# Extensive Pyrosequencing Reveals Frequent Intra-Genomic Variations of Internal Transcribed Spacer Regions of Nuclear Ribosomal DNA

**DOI:** 10.1371/journal.pone.0043971

**Published:** 2012-08-30

**Authors:** Jingyuan Song, Linchun Shi, Dezhu Li, Yongzhen Sun, Yunyun Niu, Zhiduan Chen, Hongmei Luo, Xiaohui Pang, Zhiying Sun, Chang Liu, Aiping Lv, Youping Deng, Zachary Larson-Rabin, Mike Wilkinson, Shilin Chen

**Affiliations:** 1 Institute of Medicinal Plant Development, Chinese Academy of Medical Sciences, Peking Union Medical College, Beijing, China; 2 Kunming Institute of Botany, Chinese Academy of Sciences, Kunming, Yunnan, China; 3 Institute of Botany, Chinese Academy of Sciences, Beijing, China; 4 China Academy of Chinese Medical Sciences, Beijing, China; 5 Rush University Medical Center, Chicago, Illinois, United States of America; 6 Adelaide University, Adelaide, Australia; CNR, Italy

## Abstract

**Background:**

Internal transcribed spacer of nuclear ribosomal DNA (nrDNA) is already one of the most popular phylogenetic and DNA barcoding markers. However, the existence of its multiple copies has complicated such usage and a detailed characterization of intra-genomic variations is critical to address such concerns.

**Methodology/Principal Findings:**

In this study, we used sequence-tagged pyrosequencing and genome-wide analyses to characterize intra-genomic variations of internal transcribed spacer 2 (ITS2) regions from 178 plant species. We discovered that mutation of ITS2 is frequent, with a mean of 35 variants per species. And on average, three of the most abundant variants make up 91% of all ITS2 copies. Moreover, we found different congeneric species share identical variants in 13 genera. Interestingly, different species across different genera also share identical variants. In particular, one minor variant of ITS2 in *Eleutherococcus giraldii* was found identical to the ITS2 major variant of *Panax ginseng*, both from Araliaceae family. In addition, DNA barcoding gap analysis showed that the intra-genomic distances were markedly smaller than those of the intra-specific or inter-specific variants. When each of 5543 variants were examined for its species discrimination efficiency, a 97% success rate was obtained at the species level.

**Conclusions:**

Identification of identical ITS2 variants across intra-generic or inter-generic species revealed complex species evolutionary history, possibly, horizontal gene transfer and ancestral hybridization. Although intra-genomic multiple variants are frequently found within each genome, the usage of the major variants alone is sufficient for phylogeny construction and species determination in most cases. Furthermore, the inclusion of minor variants further improves the resolution of species identification.

## Introduction

The study of evolutionary novelty focuses on morphological traits [1] as well as genomic and proteomic data [2], especially molecular sequence data [3]–[9]. Among molecular sequences, the internal transcribed spacer 2 (ITS2) of the nuclear ribosomal DNA cistron is one of the most frequently used markers for phylogenetics, diagnostics and DNA barcoding [10]–[18]. Most eukaryotes possess hundreds of tandem copies of this cistron, each consisting of the 18S, 5.8S, and 28S rRNA genes, two external transcribed spacers (ETS1 and ETS2), two internal transcribed spacers (ITS1 and ITS2), and an intergenic spacer (IGS). Among them, ITS2 can be the most informative for discrimination at the species and subspecies levels, and it contains conserved secondary structures that can be used to facilitate alignments of higher taxonomic categories (from genus to order) due to its function in rRNA processing [19]. In each genome, however, there are hundreds of copies of ITS2 with potentially dozens of different sequences, so sequence comparisons involving just one or a few PCR-amplified ITS2 sequences from each species of interest may lead to inaccurate or misleading results [20]. This largely overlooked intra-genomic divergence of ITS2 sequences can particularly complicate the use of this marker in phylogenetic and barcoding applications. Conversely, the use of all ITS2 sequences within genomes to perform phylogenetic analyses has the potential to add new sources of data; e.g., whereas traditional methods using morphological traits and single-copy molecular markers can only display the current features of a given species, the full set of the non-coding ITS2 sequences can evolve more rapidly, yielding evolutionary insights that morphology and coding-sequences could not provide because of functional constraints. In this paper we demonstrate how intra-genomic sequence variation of ITS2 can be identified and used to provide additional direct genetic evidence reflecting the historical events of species evolution.

**Figure 1:**
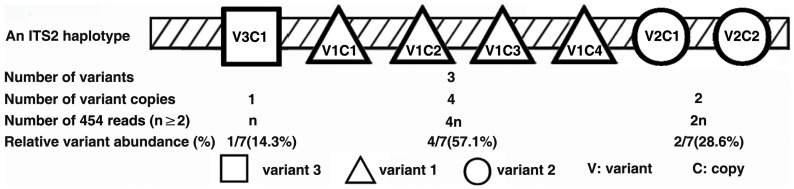
Schematic representation of an ITS2 haplotype. The variants 1–3 were sorted by their Relative Variant Abundance (RVA).

**Figure 2:**
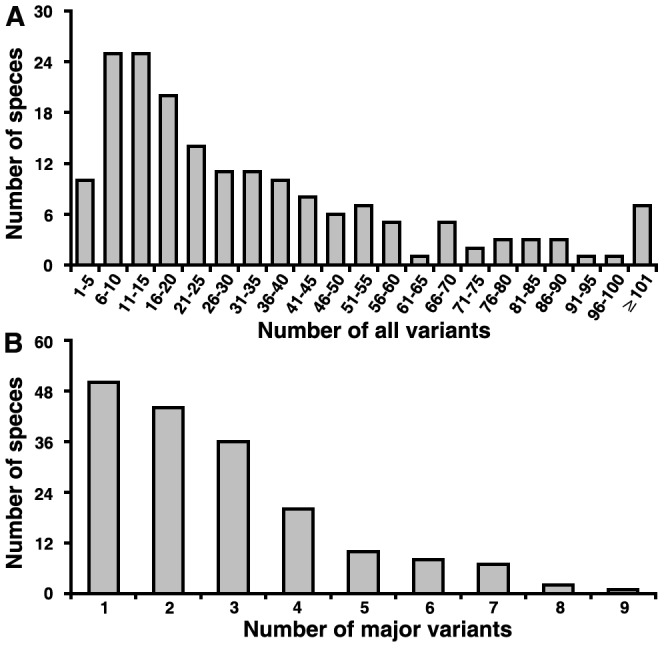
Distribution of numbers of intra-genomic ITS2 variants from 178 species. (A) Major and minor variants. (B) Major variants.

**Figure 3:**
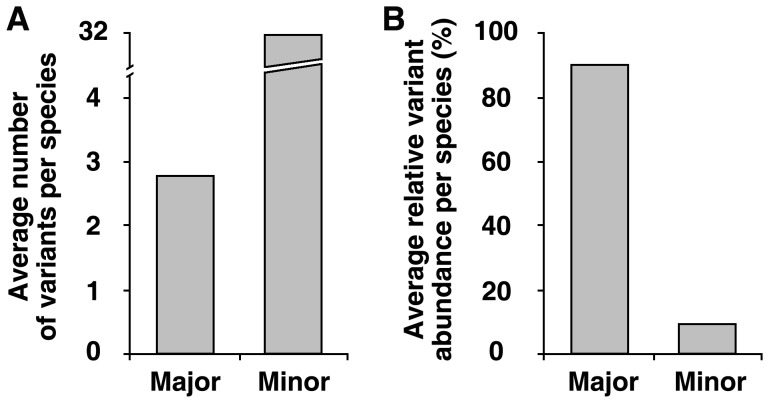
Average number of variants and average Relative Variant Abundance (RVA) per species. (A) Average number of intra-genomic variants. (B) Average RVA.

Although utilizing genomic *in situ* hybridization (GISH) and fluorescent *in situ* hybridization (FISH) techniques has revealed variations in the genomic locations and numbers of ITS2 copies in plant genomes [20], the extent of the distribution and divergence of ITS2 intra-genomic variants has not been adequately explored. Nor has the relationship between ITS2 sequence divergence and plant phylogenetics been sufficiently examined. In fact, previous experiments utilizing denaturing gradient gel electrophoresis (DGGE) and cloning techniques may have significantly underestimated intra-genomic ITS2 variation [21]. Next-generation pyrosequencing using Roche’s 454 method [22] has been used to identify genome-wide genetic changes [23] and examine taxonomic diversity [24]–[28], as well as to produce libraries of expressed sequence tags (ESTs) [29], [30]. In the present study, we performed sequence-tagged pyrosequencing to investigate the diversity of intra-genomic variants of ITS2 across a host of plant genomes, and used these variants to reevaluate some phylogenetic relationships. We confirmed the sensitivity and accuracy of sequence-tagged pyrosequencing for discovering intra-genomic ITS2 variants, by comparing our *de novo* ITS2 variants with sequences found in whole-genome sequences from *Arabidopsis thaliana* [31], *Oryza sativa* ssp. *indica* [32], *Oryza sativa* ssp. *japonica* [33], *Populus trichocarpa* [34], and *Zea mays* [35]. Our results strongly suggest that the intra-genomic ITS2 variants can reflect the phylogenetic history of species and genera.

**Figure 4:**
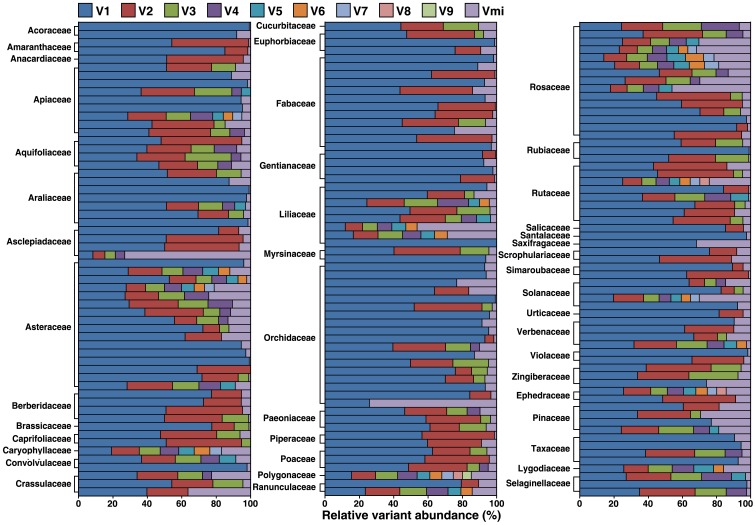
Relative variant abundance (RVA) of various ITS2 variants in plant genomes. Each marker (V1 to V9) represents RVA of a major variant sorted by their RVA while the marker Vmi represents sum of RVA for all other minor variants. The ruler shows the RVA in percentage.

## Results

### Reliability of Sequence-tagged Pyrosequencing for Discovering Intra-genomic ITS2 Variants

In this study, we obtained a mean of 2587 reads per sample, with a mean read length of 330 bases and high overall base-calling fidelity (**Figure S1**). We compared 454 reads from *Panax quinquefolius* with previously published *P. quinquefolius* sequences and found that the per-base pyrosequencing error rate was 1.29×10^−4^ (**Table S1**). However, similar to results reported by Schrijver et al. [36], homopolymeric regions of the sequences can give ambiguous results, making it difficult to determine whether so-called variants are *bona fide* variants or sequencing errors in such regions. Therefore, we suggest caution be used when evaluating variants within homopolymeric regions in the variants described below, although there is little effect on generating all of our results because of the low error rate. The terms “Number of variants”, “Number of variant copies”, “Number of 454 reads”, “Relative variant abundance (RVA)”, and “An ITS2 haplotype” are defined in **Figure 1** **.** An ITS2 haplotype in a genome contains all copies of all variants. The numbers of variants and the genetic distances between them are indicative of the divergence of ITS2 within and among plant genomes. A “major variant” is defined as any variant whose RVA is greater than 5%.

**Figure 5:**
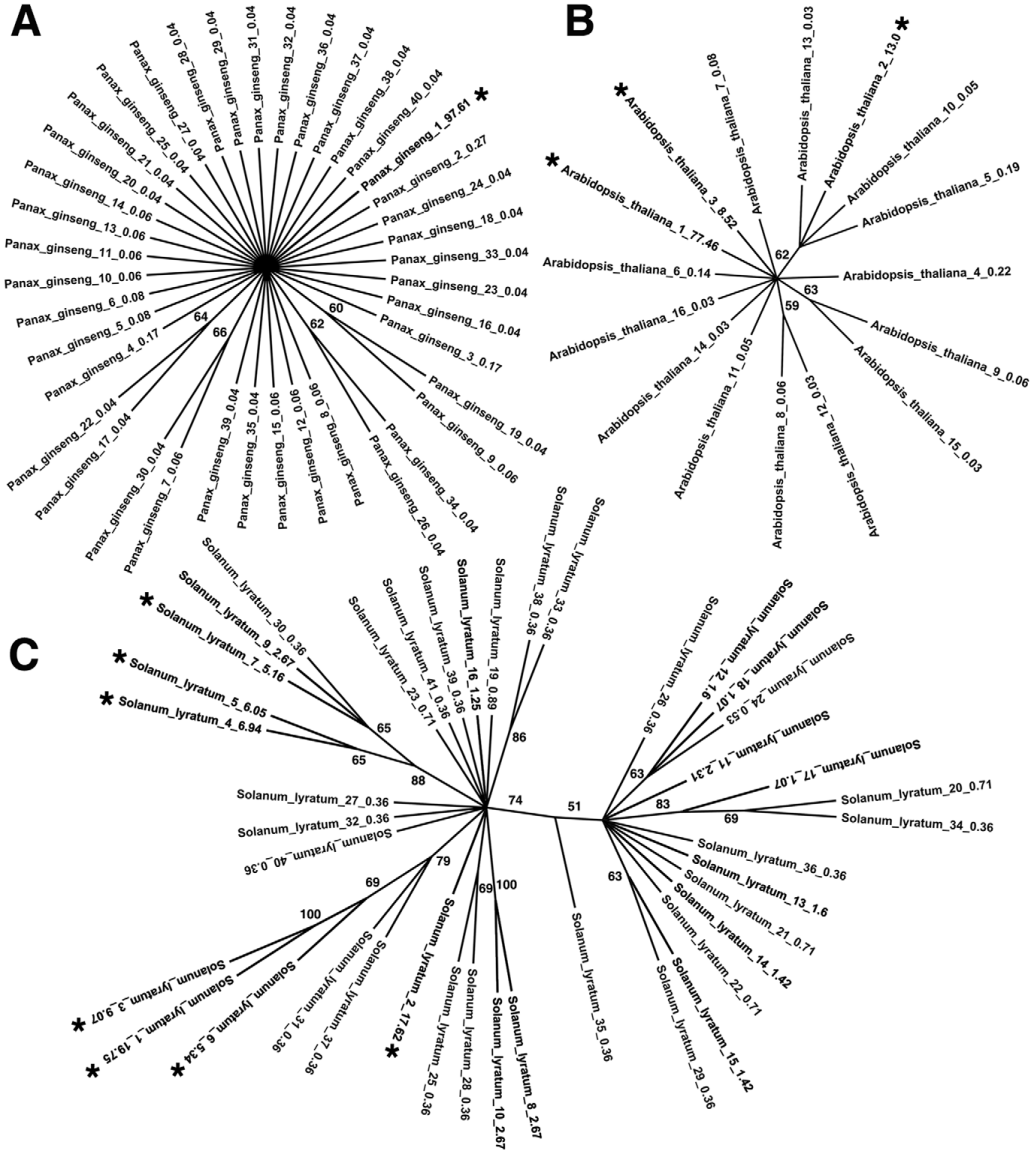
Unrooted Neighbor-Joining trees representing the results of three hypothetical mechanisms for the evolution of intra-genomic ITS2 variants. The symbols “*” show major variants. (A) The concerted evolution mechanism. *Panax ginseng* is an example, which has only one cluster of major variant. (B) The birth-and-death evolution mechanism. *Arabidopsis thaliana* is an example and shows the presence of two clusters of major variants. (C) The divergent evolution mechanism. *Solanum lyratum* is an example and shows the presence of multiple clusters of major variants.

To validate the reliability of ITS2 variants produced by pyrosequencing, we further compared the divergence of ITS2 variants identified using pyrosequencing data with data acquired from publicly available genome sequences and those obtained using the cloning method for 12 samples belonging to 5 species (*Arabidopsis*, maize, poplar, and two rice species). The results demonstrated that the sensitivity to discover minor variants of ITS2 in plant genomes using pyrosequencing technology is similar to that of whole genome sequence analysis but is far superior compared with the cloning method (**Figure S2**). The cloning method can be used to identify the major variants of ITS2 effectively, but it is ineffective in identifying minor variants even in the situation when more than one hundred clones were picked and sequenced. Therefore, pyrosequencing is a superior tool for discovering the ITS2 variants in plant genomes.

### Intra-genomic Variation of ITS2 Sequences across a Wide Range of Plant Taxa

We analyzed 247 samples from 178 plant species and discovered that these genomes have 1 to 253 different ITS2 variants, with a mean of 35 and a median of 23 (**Figure 2A**). The distribution of major variants was shown in **Figure 2B** **.** The average number of major variants per species is fewer than 3, while number of minor variants per species was 32 in a genome (**Figure 3A**). The distribution of RVA for major and minor variants in 44 families displayed divergent forms (**Figure 4**). On average, the sum of RVA of major variants per species constituted 91% of 454 reads recovered from that specieś genome (**Figure 3B**). The results reveal that mutation of ITS2 in a genome is frequent but major variants of ITS2 are conservative and predominant.

**Figure 6:**
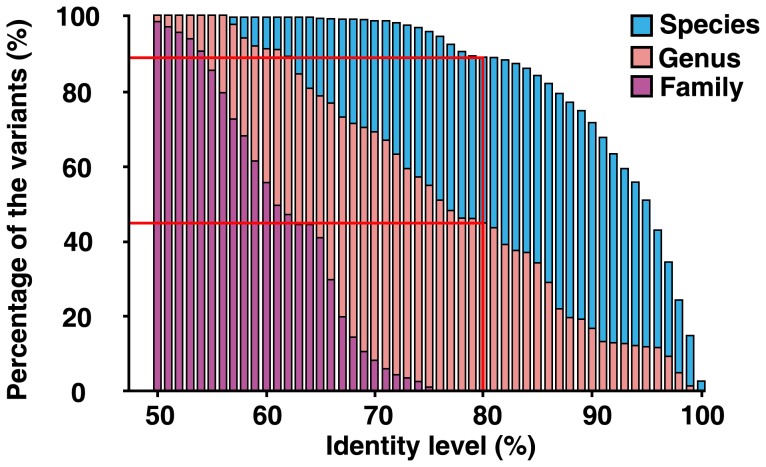
Identity of ITS2 variants in plant genomes between different taxonomic clades. “Species” represents identity between congeneric species while “Genus” represents identity between genera in the same family and “Family” represents identity between different families.

According to the phylogenetic trees of intra-genomic variants from all species under investigation, intra-genomic variation of ITS2 sequences was distributed among three established evolution mechanisms [19]. The first mechanism is concerted evolution, whereby ITS2 variants evolved as units in plant genomes by the mechanics of unequal crossing-over and gene conversion; *Panax ginseng* genome has only one cluster of major variant, in agreement with results from this mechanism (**Figure 5A** **, Figure S3A**). The second mechanism is birth-and-death evolution, in which some ITS2 variants were produced by gene duplication and a fraction of them were subsequently lost by deletions, translocations, and change in chromosome number; *Arabidopsis thaliana* genome has two clusters of major variants and is considered a demonstration of such mechanism (**Figure 5B** **, Figure S3B**). The third mechanism is divergent evolution, whereby ITS2 variants within a plant genome evolved divergently and independently; *Solanum lyratum* genome has multiple clusters of major variants and can be best explained by this mechanism (**Figure 5C** **, Figure S3C**). In our study as a whole, concerted evolution appears to be the best mechanism explaining intra-genomic variation of ITS2 sequences for 65.7% of the species, while birth-and-death evolution and divergent evolution are the best mechanism to explain 27% and 7.3% of the species, respectively (**Table S2**). Regardless of the mechanisms that give rise to ITS2 variants, the intra-genomic variants often provide clues to the evolutionary history of species.

**Figure 7:**
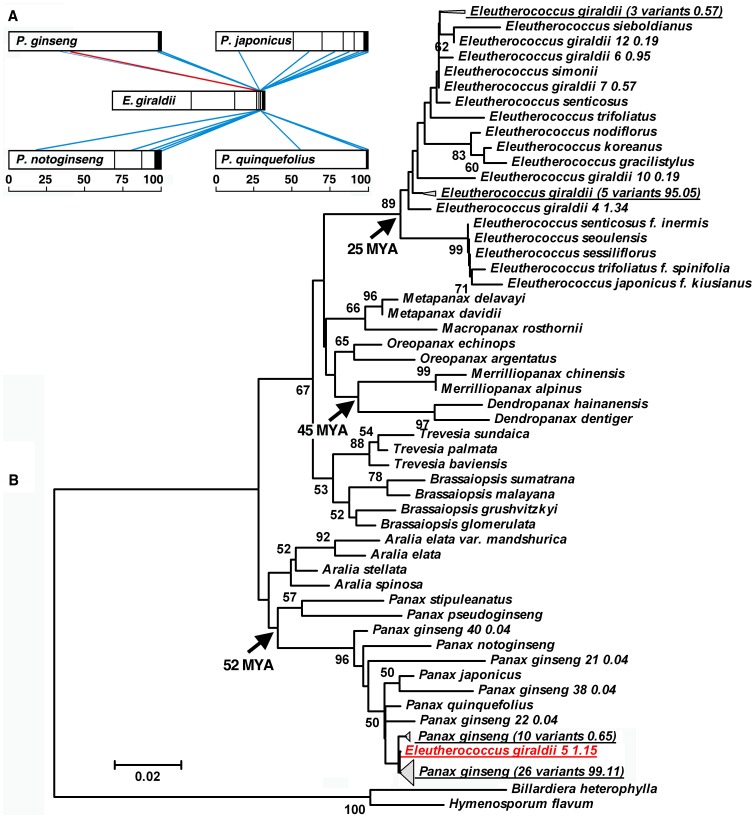
Two identical variants of ITS2 in the genera *Panax* and *Eleutherococcus* of Araliaceae. (A) Pairs of variants are linked using red and blue lines at 100% and 95% identities, respectively. Each open box represents a single variant with relative variant abundance (RVA) over 1%, and each solid box represents the variants with RVA less than 1%. The rulers show the RVA in percentage. (B) The Neighbor-Joining tree of ITS2 in Araliaceae. Numbers are bootstrap values (<50% not shown). One minor variant of ITS2 in *Eleutherococcus giraldii* showed close affinity to the ITS2 variants of *Panax ginseng* and was clustered with them. Based on a fossil calibration of the split between *Dendropanax* and *Merrilliopanax* at 45 million year ago (MYA), divergence dates among *Panax* species and among *Eleutherococcus* species were estimated o be 52 MYA and 25 MYA, respectively.

### Identification of Identical Variants across Species Reflects the Complex Species Evolutionary History

Based on genome-wide analyses of 247 samples from 178 species of angiosperms, gymnosperms and ferns, 88.8% of ITS2 variants possessed more than 80% identity among congeneric species, while only 45.5% of ITS2 variants possessed more than 80% identity among genera in the same family, but no ITS2 variants showed more than 80% identity among all tested families (**Figure 6**). ITS2 variants in plant genomes displayed varying degrees of similarity within most of plant taxa. In particular, some inter-specific or inter-generic ITS2 variants showed high degrees of sequence similarity, while others showed low degrees of sequence similarity. Remarkably, some inter-specific or inter-generic variants were 100% identical and their abundance varied. Such identical or highly-similar ITS2 variants can reflect the evolutionary history of the species in a manner different from the analysis of single-copy genes, which exhibit only the current characters of the species. From analyses of phylogenetic trees and genetic distances, these shared ITS2 variants within different plant taxa display closer phylogenetic relationships to each other than to those with other ITS2 variants.

For example, in the family Araliaceae, one minor variant of ITS2 in *Eleutherococcus giraldii* showed 100% identity to the ITS2 major variant of *Panax ginseng* (**Figure 7A,B** **, Figure S4A**). The variant was validated in the nuclear genomes of *P. ginseng* and *E. giraldii* through direct sequencing of PCR products using species-specific primers (**Figure S4B**). Based on 7.4×10^−10^ substitutions/site/year of ITS2 in Araliaceae, divergence dates among *Panax* species and among *Eleutherococcus* species were estimated to be 52 million year ago (MYA) and 25 MYA, respectively (**Figure 7B**). It should be pointed out that the bootstrap values for these nodes are less than 50%, which probably resulted from the limited polymorphic sites between samples. However, this phylogenetic relationship is consistent with those described previously [37]. These results indicated that the intra-genomic variants could reveal evidences for horizontal gene transfer or an ancestral hybridization event. In addition, intra-generic similar ITS2 variants were discovered in the genus *Armeniaca* (Rosaceae). Six pairs of variants derived from *A. vulgaris* and *A. sibirica* showed 100% identity, and all ITS2 variants showed more than 95% identity (**Figure S5A**). These data and the phylogenetic trees of ITS2 variants (**Figure S5B,C**) supported the hypothesis that identical variants derived from a common ancestor and were preserved in the genomes of the descendants. The variants were corroborated by direct Sanger-sequencing or cloning of PCR products (**Figure S5D-H**). As illustrated in these NJ and MP trees, the ITS2 variants reflected the close relationships among species within a given genus and can be regarded as molecular fossils, which are defined as any molecule whose contemporary structure or function gives clues about its evolutionary history [38]. Such fossils were also discovered in 12 other genera, including *Epimedium* (Berberidaceae), *Inula* (Asteraceae), *Ipomoea* (Convolvulaceae), *Panax* (Araliaceae), and *Pinus* (Pinaceae) (**Figure S6**), and NJ trees and MP trees of the ITS2 variants have supported the close relationships among species of these genera (**Figure S7**). Determination of these ITS2 variants by the direct sequencing or cloning of PCR products is as shown in **Table S3**.

However, such molecular fossils were not discovered in all species under investigation. For example, among the four species we investigated from the genus *Ilex* (Aquifoliaceae) (*I. asprella*, *I. cornuta*, *I. pubilimba*, and *I. rotunda*) all ITS2 variants were clearly clustered within species, with high bootstrap values (**Figure S8**). Other genera for which ITS2 molecular fossils were not found include *Astragalus* (Fabaceae), *Clerodendrum* (Verbenaceae), and *Euphorbia* (Euphorbiaceae) (**Figure S9**). In the fact as described bellow, any of the variants could accurately distinguish these species. Overall, both major and minor variants in plant genomes were valuable markers for illustrating the evolutionary histories of many of the investigated species.

### Effects of Intra-genomic ITS2 Variation on DNA Barcoding Studies

In order to determine the effect of intra-genomic ITS2 variation on DNA barcoding, only the genus having at least two species was selected to characterize intra-genomic, intra-specific, and inter-specific divergences. In total, 5543 variants of ITS2 from 207 samples of 153 species in 51 genera of 34 families were analyzed. Our results indicated that 97% of all ITS2 variants could be correctly identified at the species level, while 92% of ITS2 major variants produced correct identification (**Table 1**). Among 8 of 51 genera, identification efficiency of all intra-genomic variants at the species level is higher than that of major variants with the range 8% to 66% (**Table 2**). Only among 5 of 51 genera, identification efficiency of all intra-genomic variants at the species level is lower than that of major variants with the range 1% to 3%. However, among 38 of 51 genera, identification efficiency of all intra-genomic variants at the species level is the same as that of major variants with 100%. Even if for 18 species of the genus *Dendrobium*, intra-genomic 440 variants could correctly identify each species (**Table 2**). These results show that the ability of species identification increases when full set of ITS2 variants was considered.

**Table 1:** Identification efficiency at species level for ITS2 variants from 207 samples of 153 species in 51 genera using best BLAST hit.

Variant type	Level	No. of variants	Successful identification (%)	Ambiguous identification (%)
Major	Species	531	92.3	7.7
	Genus	531	100	0
Major+Minor	Species	5543	97	3
	Genus	5543	99.9	0.1

**Table 2:** Identification efficiency at species level for ITS2 variants among each of 51 genera using best BLAST hit.

Genus	No. of species	No. of major variants	No. of all variants	Identification of major variants (%)	Identification of all variants (%)
*Armeniaca*	2	20	114	70	81.6
*Brucea*	2	5	29	40	89.7
*Cerasus*	3	18	86	55.6	66.3
*Citrus*	5	38	222	81.6	89.6
*Epimedium*	3	7	156	14.3	80.1
*Ligusticum*	3	17	121	64.7	90.9
*Pinus*	4	11	459	81.8	98.3
*Torreya*	4	7	170	57.1	91.8
*Alpinia*	3	13	276	100	97.8
*Inula*	3	7	95	100	97.9
*Ipomoea*	2	7	71	100	97.2
*Panax*	4	15	186	100	98.9
*Potentilla*	4	19	284	100	96.8
*Acorus*	2	5	24	100	100
*Amygdalus*	2	8	31	100	100
*Angelica*	3	8	144	100	100
*Ardisia*	2	4	88	100	100
*Artemisia*	5	25	163	100	100
*Asparagus*	3	16	104	100	100
*Aster*	2	8	85	100	100
*Astragalus*	3	4	65	100	100
*Celosia*	2	4	17	100	100
*Cimicifuga*	2	12	136	100	100
*Cirsium*	2	19	128	100	100
*Clerodendrum*	3	6	202	100	100
*Cynanchum*	3	6	59	100	100
*Datura*	2	7	32	100	100
*Dendrobium*	18	39	440	100	100
*Eleutherococcus*	3	5	49	100	100
*Ephedra*	2	16	61	100	100
*Euphorbia*	3	7	65	100	100
*Flemingia*	2	4	74	100	100
*Gentiana*	4	7	56	100	100
*Ilex*	4	21	85	100	100
*Lilium*	2	12	180	100	100
*Lonicera*	2	6	18	100	100
*Oryza*	2	10	26	100	100
*Paeonia*	3	13	94	100	100
*Piper*	2	5	43	100	100
*Pueraria*	2	3	32	100	100
*Rosa*	2	4	25	100	100
*Rubus*	2	4	27	100	100
*Sedum*	3	10	357	100	100
*Selaginella*	3	10	86	100	100
*Senna*	2	5	30	100	100
*Siegesbeckia*	2	5	15	100	100
*Solanum*	2	8	45	100	100
*Sophora*	3	4	101	100	100
*Uncaria*	3	10	39	100	100
*Veronicastrum*	2	4	27	100	100
*Viola*	2	3	21	100	100

**Figure 8:**
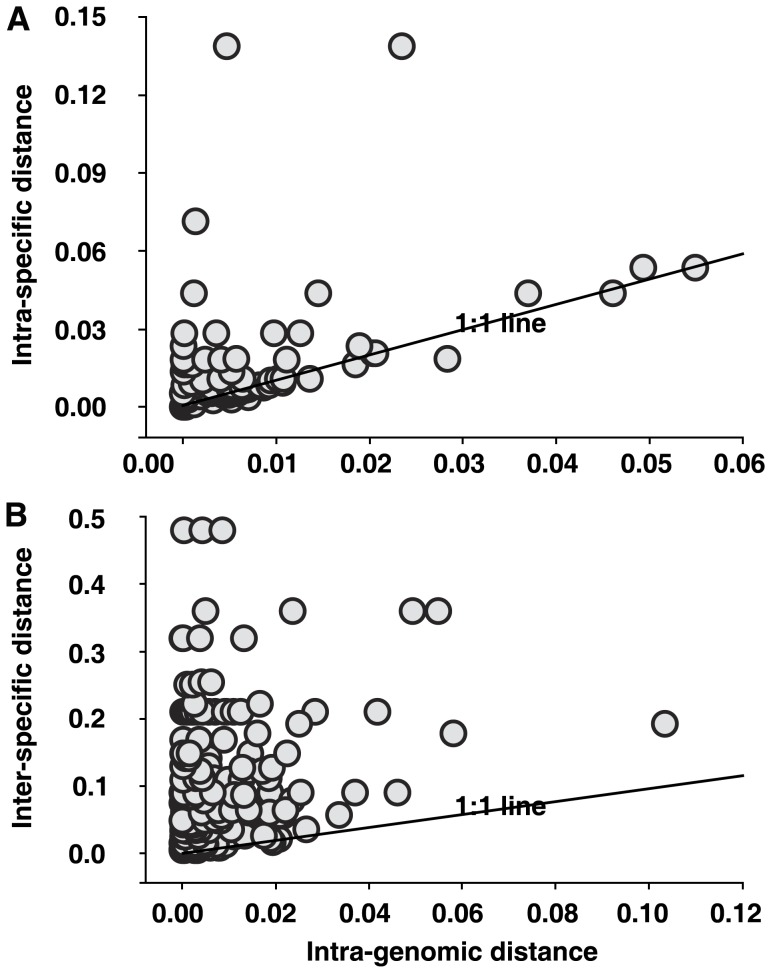
The presence/absence of barcode gaps. (A) Comparison of the genetic distances for intra-genomic and intra-specific ITS2 variants (95 dots total). (B) Comparison of the genetic distances for intra-genomic and inter-specific ITS2 variants (207 dots total).

As a result, we investigated further whether DNA barcoding gap is present. DNA barcoding gap exposed that the intra-genomic distances were markedly less than those of the intra-specific or inter-specific variants (**Figure 8**). These results indicated that the distribution for all ITS2 variants could be used to distinguish the species. Previously, the existence of a few major variants in plant genomes has allowed ITS2 to be used as a molecular marker in phylogenetics and barcoding. Although this use of ITS2 was successful in the sense that the major sequence or sequences have been used to distinguish species, the full capacity of the ITS2 variant set, including its minor variants, was overlooked.

## Discussion

Nuclear ribosomal DNA sequence variation in 34 strains of *Saccharomyces cerevisiae* and in 12 *Drosophila* species has been previously investigated by whole-genome shotgun sequencing [39], [40]. However, no attempts have been made to evaluate the overall diversity of nrDNA sequence variation across a wide range of plant species because of obstacle in detectability. To our knowledge, this study represents the first quantitative analysis of genome-scale sequence variation of ITS2 in 178 plant species using the 454-pyrosequecing method. We discovered that in a wide range of plant taxa including those of the model plant species (*Arabidopsis*, maize, poplar, and two rice species), mutation of ITS2 is frequent with a mean of 35 variants per species. This abundance of mutations provides resources for natural selection and species evolution, supporting the hypothesis that multiple copies of ITS sequences may prove to be highly informative in explaining phylogenetic history [41].

The evolutionary progress of intra-genomic ITS2 variants generally revealed the phylogenetic histories of species. Even if the phylogenetic relationship of *Panax* revealed by its ITS2 variants was in agreement with previous reports [42], [43], the affinity between *Panax* and *Eleutherococcus* reflected by the identical variant was a substantial finding that could not have been as demonstrated by single-copy gene analysis alone. Since the ITS2 variant was major in *P. ginseng* but minor in *E. giraldii*, it may have been integrated into *E. giraldii* genome from the *P. ginseng* genome through horizontal gene transfer or an ancestral hybridization event. Recently, the observation of ITS variants in the Musaceae enables determination of hybrid origin [44]. The identical ITS2 variants shared for *A. vulgaris* and *A. sibirica* provide direct genetic information that reveals close phylogenetic relationships under-reported in previous research [45]–[47]. The results may imply that multiple gene families such as 5S RNA genes also share ancestral polymorphism [48]. However, some plant taxa do not contain such ITS2 variants. One possible explanation is that the concerted evolution of multiple repeat arrays had already taken place in these species. Another possible reason is that sufficiently closely related species from those genera were not included in our study; a further analysis including more of the closely related species would test that possibility.

Based on the analysis of a large sample size, intra-genomic variants of ITS2 sequences proved to be powerful for species identification and provide a significant contribution to the field of DNA barcoding technology, which has recently attracted widespread attention [49]–[53]. Moreover, our results are important because they resolve the controversy about the internal transcribed spacer (ITS); i.e., the multicopy nature of ITS1 and ITS2 sequences has reduced their utility in phylogenetic analysis for many years [20], and more recently has rendered them unacceptable for use as standard barcodes across the plant kingdom [54]–[56]. On the other hand, many researchers have insisted that ITS2 be the best marker for phylogenetics and for barcoding [10], [12], [14], [16], [57]–[61]. Our results provide effective and direct evidence for the intra-genomic divergence of ITS2, and illustrate how ITS2 variants can yield insights into DNA barcoding. In addition, the analytical protocol we have established may enable the construction of an ITS2-based DNA barcode database, a valuable resource for investigating on ITS2 secondary structures, compensatory base changes (CBC), and etc [62]–[67]. Previous study has revealed that a CBC in an ITS2 sequence-structure alignment is a sufficient condition to distinguish even closely related species [68]. This CBC study focused on the sequences of the ITS2 major variants. Our study further characterizes the distribution and utilities of multiple ITS2 variants. Combining these results together, it supports the notion that ITS2 possesses a unique capacity to differentiate closely related species [12], [14], [59], [61], [69]; at the same time it also suggests that intra-genomic variation is less likely to compromise the vast majority of barcoding applications (e.g. that use direct sequencing) as previously reported [54]–[56]. This finding supports the use of ITS2 as a valuable supplementary locus for the standard plant barcode of *rbcL*+*matK* [50], [70].

In summary, intra-genomic ITS2 variants represent a valuable source that help reveal the evolutionary histories of related species, and the major variants of ITS2 can be useful in phylogenetic and barcoding applications.

## Materials and Methods

### DNA Samples and PCR Conditions

For the 5 species (maize, Arabidopsis, poplar, and two rice species) with publicly available whole genome sequences, *in vitro* cultured sterile plantlets were used for DNA extraction. Procurement of materials from other plant species was described previously [14], [71], [72]. Genomic DNA extraction was performed using the Plant Genomic DNA Extraction Kit according to the manufacturer′s protocol (Tiangen Biotech Co., China). ITS2 sequences were obtained from 247 samples from 178 species of 76 genera belonging to 44 families of angiosperms, gymnosperms, and ferns (**Table S4**). The plant materials were mainly selected based on the list in Chinese Pharmacopoeia, which possess medical importance. In addition, we took particular interests in those that are also economically important species, such as *Panax ginseng*, *Dendrobium nobile* and etc. To distinguish each PCR product, unique sequence-tagged PCR primer pairs were designed for each sample (**Table S5**). Each 25 µL PCR mixture contained ∼30 ng of template DNA, 2.5 µL of 10×PCR buffer, 1 µL of 2.5 mM dNTPs mix, 0.5 U of High-fidelity Pyrobest ® DNA Polymerase (Takara Biotech Co., China), and 1.0 µL of 2.5 µM ITS2 primers (synthesized by Shanghai Sangon Biotech Co. Ltd, China). The PrimeSTAR® HS DNA Polymerase with GC Buffer (Takara Biotech Co., China) was used for *Oryza sativa* ssp. *japonica*, *Oryza sativa* ssp. *indica* and *Populus trichocarpa,* since the GC content of these ITS2 sequences is ca. 70%. PCR runs were performed using a BIO-RAD DNA Engine® Peltier Thermal Cycler with near-universal programs (**Table S6**). In addition, three PCR products from *Panax quinquefolius* with known sequences of lengths 176 bp, 187 bp, and 350 bp (GenBank accessions: JF927163, JF927167, JF927168) were used as internal controls to measure the sequencing accuracy of pyrosequencing.

### 454 Pyrosequencing and Cloning

The PCR products were purified using a TIANquick Midi Purification Kit (Tiangen Biotech Co., China) and dissolved in 30–50 µL of 10 mM Tris-HCl (pH 8.0). The concentration and purity of the PCR products were examined using a NanoDrop ND-1000 Spectrophotometer (Thermo Scientific, USA). 170 ng of each PCR product was mixed, then concentrated in less than 500 µL of 10 mM Tris-HCl (pH 8.0) using an Amicon Ultra-0.5 mL 30 kDa membrane (Millipore, USA). The mixture was sequenced in separate half-lanes of a picotiter plate using a GS_FLX pyrosequencer according to the manufacturers’ instructions. The raw 454 reads were submitted to GenBank with accession number SRA037432.

To confirm that our methods were sensitive enough to discover intra-genomic ITS2 variants and to further validate the accuracy of sequence data produced by the 454 pyro-sequencing, 12 samples belonging to 5 species with sequenced genomes were analyzed using PCR amplification, cloning and Sanger DNA sequencing methods. The PCR products were examined by 1% agarose gel electrophoresis and recovered using an agarose gel recovering kit (Tiangen Biotech Co., China). The recovered products were then ligated to pMD® 18-T Vector (Takara Biotech Co., China) after adding dATP at the 3′end of the PCR products using a DNA A-Tailing Kit (Takara Biotech Co., China) and transformed into the *E. coli* DH5α strain using standard recombinant DNA techniques [73]. Identification of the transformed bacteria was performed by PCR using M13 primers. For each sample, 24 to 120 PCR-identified positive clones were subjected to direct sequencing using a 3730XL DNA Analyzer (ABI, USA).

### Data Analyses

The obtained pyrosequencing reads were analyzed according to the work flow diagram (**Figure S10**). First, all 454 sequence reads were assigned to the original samples according to perfect matches with the sequence-tagged PCR primers. The distributions of read number, read length, number of reads from forward or reverse strands, and base quality were calculated and evaluated. Second, we used the algorithm to reduce pyrosequencing noise [27]. Third, the ITS2 sequences from a subset of investigated species were obtained by bi-direction Sanger sequencing of PCR products, or directly downloaded from GenBank, as standard query sequences which was annotated using hidden Markov models (HMM). A database of full-length ITS2 sequences from the investigated species was compiled using our full-length-match ITS2 pyrosequencing reads (if we obtained more than 50 reads from that sample), and sequences obtained according to standard query sequences using BLAST hits with E-values less than 10^−20^. Fourth, the variants of intact ITS2 regions supported by at least two pyrosequencing reads were selected for further analysis. All ITS2 variants were then sorted using scripts written in the Python programming language. Fifth, sequence alignment for the ITS2 variants was performed using MUSCLE (version 3.6, [74] ) and further adjusted manually using Jalview (version 2.7, [75] ). K2P genetic distances [76] were computed using PAUP 4b10 [77]. Sixth, Neighbor-Joining (NJ) and Maximum Parsimony (MP) trees were constructed with PAUP 4b10. Based on a fossil calibration of the split between *Dendropanax* and *Merrilliopanax* at 45 MYA [37], the substitution rate of ITS2 in Araliaceae was calculated to be 7.4×10^−10^ substitutions/site/year, which is in agreement with nrITS’s range of substitution rate (from 3.8×10^−10^ to 8.34×10^−9^) [78]. Names and GenBank accession numbers of the other 37 previously published ITS2 sequences from Araliaceae and two outgroup taxa were listed in **Table S7**. Seventh, based on the ITS2 variants, species identification was carried out using BLAST1 method as described previously by Ross et al. [79]. Finally, identity analyses between pairs of variants were performed using the pairwise alignment function of MUSCLE software v. 3.6, and the identities between congeneric species, between genera in the same family, and between different families were computed using custom scripts.

In addition, the whole genome sequence data for the five model species were downloaded from the Tair-AGI genomic database (*Arabidopsis thaliana*), the NCBI Trace Archive (*Zea mays*, *Oryza sativa* ssp. *indica*, *Oryza sativa* ssp. *japonica*), and JGI (*Populus trichocarpa*). The ITS2 regions were extracted using the procedures described above. The ITS2 sequences with base quality scores greater than 20, or those with variants that occurred more than two times, were used for further analysis.

### Verification of the Identical ITS2 Variants Derived from Different Species

According to the sequences of the identical ITS2 variants obtained from the 454 reads, specific primer pairs for the corresponding samples were designed (**Table S8**). The PCR cycling regimes and reaction conditions were shown in **Table S9**. The cloning of PCR products was performed following the protocols described above. The PCR cycling regimes and reaction conditions for the ITS2 variants that had been confirmed by PCR with the universal primers were described in a previous study [14].

## Supporting Information

Figure S1 Overview of the 454 sequencing data. (**A**) Distribution of read number among the investigated samples. (**B**) Distribution of read lengths in the investigated samples. (**C**) Distribution of sense vs. antisense reads in the investigated samples. (**D**) Distribution of base quality. The base quality was low for homopolymer lengths greater than 3 bases.
(PDF)

Figure S2 Comparison of ITS2 variants from completed genome sequence data, cloning data and 454 pyrosequencing data. For each species, an unrooted Neighbor-Joining tree shows the diversity of ITS2 variants. Each branch represents an ITS2 variant, labeled with the sample number followed by the rank of the variant, RVA of the variant, and the letter (G, C or F). The letter G indicates a variant derived from completed genome sequence data. The letter C represents a variant derived from cloning data. The letter F represents a variant derived from 454 data. (**A**) Unrooted tree of ITS2 variants in *Arabidopsis thaliana*. (**B**) Unrooted tree of ITS2 variants in *Oryza sativa* ssp. *japonica*. (**C**) Unrooted tree of ITS2 variants in *Oryza sativa* ssp. *indica*. (**D**) Unrooted tree of ITS2 variants in *Populus trichocarpa*. (**E**) Unrooted tree of ITS2 variants in *Zea mays*.
(PDF)

Figure S3 Unrooted Maximum Parsimony trees displaying the evolutionary mechanisms of intra-genomic ITS2 variants in plant genomes. (**A**) The concerted evolution mechanism. *Panax ginseng* is used as an example. The red line indicates the most major variant. (**B**) The birth-and-death evolution mechanism. *Arabidopsis thaliana* is used as an example. The red lines indicate the two main variant clusters. (**C**) The divergent evolution mechanism. *Solanum lyratum* is used as an example. The red lines indicate multiple clusters of variants.
(PDF)

Figure S4 Two identical variants of ITS2 in the genera *Panax* and *Eleutherococcus* of Araliaceae. (**A**) The Maximum Parsimony (MP) tree of ITS2 in Araliaceae. One minor variant of ITS2 in *Eleutherococcus giraldii* showed close affinity to the ITS2 variants of *Panax ginseng* and was clustered with them. (**B**) This ITS2 variant, which was the major variant in the *Panax ginseng genome* but minor in *Eleutherococcus giraldii*, was confirmed by direct sequencing of PCR products using specific primers. The sequencing trace from *E. giraldii* shows double peaks at some bases, and these double peaks correspond to the major ITS2 variant in *P. ginseng*. The bases having double peaks are boxed in red.
(PDF)

Figure S5 ITS2 variants representing molecular fossils in the genus *Armeniaca* (Rosaceae). (**A**) The variants are linked using red and blue lines at 100% and 95% identities, respectively. Each open box represents a single variant with RVA over 1%, and each solid box represents the variants with RVA less than 1%. The ruler shows the RVA in percentage. (**B,C**) The Neighbor-Joining (NJ) and Maximum Parsimony (MP) trees of ITS2 variants indicated that genetic information from the common ancestor was maintained in the genomes of two species derived from *Armeniaca* (Rosaceae). The same colors represent the same species. The same shade and color of symbols show the variants with 100% identity. The Latin names of species are followed by the rank and RVA of the variants. (**D**) The ITS2 variants in *A. vulgaris* were confirmed by direct sequencing of PCR products. The sequencing trace from *A. vulgaris* shows double peaks at some bases, and these double peaks correspond to the different ITS2 variants in *A. vulgaris*. The bases with double peaks are boxed in red. (**E-H**) The variants as molecular fossils of *A. sibirica* were confirmed by cloning of PCR products.
(PDF)

Figure S6 The variants are linked using red and blue lines at 100% and 95% identities, respectively. Each open box represents a single variant with RVA over 1%, and each solid box represents the variants with RVA less than 1%. The rulers show the RVA in percentage. (**A**) *Epimedium* (Berberidaceae). (**B**) *Inula* (Asteraceae). (**C**) *Ipomoea* (Convolvulaceae). (**D**) *Panax* (Araliaceae). (**E**) *Pinus* (Pinaceae).
(PDF)

Figure S7 The Neighbor-Joining and the Maximum Parsimony trees of ITS2 variants indicated that genetic information from the common ancestor was maintained in the genomes of descendants. The same colors represent the same species. The same shade and color of symbols show the variants with 100% identity. The Latin names of species are followed by the rank and RVA of the variants. (**A,B**) *Epimedium* (Berberidaceae). (**C,D**) *Inula* (Asteraceae). (**E,F**) *Ipomoea* (Convolvulaceae). (**G,H**) *Panax* (Araliaceae). (**I,J**) *Pinus* (Pinaceae).
(PDF)

Figure S8 The Neighbor-Joining tree of ITS2 variants of four species in the genus *Ilex* (Aquifoliaceae). The same colors represent the same species. The Latin names of species are followed by the rank and RVA of the variants.
(PDF)

Figure S9 The Neighbor-Joining tree of ITS2 variants in the genera. (**A**) *Astragalus* (Fabaceae), (**B**) *Clerodendrum* (Verbenaceae), and (**C**) *Euphorbia* (Euphorbiaceae). The same colors represent the same species. The Latin names of species are followed by the rank and RVA of the variants.
(PDF)

Figure S10 The work flow for the processing and analysis of pyrosequencing reads.
(PDF)

Table S1 Determining the pyrosequencing error rate using known *Panax quinquefolius* sequences as internal controls.
(PDF)

Table S2 The evolutionary mechanisms of ITS2 variants across 178 plant species under investigation.
(PDF)

Table S3 ITS2 variants which have been verified by PCR products cloned or directly sequenced.
(PDF)

Table S4 Samples and their voucher numbers.
(PDF)

Table S5 Sequence-tagged PCR primer pairs for each sample.
(PDF)

Table S6 PCR cycling regime for primers in **Table S5**.
(PDF)

Table S7 GenBank accession numbers of the 37 previously published ITS2 sequences from Araliaceae and two outgroup taxa.
(PDF)

Table S8 Specific primer pairs for the corresponding species.
(PDF)

Table S9 The PCR cycling and reaction conditions for specific primer pairs in **Table S8**.
(PDF)
