# Supplementary material for: Extensive Pyrosequencing Reveals Frequent Intra-Genomic Variations of Internal Transcribed Spacer Regions of Nuclear Ribosomal DNA
Source: PLoS One. 2012 Aug 30;7(8):e43971. doi: 10.1371/journal.pone.0043971 (PMC3431384; doi:10.1371/journal.pone.0043971)

Figure S1. Overview of the 454 sequencing data **(A)** Distribution of read number among the investigated samples. **(B)** Distribution of read lengths in the investigated samples. **(C)** Distribution of sense vs. antisense reads in the investigated samples. **(D)** Distribution of base quality. The base quality was low for homopolymer lengths greater than 3 bases.

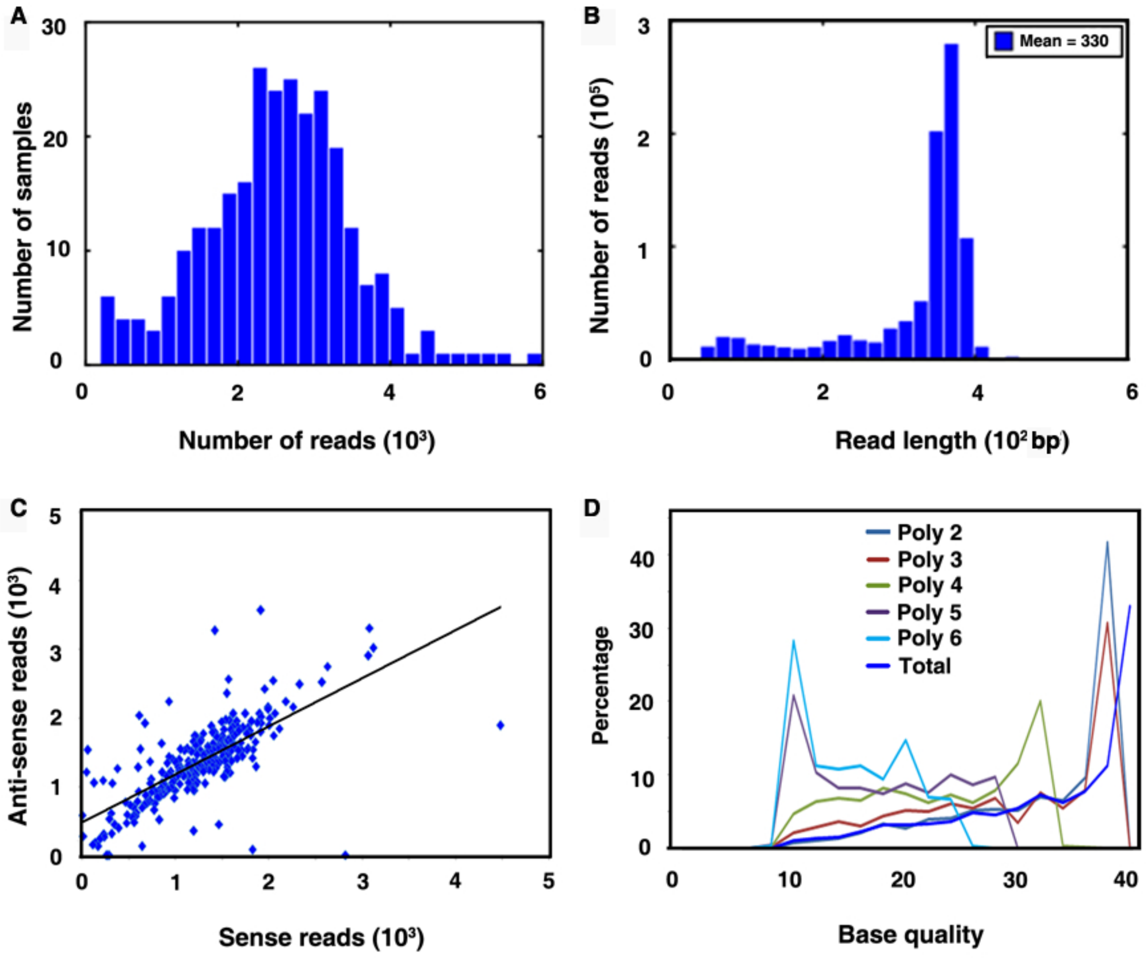

Supplement: Figure S1 — Overview of the 454 sequencing data. (A) Distribution of read number among the investigated samples. (B) Distribution of read lengths in the investigated samples. (C) Distribution of sense vs. antisense reads in the investigated samples. (D) Distribution of base quality. The base quality was low for homopolymer lengths greater than 3 bases. (PDF) [file pone.0043971.s001.pdf]
