# Supplementary material for: Extensive Pyrosequencing Reveals Frequent Intra-Genomic Variations of Internal Transcribed Spacer Regions of Nuclear Ribosomal DNA
Source: PLoS One. 2012 Aug 30;7(8):e43971. doi: 10.1371/journal.pone.0043971 (PMC3431384; doi:10.1371/journal.pone.0043971)

Figure S2. Comparison of ITS2 variants from completed genome sequence data, cloning data and 454 pyrosequencing data. For each species, an unrooted Neighbor-Joining tree shows the diversity of ITS2 variants. Each branch represents an ITS2 variant, labeled with the sample number followed by the rank of the variant, RVA of the variant, and the letter (G, C or F). The letter G indicates a variant derived from completed genome sequence data. The letter C represents a variant derived from cloning data. The letter F represents a variant derived from 454 data. **(A)** Unrooted tree of ITS2 variants in *Arabidopsis thaliana*. **(B)** Unrooted tree of ITS2 variants in *Oryza sativa* ssp. *japonica*. **(C)** Unrooted tree of ITS2 variants in *Oryza sativa* ssp. *indica*. **(D)** Unrooted tree of ITS2 variants in *Populus trichocarpa*. **(E)** Unrooted tree of ITS2 variants in *Zea mays*.

Figure S2A.

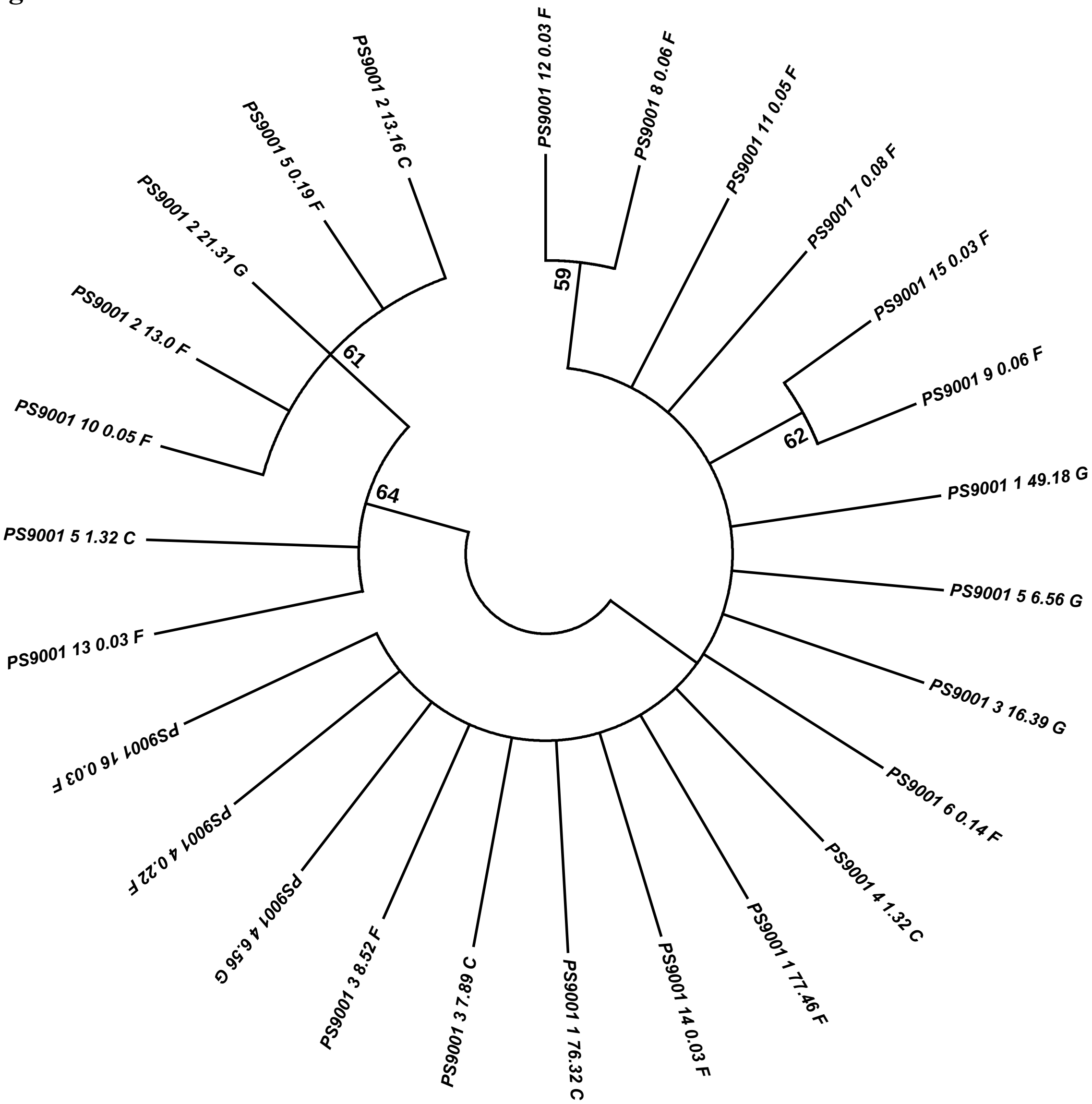

Figure S2B.

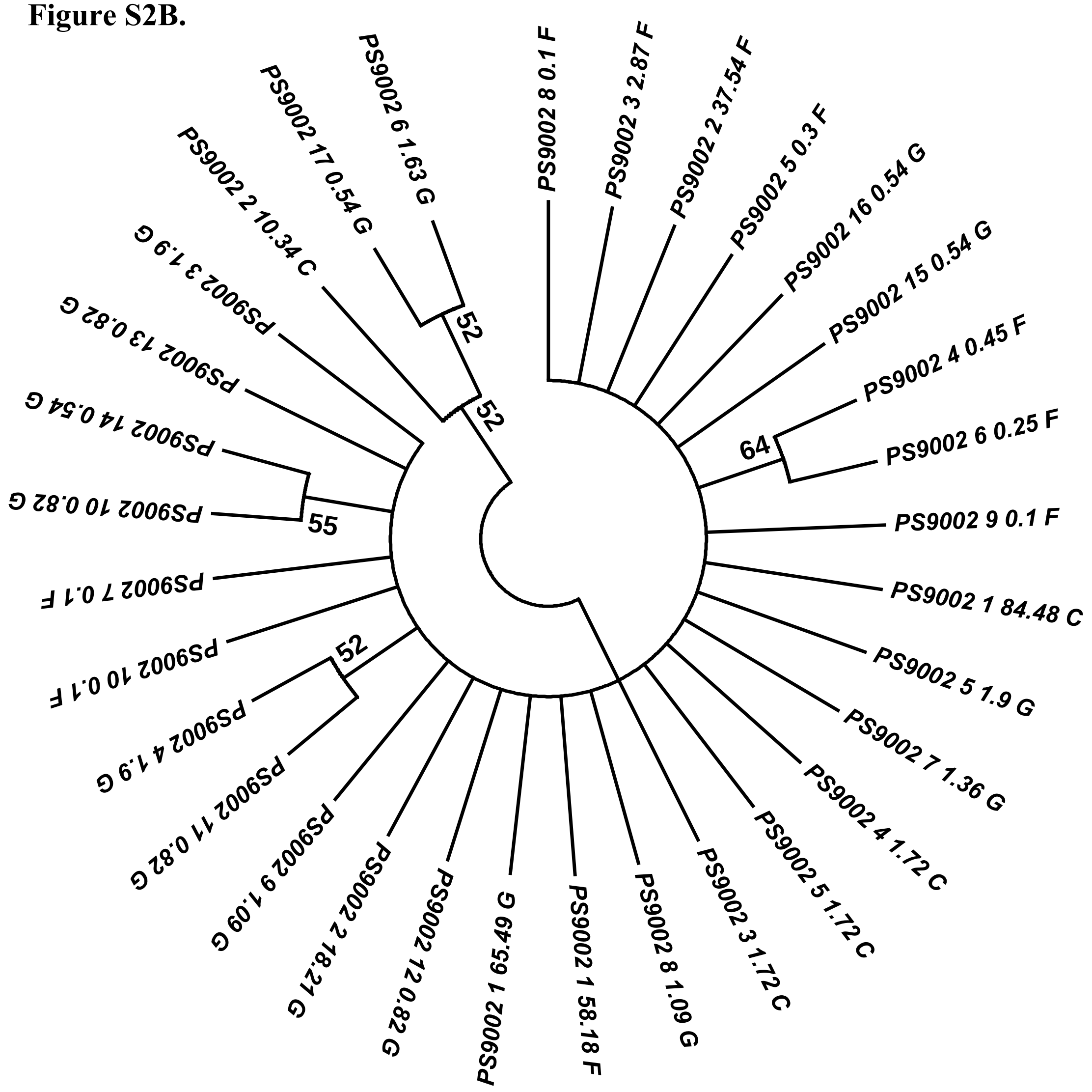
PS9002 8 0.1 F
PS9002 3 2.87 F
PS9002 2 37.54 F
PS9002 5 0.3 F
PS9002 16 0.54 G
PS9002 15 0.54 G
PS9002 4 0.45 F
PS9002 6 0.25 F
64
PS9002 9 0.1 F
PS9002 1 84.48 C
PS9002 5 1.9 G
PS9002 7 1.36 G
PS9002 4 1.72 C
PS9002 5 1.72 C
PS9002 3 1.72 C
PS9002 8 1.09 G
PS9002 1 58.18 F
PS9002 1 65.49 G
PS9002 12 0.82 G
PS9002 2 18.21 G
PS9002 9 1.09 G
PS9002 11 0.82 G
PS9002 4 1.9 G
52
PS9002 10 0.1 F
PS9002 7 0.1 F
PS9002 10 0.82 G
55
PS9002 14 0.54 G
PS9002 13 0.82 G
PS9002 3 1.9 G
PS9002 2 10.34 C
PS9002 17 0.54 G
PS9002 6 1.63 G
52
52

Figure S2C.

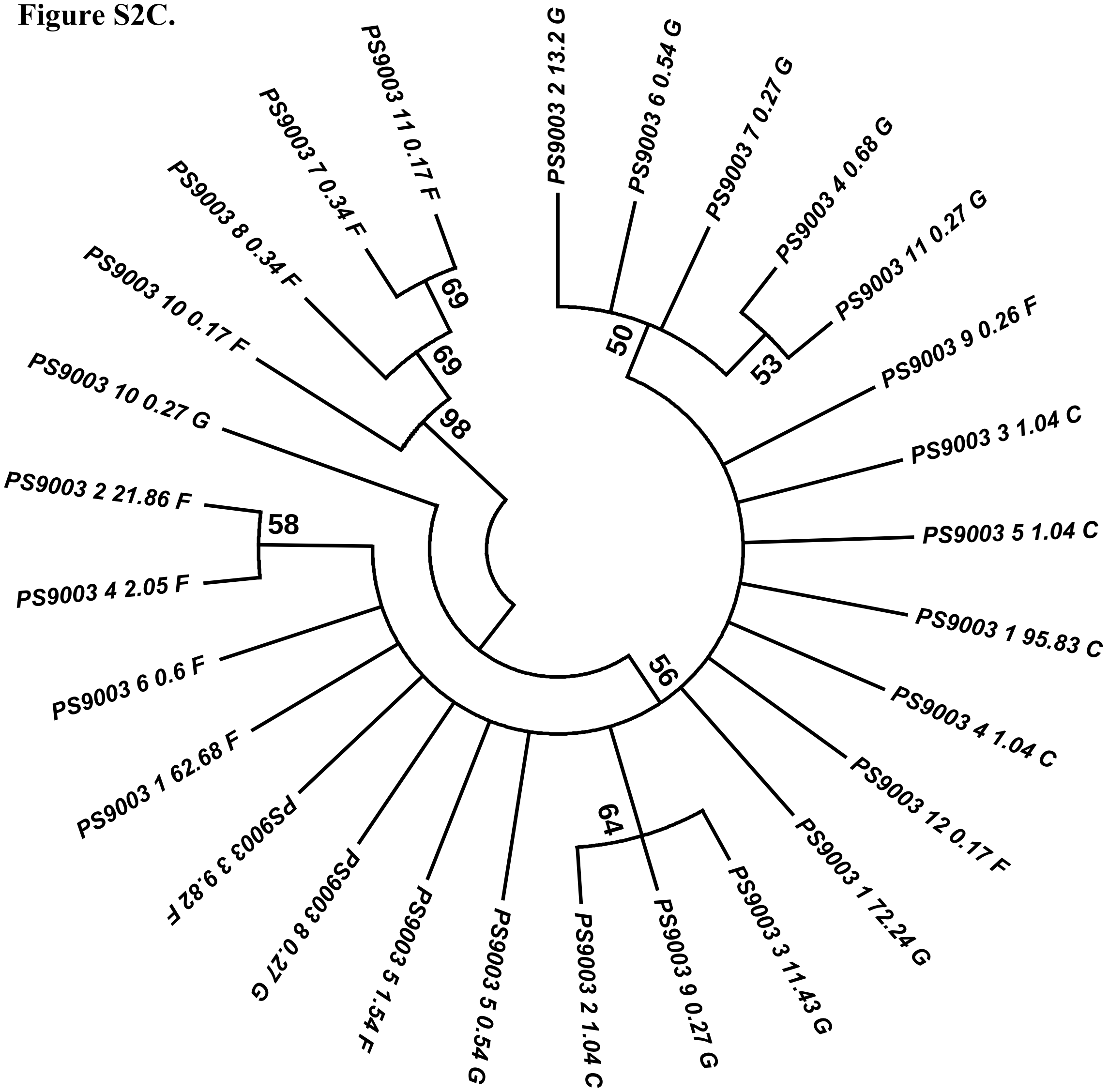
PS9003 2 13.2 G
PS9003 6 0.54 G
PS9003 7 0.27 G
PS9003 4 0.68 G
PS9003 11 0.27 G
PS9003 9 0.26 F
PS9003 3 1.04 C
PS9003 5 1.04 C
PS9003 1 95.83 C
PS9003 4 1.04 C
PS9003 12 0.17 F
PS9003 1 72.24 G
PS9003 3 11.43 G
PS9003 9 0.27 G
PS9003 2 1.04 C
PS9003 5 0.54 G
PS9003 5 1.54 F
PS9003 8 0.27 G
PS9003 3 9.82 F
PS9003 1 62.68 F
PS9003 6 0.6 F
PS9003 4 2.05 F
PS9003 2 21.86 F
PS9003 10 0.27 G
PS9003 10 0.17 F
PS9003 8 0.34 F
PS9003 7 0.34 F
PS9003 11 0.17 F
50
53
56
64
58
98
69
69

Figure S2D.

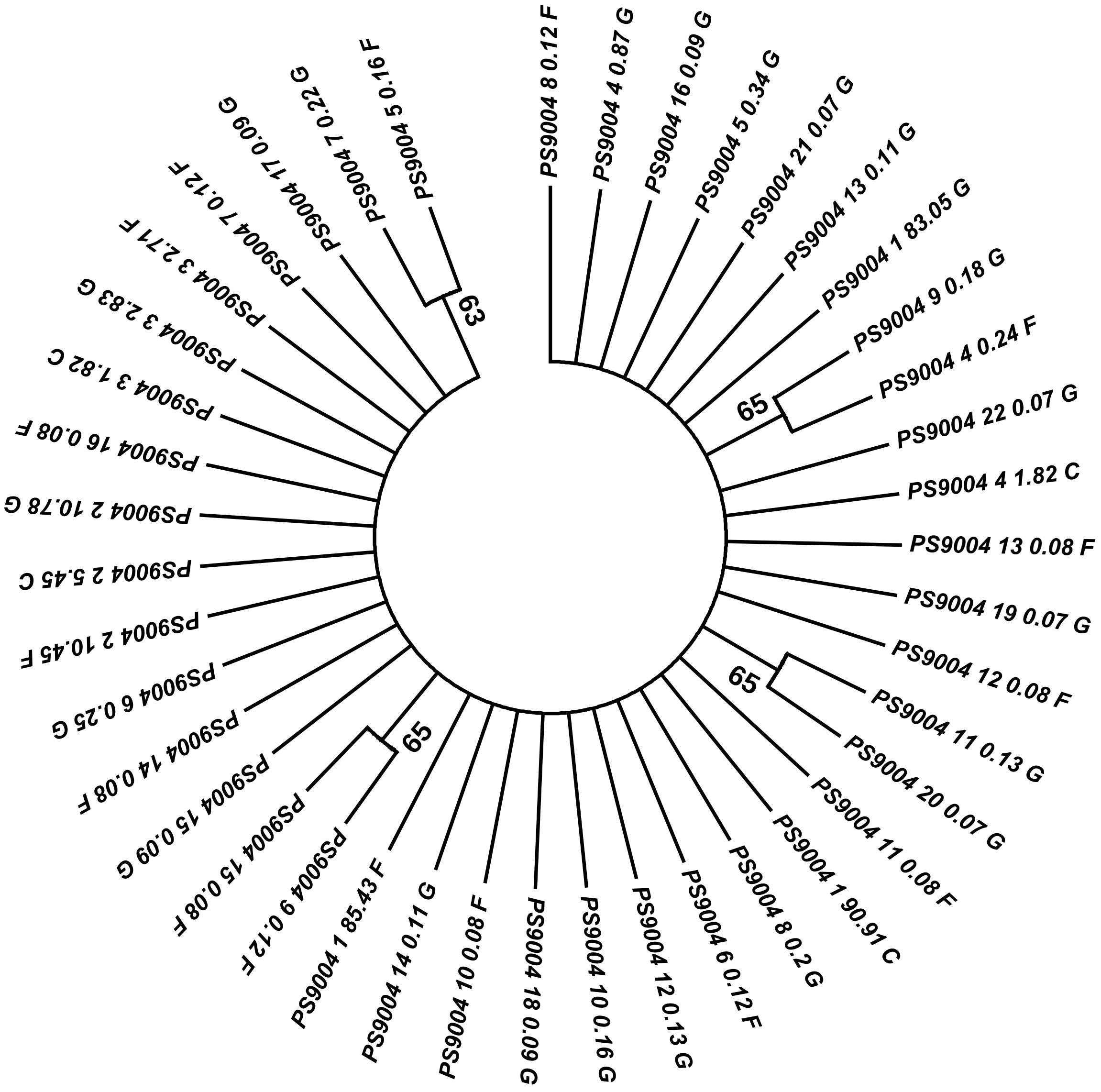

PS9004 8 0.12 F
PS9004 4 0.87 G
PS9004 16 0.09 G
PS9004 5 0.34 G
PS9004 21 0.07 G
PS9004 13 0.11 G
PS9004 1 83.05 G
PS9004 9 0.18 G
PS9004 4 0.24 F
65
PS9004 22 0.07 G
PS9004 4 1.82 C
PS9004 13 0.08 F
PS9004 19 0.07 G
PS9004 12 0.08 F
PS9004 11 0.13 G
65
PS9004 20 0.07 G
PS9004 11 0.08 F
PS9004 1 90.91 C
PS9004 8 0.2 G
PS9004 6 0.12 F
PS9004 12 0.13 G
PS9004 10 0.16 G
PS9004 18 0.09 G
PS9004 10 0.08 F
PS9004 14 0.11 G
PS9004 1 85.43 F
PS9004 9 0.12 F
PS9004 15 0.08 F
65
PS9004 15 0.09 G
PS9004 14 0.08 F
PS9004 6 0.25 G
PS9004 2 10.45 F
PS9004 2 5.45 C
PS9004 2 10.78 G
PS9004 16 0.08 F
PS9004 3 1.82 C
PS9004 3 2.83 G
PS9004 3 2.71 F
PS9004 7 0.12 F
PS9004 17 0.09 G
PS9004 7 0.22 G
PS9004 5 0.16 F
63

Figure S2E.

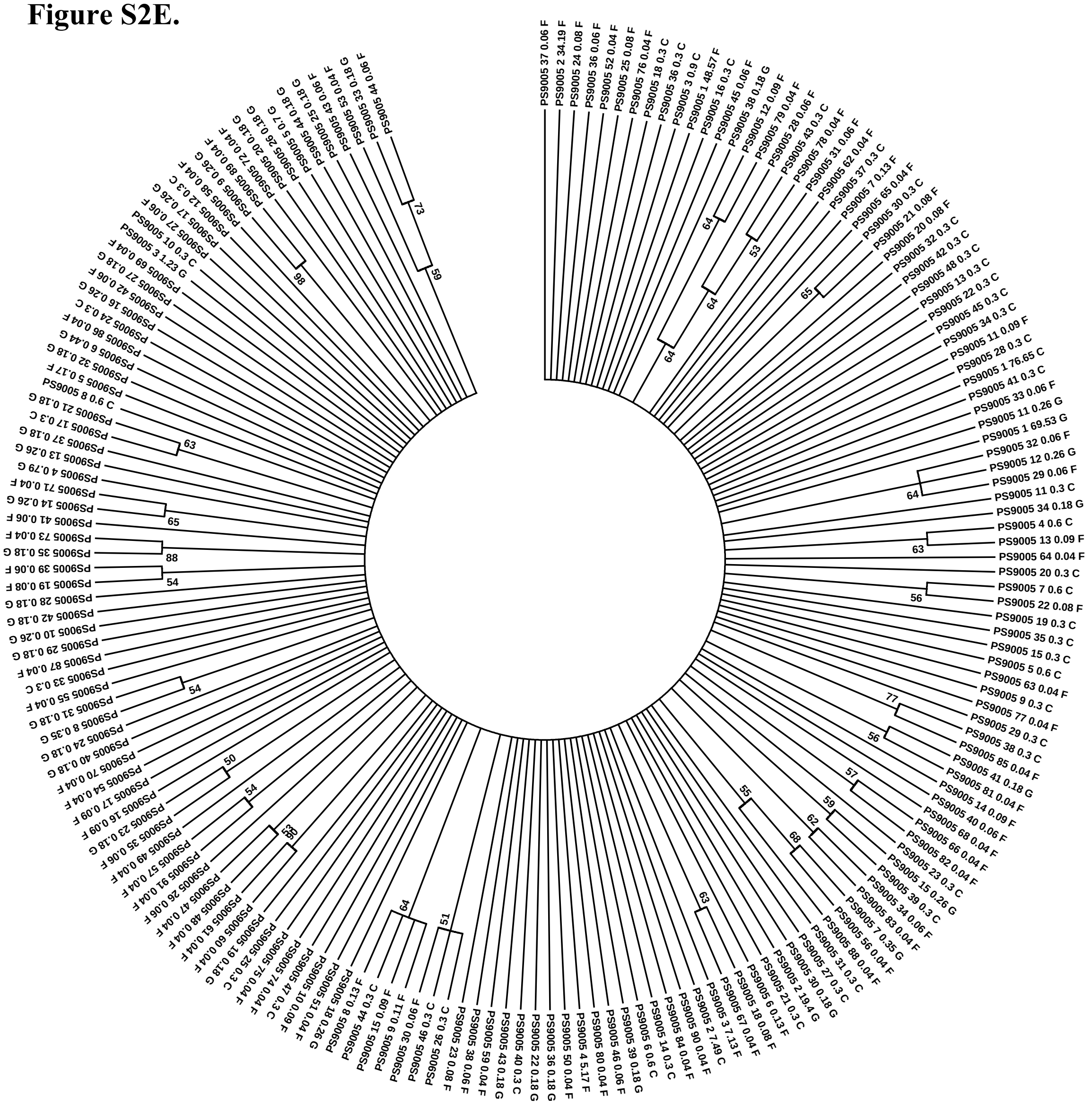

Supplement: Figure S2 — Comparison of ITS2 variants from completed genome sequence data, cloning data and 454 pyrosequencing data. For each species, an unrooted Neighbor-Joining tree shows the diversity of ITS2 variants. Each branch represents an ITS2 variant, labeled with the sample number followed by the rank of the variant, RVA of the variant, and the letter (G, C or F). The letter G indicates a variant derived from completed genome sequence data. The letter C represents a variant derived from cloning data. The letter F represents a variant derived from 454 data. (A) Unrooted tree of ITS2 variants in Arabidopsis thaliana. (B) Unrooted tree of ITS2 variants in Oryza sativa ssp. japonica. (C) Unrooted tree of ITS2 variants in Oryza sativa ssp. indica. (D) Unrooted tree of ITS2 variants in Populus trichocarpa. (E) Unrooted tree of ITS2 variants in Zea mays. (PDF) [file pone.0043971.s002.pdf]
