# Supplementary material for: Extensive Pyrosequencing Reveals Frequent Intra-Genomic Variations of Internal Transcribed Spacer Regions of Nuclear Ribosomal DNA
Source: PLoS One. 2012 Aug 30;7(8):e43971. doi: 10.1371/journal.pone.0043971 (PMC3431384; doi:10.1371/journal.pone.0043971)
Supplement: Figure S3 — Unrooted Maximum Parsimony trees displaying the evolutionary mechanisms of intra-genomic ITS2 variants in plant genomes. (A) The concerted evolution mechanism. Panax ginseng is used as an example. The red line indicates the most major variant. (B) The birth-and-death evolution mechanism. Arabidopsis thaliana is used as an example. The red lines indicate the two main variant clusters. (C) The divergent evolution mechanism. Solanum lyratum is used as an example. The red lines indicate multiple clusters of variants. (PDF) [file pone.0043971.s003.pdf]

Figure S3. Unrooted Maximum Parsimony trees displaying the evolutionary mechanisms of intra-genomic ITS2 variants in plant genomes. **(A)** The concerted evolution mechanism. *Panax ginseng* is used as an example. The red line indicates the most major variant. **(B)** The birth-and-death evolution mechanism. *Arabidopsis thaliana* is used as an example. The red lines indicate the two main variant clusters. **(C)** The divergent evolution mechanism. *Solanum lyratum* is used as an example. The red lines indicate multiple clusters of variants.

Figure S3A.

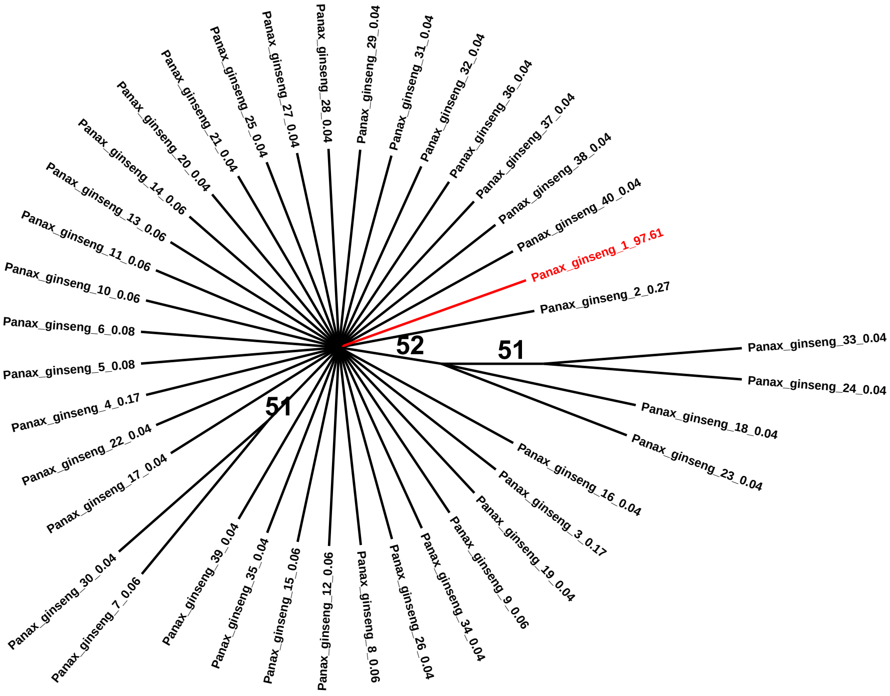

Figure S3B.

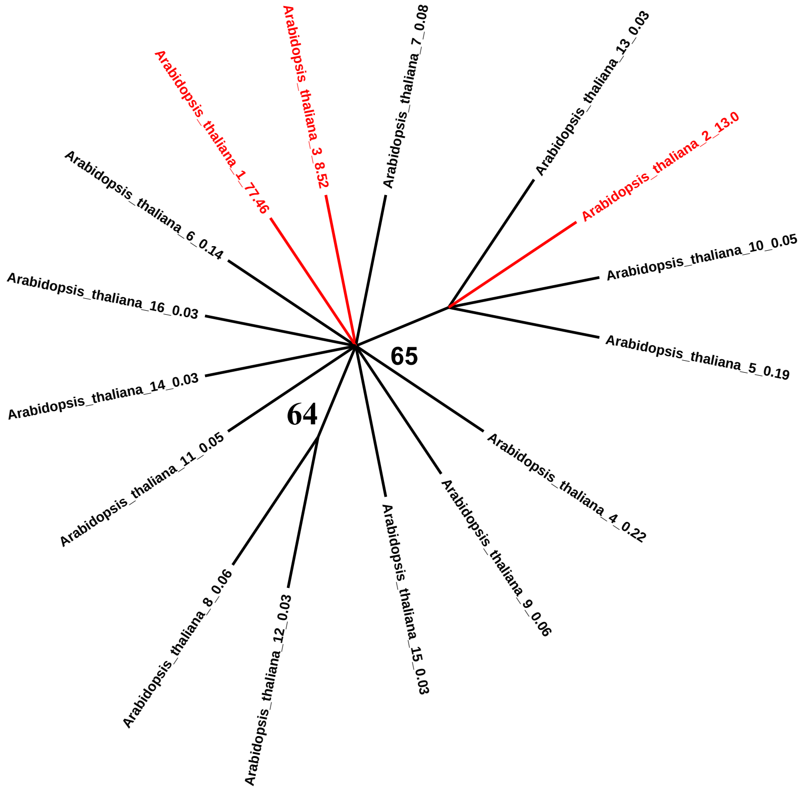

Figure S3C.

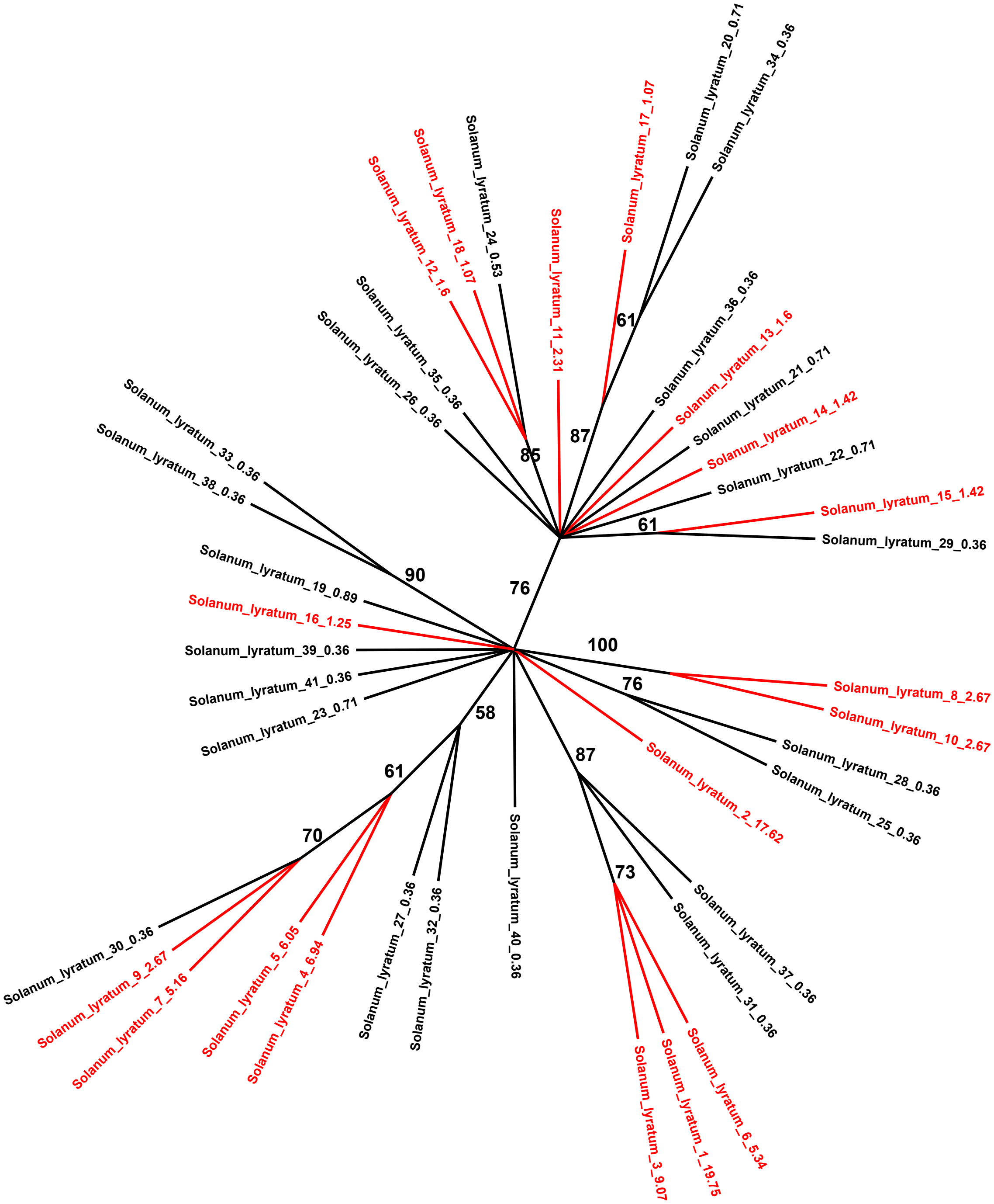
Solanum_lyratum_20_0.71
Solanum_lyratum_34_0.36
Solanum_lyratum_17_1.07
Solanum_lyratum_24_0.53
Solanum_lyratum_18_1.07
Solanum_lyratum_12_1.6
Solanum_lyratum_11_2.31
Solanum_lyratum_35_0.36
Solanum_lyratum_26_0.36
Solanum_lyratum_36_0.36
Solanum_lyratum_13_1.6
Solanum_lyratum_21_0.71
Solanum_lyratum_14_1.42
Solanum_lyratum_22_0.71
Solanum_lyratum_15_1.42
Solanum_lyratum_29_0.36
Solanum_lyratum_33_0.36
Solanum_lyratum_38_0.36
Solanum_lyratum_19_0.89
Solanum_lyratum_16_1.25
Solanum_lyratum_39_0.36
Solanum_lyratum_41_0.36
Solanum_lyratum_23_0.71
Solanum_lyratum_8_2.67
Solanum_lyratum_10_2.67
Solanum_lyratum_28_0.36
Solanum_lyratum_25_0.36
Solanum_lyratum_2_17.62
Solanum_lyratum_40_0.36
Solanum_lyratum_27_0.36
Solanum_lyratum_32_0.36
Solanum_lyratum_30_0.36
Solanum_lyratum_9_2.67
Solanum_lyratum_7_5.16
Solanum_lyratum_5_6.05
Solanum_lyratum_4_6.94
Solanum_lyratum_37_0.36
Solanum_lyratum_31_0.36
Solanum_lyratum_6_5.34
Solanum_lyratum_1_19.75
Solanum_lyratum_3_9.07
61
87
85
61
90
76
100
76
58
61
70
87
73
