# Supplementary figures and images for: Extensive Pyrosequencing Reveals Frequent Intra-Genomic Variations of Internal Transcribed Spacer Regions of Nuclear Ribosomal DNA
Source: PLoS One. 2012 Aug 30;7(8):e43971. doi: 10.1371/journal.pone.0043971 (PMC3431384; doi:10.1371/journal.pone.0043971)

Figure S4B.

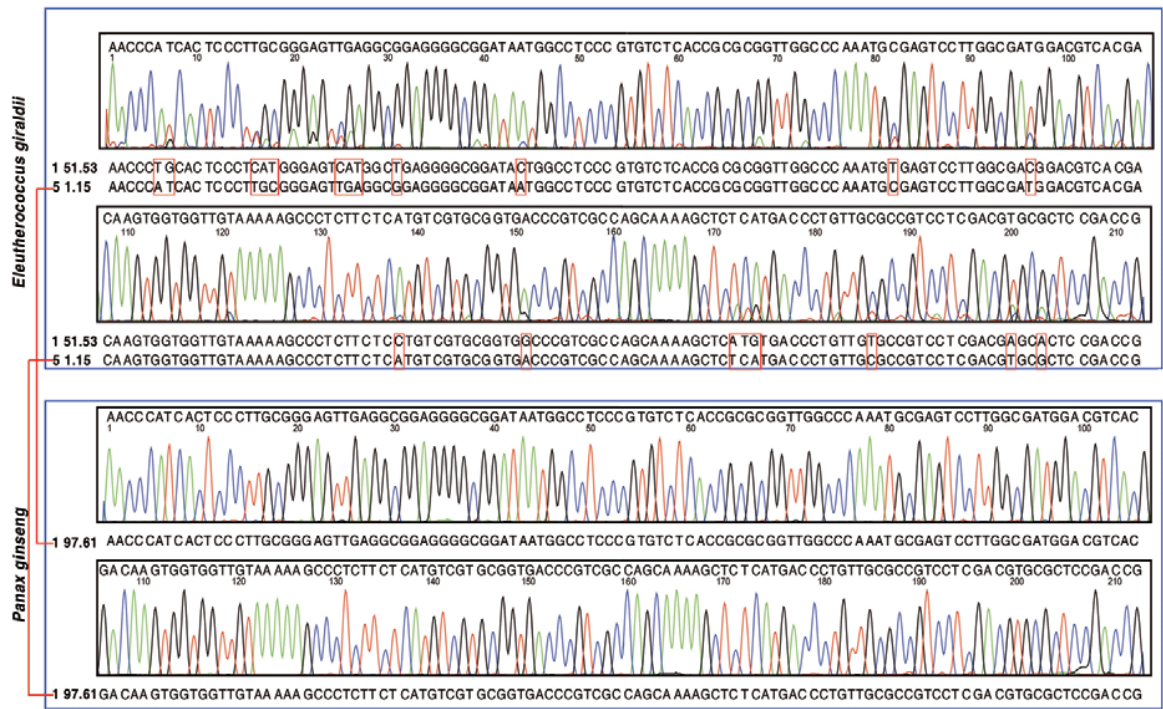

Supplement: Figure S4 — Two identical variants of ITS2 in the genera Panax and Eleutherococcus of Araliaceae. (A) The Maximum Parsimony (MP) tree of ITS2 in Araliaceae. One minor variant of ITS2 in Eleutherococcus giraldii showed close affinity to the ITS2 variants of Panax ginseng and was clustered with them. (B) This ITS2 variant, which was the major variant in the Panax ginseng genome but minor in Eleutherococcus giraldii, was confirmed by direct sequencing of PCR products using specific primers. The sequencing trace from E. giraldii shows double peaks at some bases, and these double peaks correspond to the major ITS2 variant in P. ginseng. The bases having double peaks are boxed in red. (PDF) [file pone.0043971.s004.pdf]

## Hk wt g'U; A.

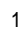

**Hk wt g'U; B.**

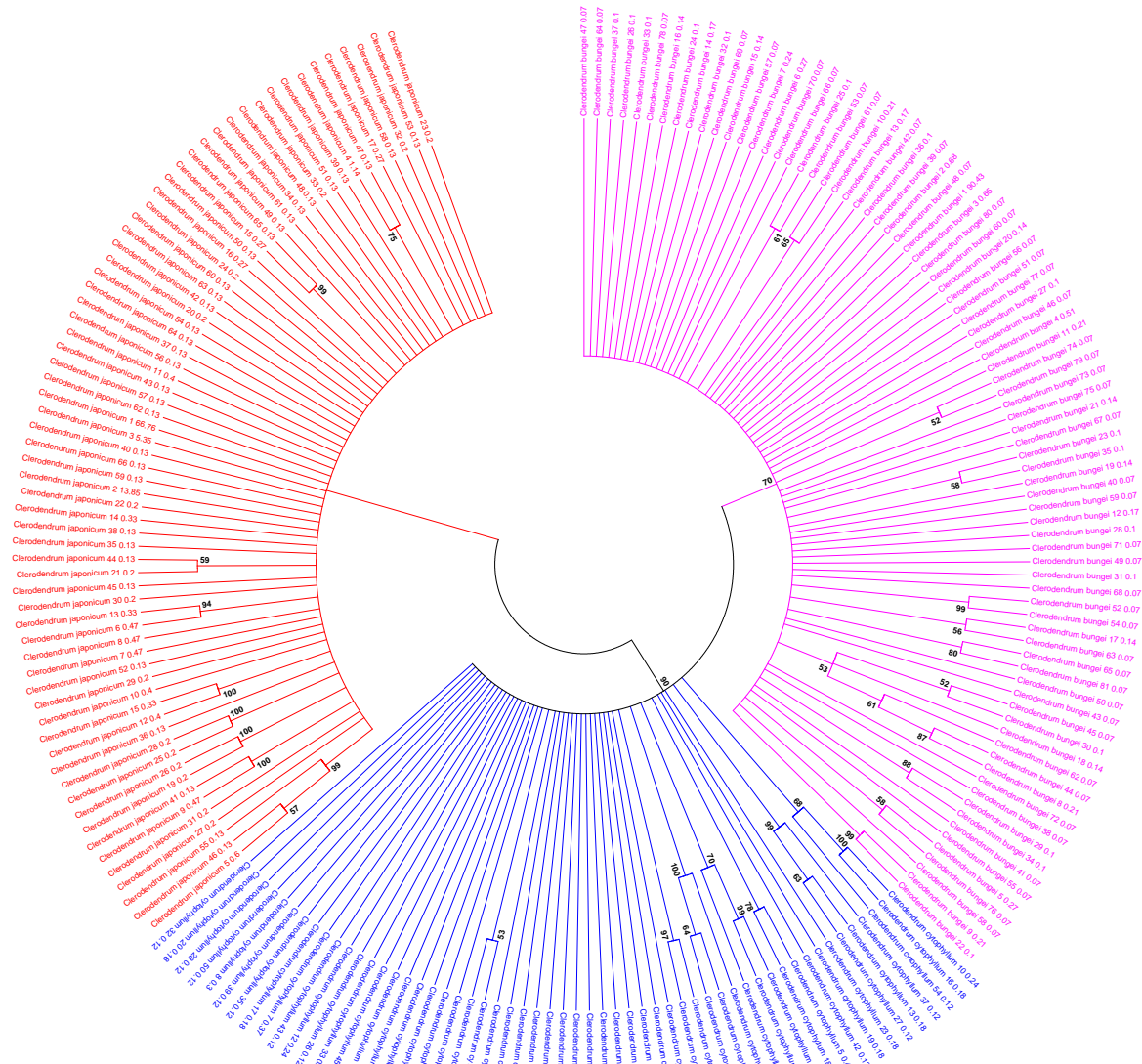

Figure S9C.

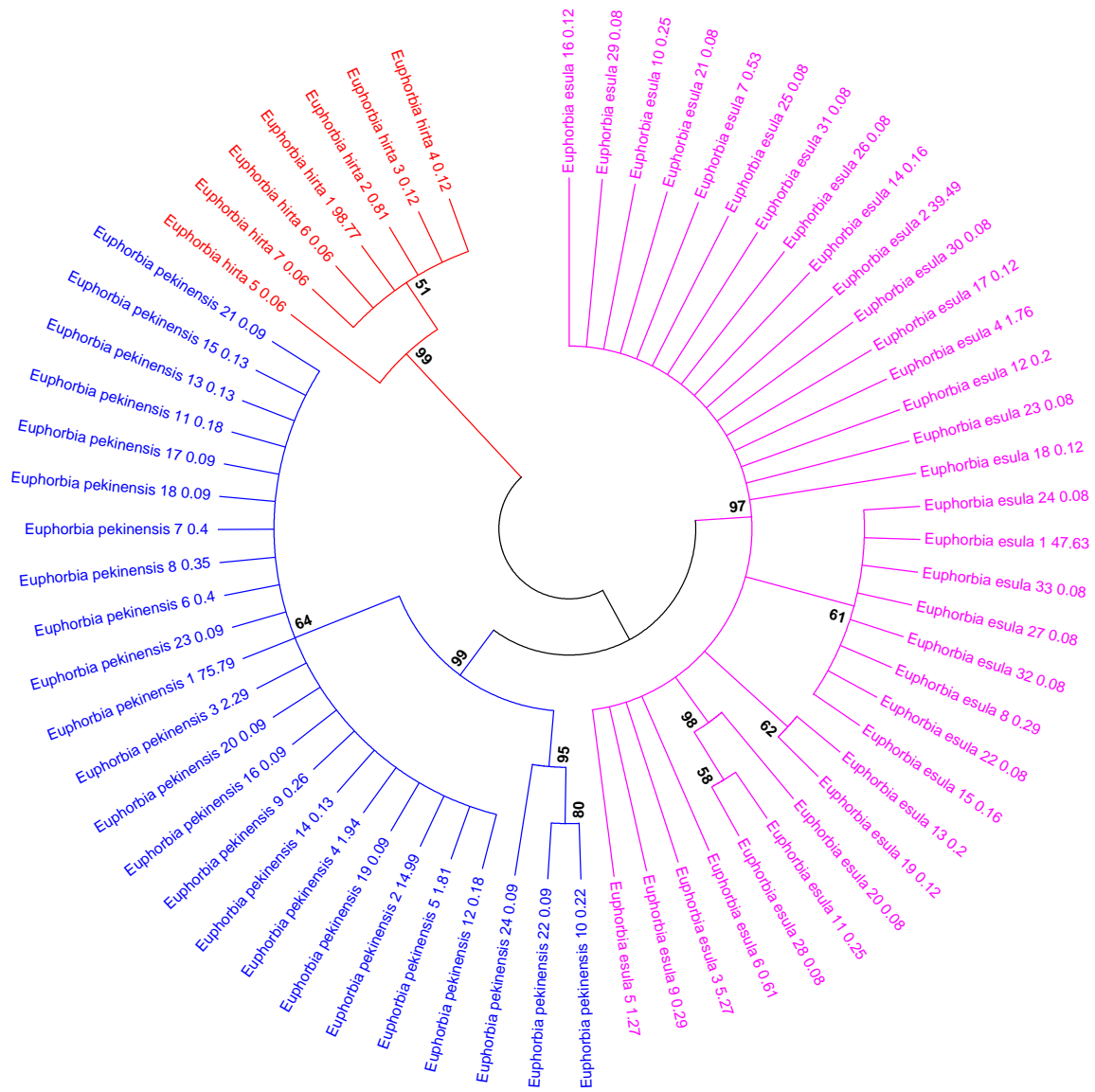

Supplement: Figure S9 — The Neighbor-Joining tree of ITS2 variants in the genera. (A) Astragalus (Fabaceae), (B) Clerodendrum (Verbenaceae), and (C) Euphorbia (Euphorbiaceae). The same colors represent the same species. The Latin names of species are followed by the rank and RVA of the variants. (PDF) [file pone.0043971.s009.pdf]

**Figure S10.** The work flow for the processing and analysis of pyrosequencing reads.

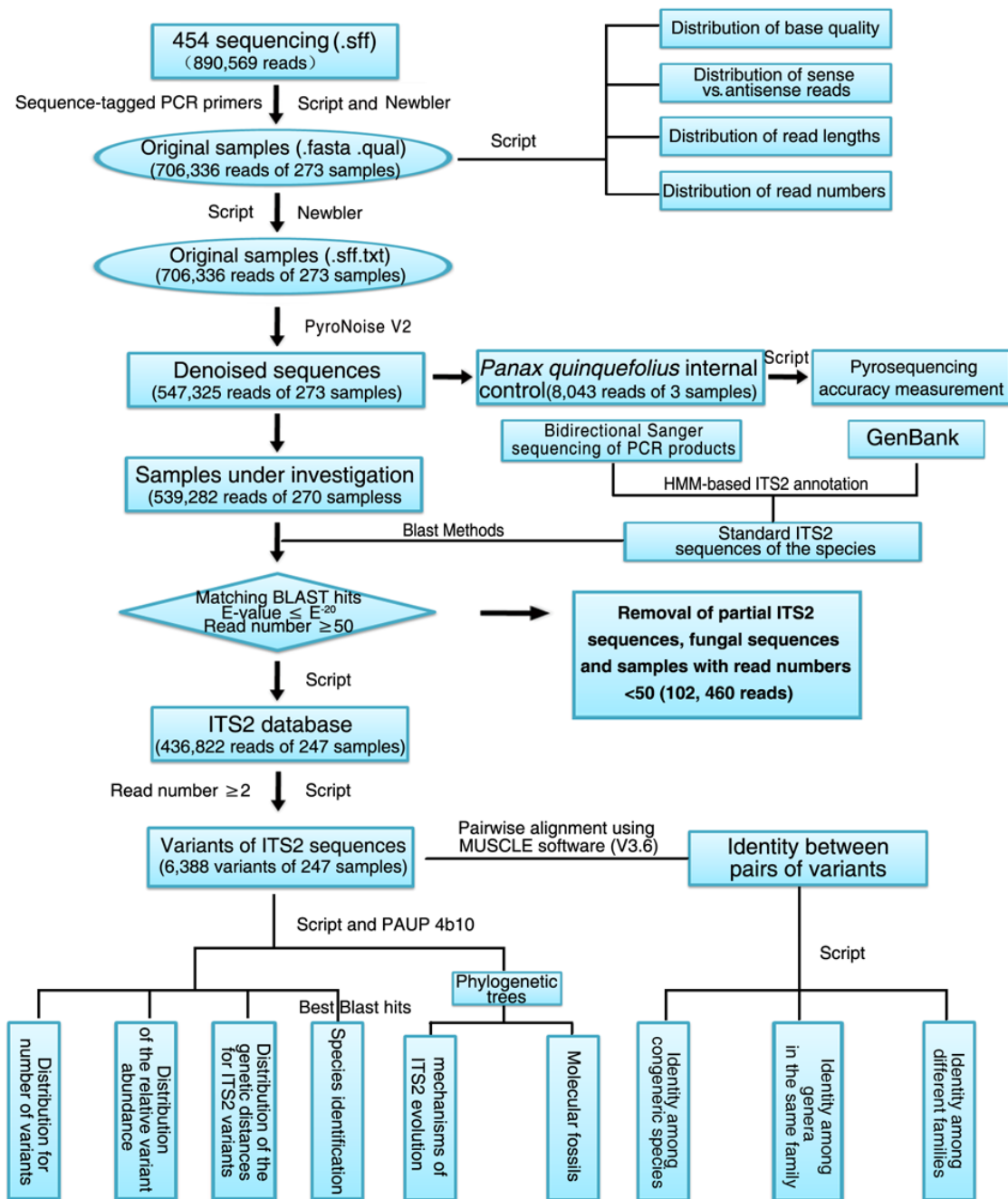

Supplement: Figure S10 — The work flow for the processing and analysis of pyrosequencing reads. (PDF) [file pone.0043971.s010.pdf]
