# Supplementary material for: Extensive Pyrosequencing Reveals Frequent Intra-Genomic Variations of Internal Transcribed Spacer Regions of Nuclear Ribosomal DNA
Source: PLoS One. 2012 Aug 30;7(8):e43971. doi: 10.1371/journal.pone.0043971 (PMC3431384; doi:10.1371/journal.pone.0043971)
Supplement: Figure S5 — ITS2 variants representing molecular fossils in the genus Armeniaca (Rosaceae). (A) The variants are linked using red and blue lines at 100% and 95% identities, respectively. Each open box represents a single variant with RVA over 1%, and each solid box represents the variants with RVA less than 1%. The ruler shows the RVA in percentage. (B,C) The Neighbor-Joining (NJ) and Maximum Parsimony (MP) trees of ITS2 variants indicated that genetic information from the common ancestor was maintained in the genomes of two species derived from Armeniaca (Rosaceae). The same colors represent the same species. The same shade and color of symbols show the variants with 100% identity. The Latin names of species are followed by the rank and RVA of the variants. (D) The ITS2 variants in A. vulgaris were confirmed by direct sequencing of PCR products. The sequencing trace from A. vulgaris shows double peaks at some bases, and these double peaks correspond to the different ITS2 variants in A. vulgaris. The bases with double peaks are boxed in red. (E-H) The variants as molecular fossils of A. sibirica were confirmed by cloning of PCR products. (PDF) [file pone.0043971.s005.pdf]

Figure S5. ITS2 variants representing molecular fossils in the genus *Armeniaca* (Rosaceae) **(A)** The variants are linked using red and blue lines at 100% and 95% identities, respectively. Each open box represents a single variant with RVA over 1%, and each solid box represents the variants with RVA less than 1%. The ruler shows the RVA in percentage. **(B,C)** The Neighbor-Joining (NJ) and Maximum Parsimony (MP) trees of ITS2 variants indicated that genetic information from the common ancestor was maintained in the genomes of two species derived from *Armeniaca* (Rosaceae). The same colors represent the same species. The same shade and color of symbols show the variants with 100% identity. The Latin names of species are followed by the rank and RVA of the variants. **(D)** The ITS2 variants in *A. vulgaris* were confirmed by direct sequencing of PCR products. The sequencing trace from *A. vulgaris* shows double peaks at some bases, and these double peaks correspond to the different ITS2 variants in *A. vulgaris*. The bases with double peaks are boxed in red. **(E-H)** The variants as molecular fossils of *A. sibirica* were confirmed by cloning of PCR products.

Figure S5A.

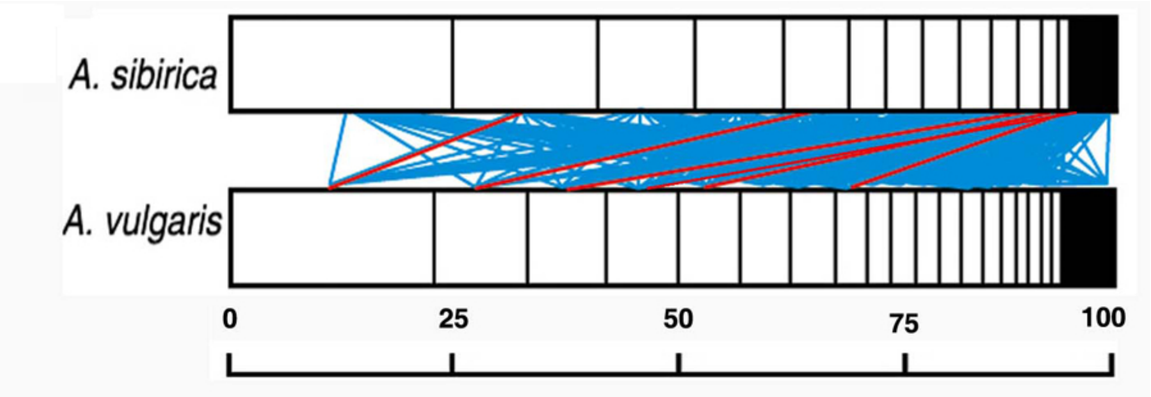

Figure S5B.

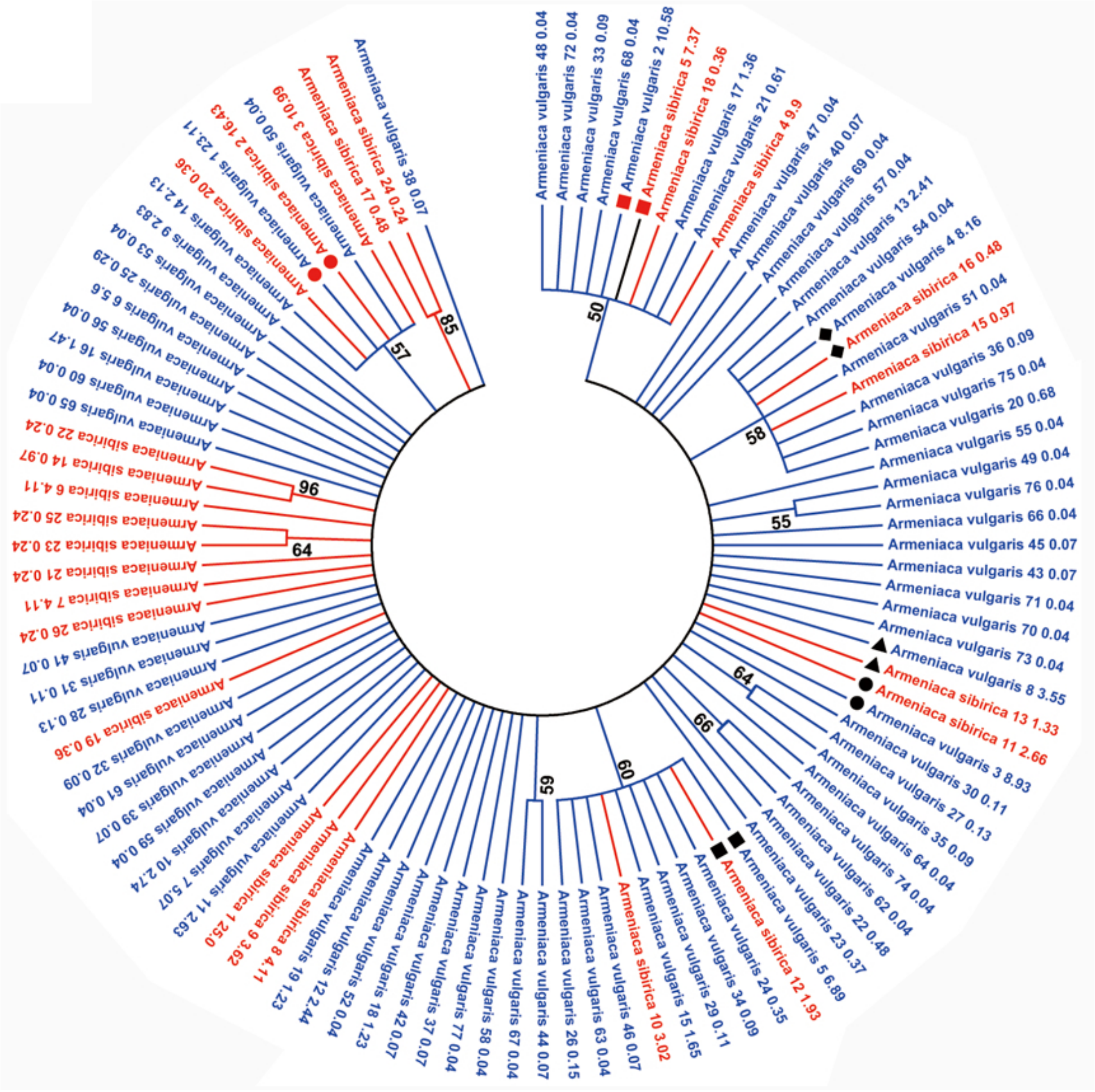
Armeniaca vulgaris 48 0.04
Armeniaca vulgaris 72 0.04
Armeniaca vulgaris 33 0.09
Armeniaca vulgaris 68 0.04
Armeniaca vulgaris 2 10.58
Armeniaca sibirica 5 7.37
Armeniaca sibirica 18 0.36
Armeniaca vulgaris 17 1.36
Armeniaca vulgaris 21 0.61
Armeniaca sibirica 4 9.9
Armeniaca vulgaris 47 0.04
Armeniaca vulgaris 40 0.07
Armeniaca vulgaris 69 0.04
Armeniaca vulgaris 57 0.04
Armeniaca vulgaris 13 2.41
Armeniaca vulgaris 54 0.04
Armeniaca vulgaris 4 8.16
Armeniaca sibirica 16 0.48
Armeniaca vulgaris 51 0.04
Armeniaca sibirica 15 0.97
Armeniaca vulgaris 36 0.09
Armeniaca vulgaris 75 0.04
Armeniaca vulgaris 20 0.68
Armeniaca vulgaris 55 0.04
Armeniaca vulgaris 49 0.04
Armeniaca vulgaris 76 0.04
Armeniaca vulgaris 66 0.04
Armeniaca vulgaris 45 0.07
Armeniaca vulgaris 43 0.07
Armeniaca vulgaris 71 0.04
Armeniaca vulgaris 70 0.04
Armeniaca vulgaris 73 0.04
Armeniaca vulgaris 8 3.55
Armeniaca sibirica 13 1.33
Armeniaca sibirica 11 2.66
Armeniaca vulgaris 3 8.93
Armeniaca vulgaris 30 0.11
Armeniaca vulgaris 27 0.13
Armeniaca vulgaris 35 0.09
Armeniaca vulgaris 64 0.04
Armeniaca vulgaris 74 0.04
Armeniaca vulgaris 62 0.04
Armeniaca vulgaris 22 0.48
Armeniaca vulgaris 23 0.37
Armeniaca vulgaris 5 6.89
Armeniaca sibirica 12 1.93
Armeniaca vulgaris 24 0.35
Armeniaca vulgaris 34 0.09
Armeniaca vulgaris 29 0.11
Armeniaca vulgaris 15 1.65
Armeniaca sibirica 10 3.02
Armeniaca vulgaris 46 0.07
Armeniaca vulgaris 63 0.04
Armeniaca vulgaris 26 0.15
Armeniaca vulgaris 44 0.07
Armeniaca vulgaris 67 0.04
Armeniaca vulgaris 58 0.04
Armeniaca vulgaris 77 0.04
Armeniaca vulgaris 37 0.07
Armeniaca vulgaris 42 0.07
Armeniaca vulgaris 18 1.23
Armeniaca vulgaris 52 0.04
Armeniaca vulgaris 12 2.44
Armeniaca vulgaris 19 1.23
Armeniaca sibirica 8 4.11
Armeniaca sibirica 9 3.62
Armeniaca sibirica 1 25.0
Armeniaca vulgaris 11 2.63
Armeniaca vulgaris 7 5.07
Armeniaca vulgaris 10 2.74
Armeniaca vulgaris 59 0.04
Armeniaca vulgaris 39 0.07
Armeniaca vulgaris 61 0.04
Armeniaca vulgaris 32 0.09
Armeniaca sibirica 19 0.36
Armeniaca vulgaris 28 0.13
Armeniaca vulgaris 31 0.11
Armeniaca vulgaris 41 0.07
Armeniaca sibirica 26 0.24
Armeniaca sibirica 7 4.11
Armeniaca sibirica 21 0.24
Armeniaca sibirica 23 0.24
Armeniaca sibirica 25 0.24
Armeniaca sibirica 6 4.11
Armeniaca sibirica 14 0.97
Armeniaca sibirica 22 0.24
Armeniaca vulgaris 65 0.04
Armeniaca vulgaris 60 0.04
Armeniaca vulgaris 16 1.47
Armeniaca vulgaris 56 0.04
Armeniaca vulgaris 6 5.6
Armeniaca vulgaris 25 0.29
Armeniaca vulgaris 53 0.04
Armeniaca vulgaris 9 2.83
Armeniaca vulgaris 14 2.13
Armeniaca sibirica 20 0.36
Armeniaca vulgaris 1 23.11
Armeniaca sibirica 2 16.43
Armeniaca vulgaris 50 0.04
Armeniaca sibirica 3 10.99
Armeniaca sibirica 17 0.48
Armeniaca sibirica 24 0.24
Armeniaca vulgaris 38 0.07
50
58
55
64
66
60
59
64
96
57
85

Figure S5C.

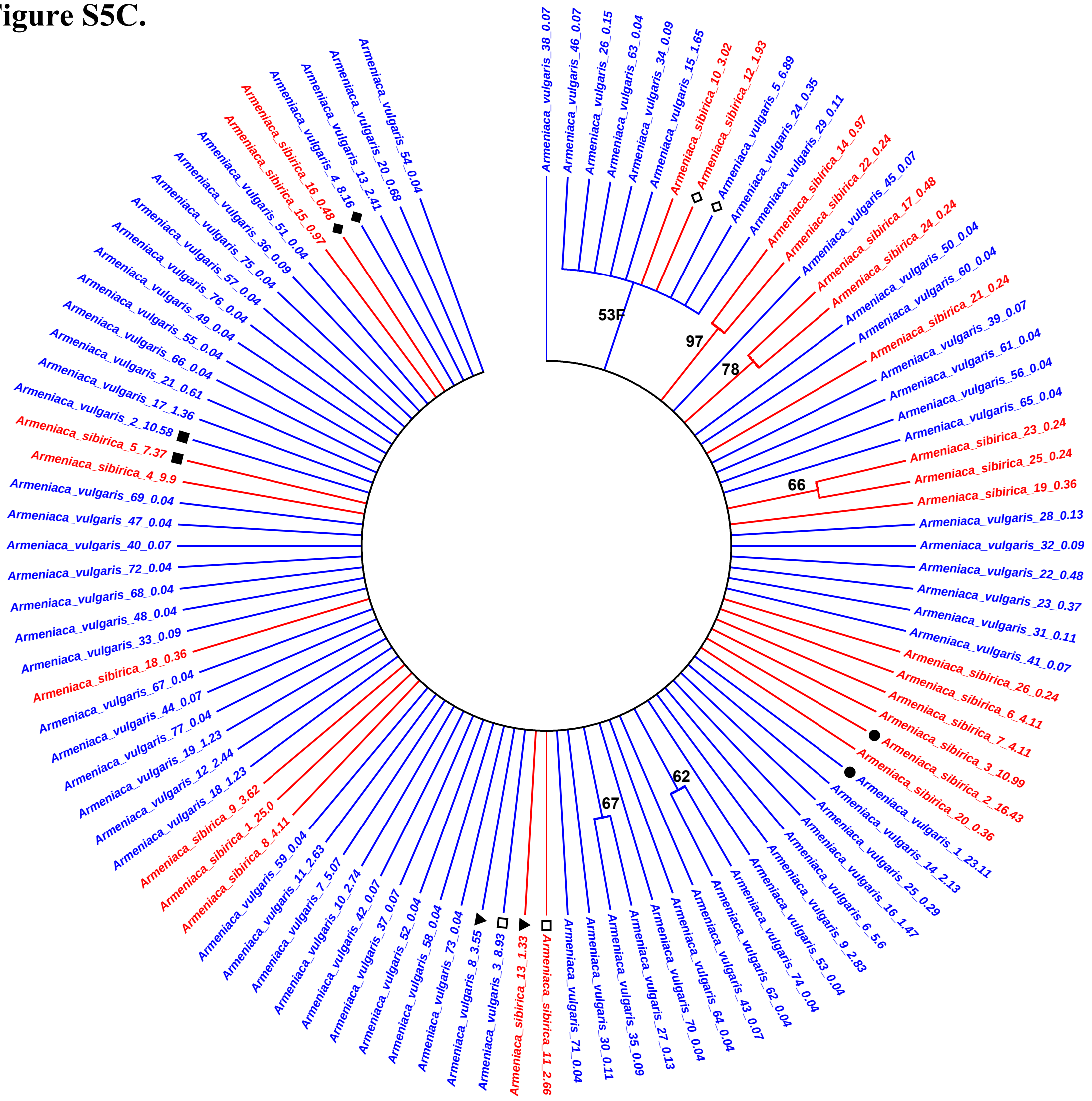

Armeniaca_vulgaris_38_0.07
Armeniaca_vulgaris_46_0.07
Armeniaca_vulgaris_26_0.15
Armeniaca_vulgaris_63_0.04
Armeniaca_vulgaris_34_0.09
Armeniaca_vulgaris_15_1.65
Armeniaca_sibirica_10_3.02
Armeniaca_sibirica_12_1.93
Armeniaca_vulgaris_5_6.89
Armeniaca_vulgaris_24_0.35
Armeniaca_vulgaris_29_0.11
Armeniaca_sibirica_14_0.97
Armeniaca_sibirica_22_0.24
Armeniaca_vulgaris_45_0.07
Armeniaca_sibirica_17_0.48
Armeniaca_sibirica_24_0.24
Armeniaca_vulgaris_50_0.04
Armeniaca_vulgaris_60_0.04
Armeniaca_sibirica_21_0.24
Armeniaca_vulgaris_39_0.07
Armeniaca_vulgaris_61_0.04
Armeniaca_vulgaris_56_0.04
Armeniaca_vulgaris_65_0.04
Armeniaca_sibirica_23_0.24
Armeniaca_sibirica_25_0.24
Armeniaca_sibirica_19_0.36
Armeniaca_vulgaris_28_0.13
Armeniaca_vulgaris_32_0.09
Armeniaca_vulgaris_22_0.48
Armeniaca_vulgaris_23_0.37
Armeniaca_vulgaris_31_0.11
Armeniaca_vulgaris_41_0.07
Armeniaca_sibirica_26_0.24
Armeniaca_sibirica_6_4.11
Armeniaca_sibirica_7_4.11
Armeniaca_sibirica_3_10.99
Armeniaca_sibirica_2_16.43
Armeniaca_sibirica_20_0.36
Armeniaca_vulgaris_1_23.11
Armeniaca_vulgaris_14_2.13
Armeniaca_vulgaris_25_0.29
Armeniaca_vulgaris_16_1.47
Armeniaca_vulgaris_6_5.56
Armeniaca_vulgaris_9_2.83
Armeniaca_vulgaris_53_0.04
Armeniaca_vulgaris_74_0.04
Armeniaca_vulgaris_62_0.04
Armeniaca_vulgaris_43_0.07
Armeniaca_vulgaris_64_0.04
Armeniaca_vulgaris_70_0.04
Armeniaca_vulgaris_27_0.13
Armeniaca_vulgaris_35_0.09
Armeniaca_vulgaris_30_0.11
Armeniaca_vulgaris_71_0.04
Armeniaca_sibirica_11_2.66
Armeniaca_sibirica_13_1.33
Armeniaca_vulgaris_3_8.93
Armeniaca_vulgaris_8_3.55
Armeniaca_vulgaris_73_0.04
Armeniaca_vulgaris_58_0.04
Armeniaca_vulgaris_52_0.04
Armeniaca_vulgaris_37_0.07
Armeniaca_vulgaris_42_0.07
Armeniaca_vulgaris_10_2.74
Armeniaca_vulgaris_7_5.07
Armeniaca_vulgaris_11_2.63
Armeniaca_vulgaris_59_0.04
Armeniaca_sibirica_8_4.11
Armeniaca_sibirica_1_25.0
Armeniaca_sibirica_9_3.62
Armeniaca_vulgaris_18_1.23
Armeniaca_vulgaris_12_2.44
Armeniaca_vulgaris_19_1.23
Armeniaca_vulgaris_77_0.04
Armeniaca_vulgaris_44_0.07
Armeniaca_vulgaris_67_0.04
Armeniaca_sibirica_18_0.36
Armeniaca_vulgaris_33_0.09
Armeniaca_vulgaris_48_0.04
Armeniaca_vulgaris_68_0.04
Armeniaca_vulgaris_72_0.04
Armeniaca_vulgaris_40_0.07
Armeniaca_vulgaris_47_0.04
Armeniaca_vulgaris_69_0.04
Armeniaca_sibirica_4_9.9
Armeniaca_sibirica_5_7.37
Armeniaca_vulgaris_2_10.58
Armeniaca_vulgaris_17_1.36
Armeniaca_vulgaris_21_0.61
Armeniaca_vulgaris_66_0.04
Armeniaca_vulgaris_55_0.04
Armeniaca_vulgaris_49_0.04
Armeniaca_vulgaris_76_0.04
Armeniaca_vulgaris_57_0.04
Armeniaca_vulgaris_75_0.04
Armeniaca_vulgaris_36_0.09
Armeniaca_vulgaris_51_0.04
Armeniaca_sibirica_15_0.97
Armeniaca_sibirica_16_0.48
Armeniaca_vulgaris_4_8.16
Armeniaca_vulgaris_13_2.41
Armeniaca_vulgaris_20_0.68
Armeniaca_vulgaris_54_0.04
53
97
78
66
62
67

Figure S5D.

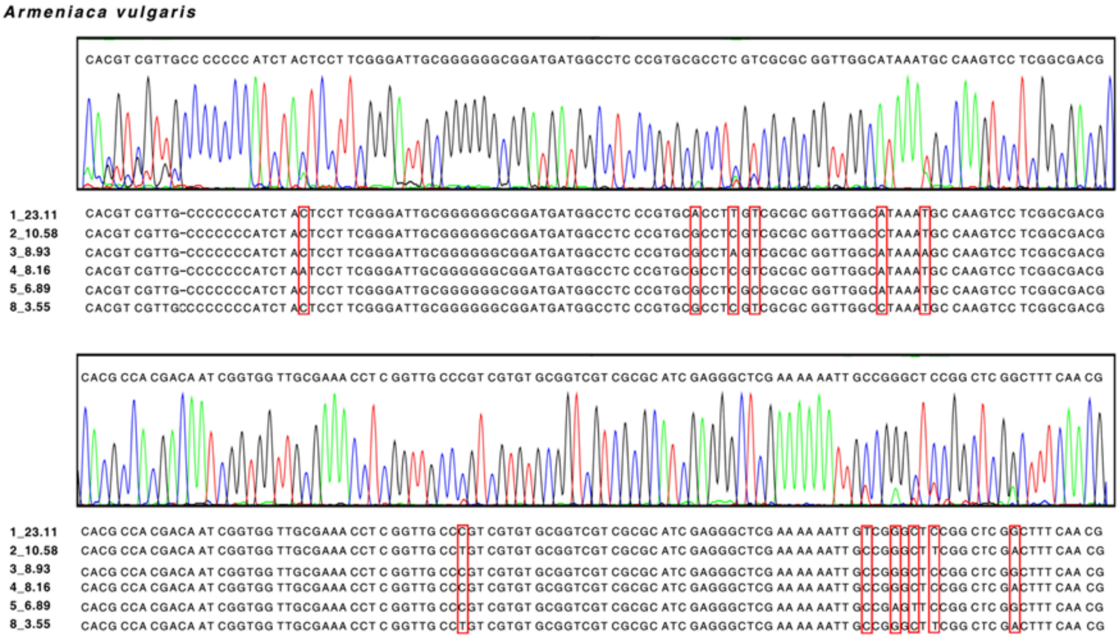

Figure S5E.

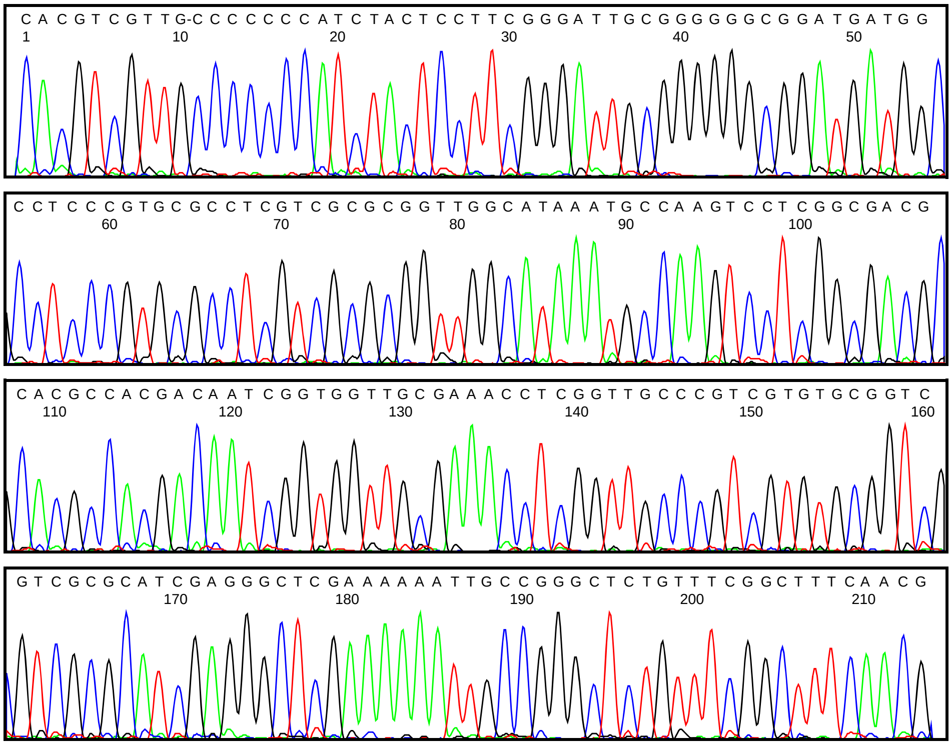

Figure S5F.

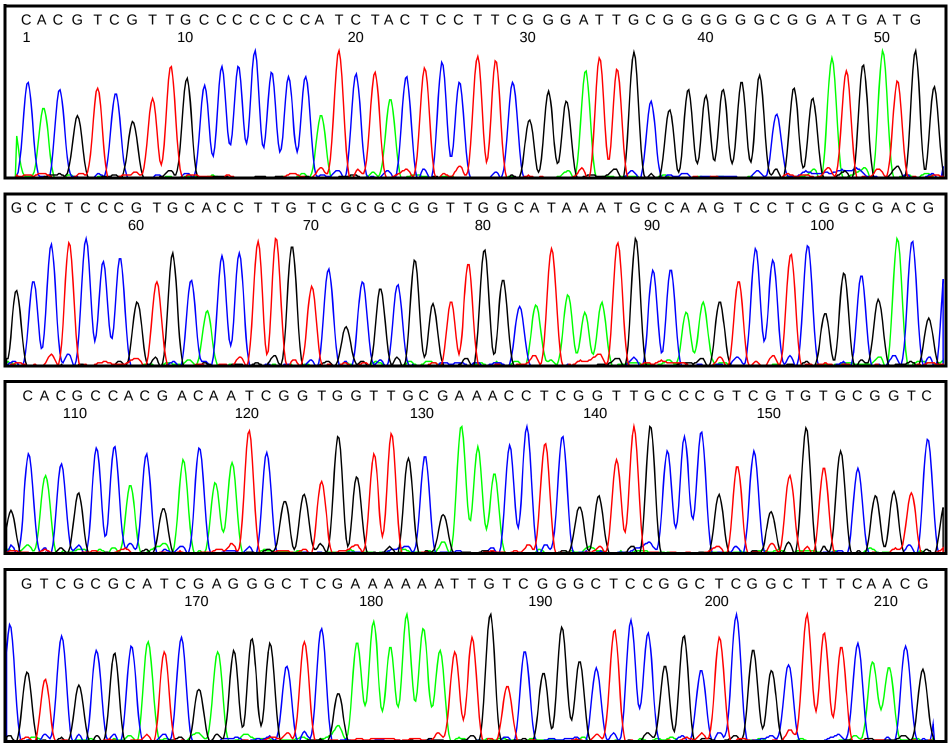

Figure S5G.

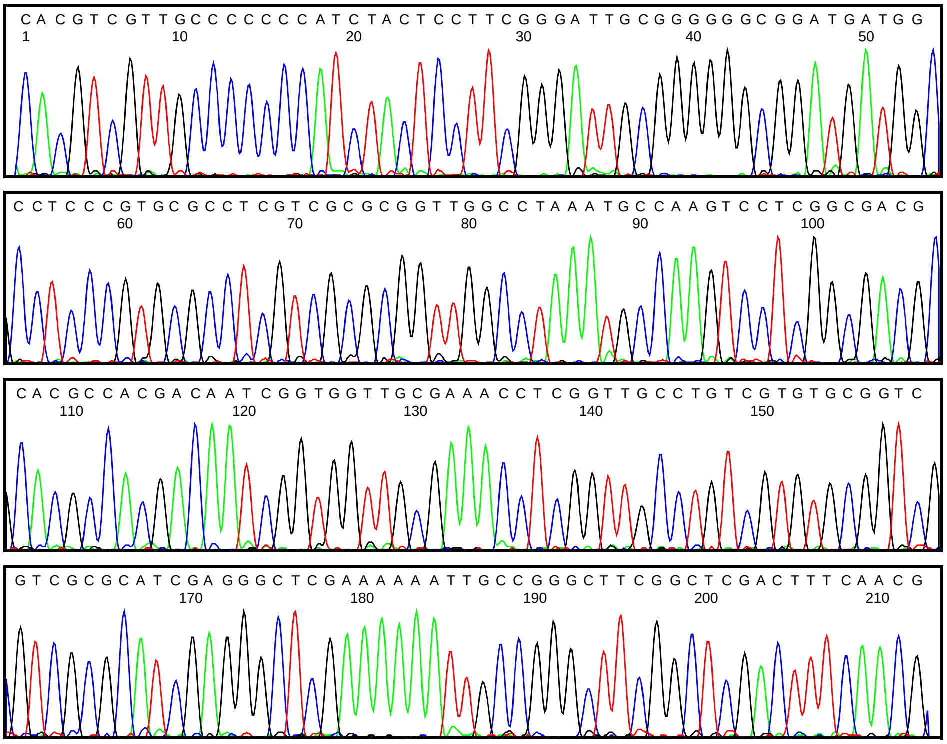

Figure S5H.

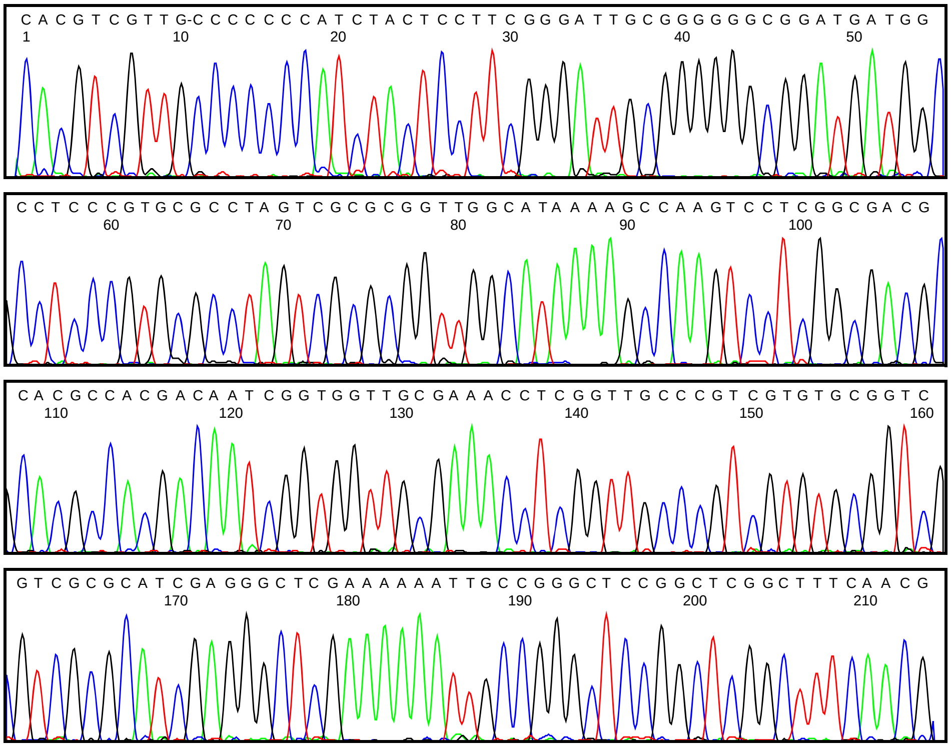
C A C G T C G T T G-C C C C C C C A T C T A C T C C T T C G G G A T T G C G G G G G G C G G A T G A T G G
1 10 20 30 40 50
C C T C C C G T G C G C C T A G T C G C G C G G T T G G C A T A A A A G C C A A G T C C T C G G C G A C G
60 70 80 90 100
C A C G C C A C G A C A A T C G G T G G T T G C G A A A C C T C G G T T G C C C G T C G T G T G C G G T C
110 120 130 140 150 160
G T C G C G C A T C G A G G G C T C G A A A A A A T T G C C G G G C T C C G G C T C G G C T T T C A A C G
170 180 190 200 210
