# Supplementary material for: Extensive Pyrosequencing Reveals Frequent Intra-Genomic Variations of Internal Transcribed Spacer Regions of Nuclear Ribosomal DNA
Source: PLoS One. 2012 Aug 30;7(8):e43971. doi: 10.1371/journal.pone.0043971 (PMC3431384; doi:10.1371/journal.pone.0043971)

Figure S6. The variants are linked using red and blue lines at 100% and 95% identities, respectively. Each open box represents a single variant with RVA over 1%, and each solid box represents the variants with RVA less than 1%. The rulers show the RVA in percentage. **(A)** *Epimedium* (Berberidaceae). **(B)** *Inula* (Asteraceae). **(C)** *Ipomoea* (Convolvulaceae). **(D)** *Panax* (Araliaceae). **(E)** *Pinus* (Pinaceae).

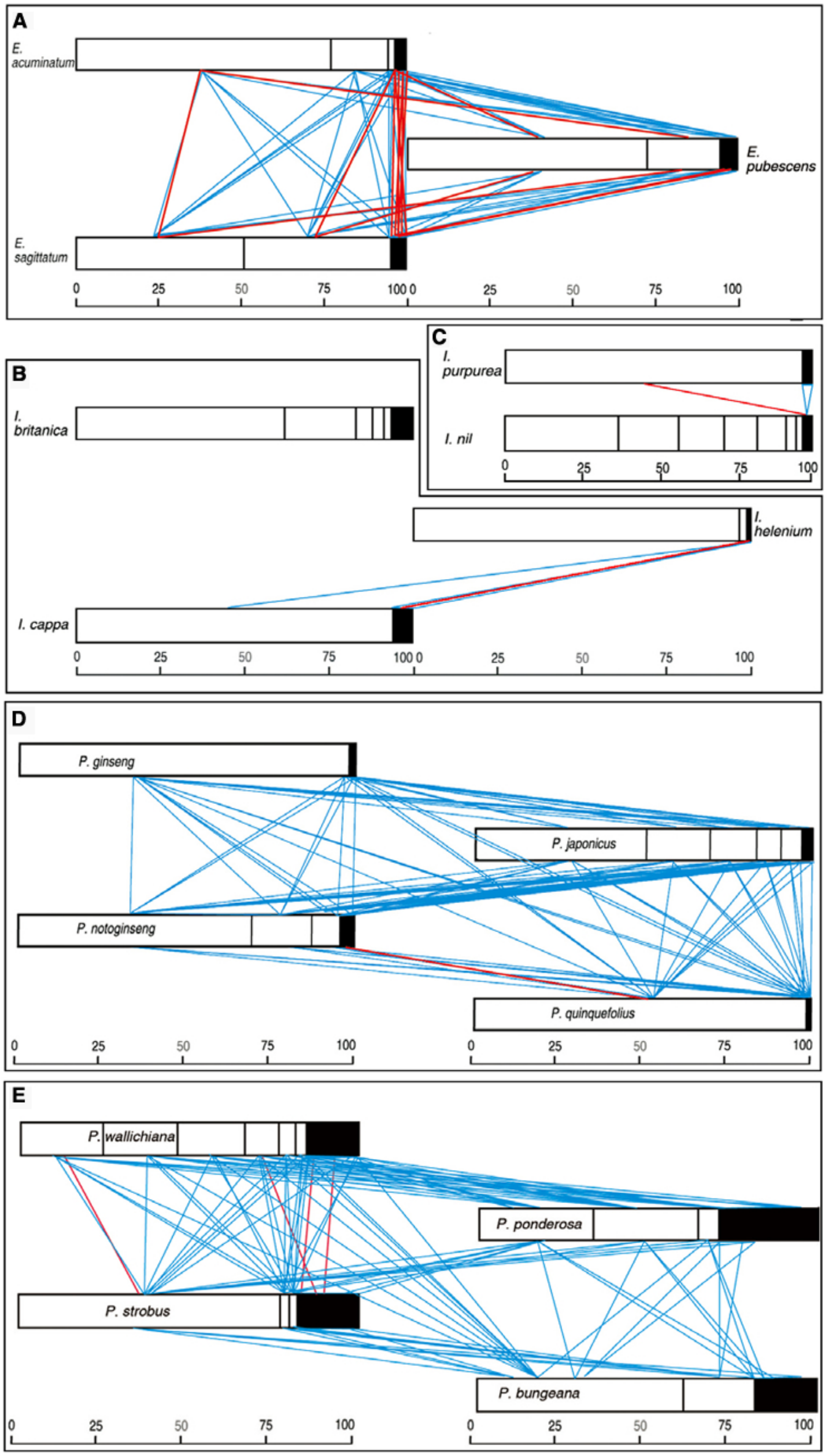

Supplement: Figure S6 — The variants are linked using red and blue lines at 100% and 95% identities, respectively. Each open box represents a single variant with RVA over 1%, and each solid box represents the variants with RVA less than 1%. The rulers show the RVA in percentage. (A) Epimedium (Berberidaceae). (B) Inula (Asteraceae). (C) Ipomoea (Convolvulaceae). (D) Panax (Araliaceae). (E) Pinus (Pinaceae). (PDF) [file pone.0043971.s006.pdf]
