# Supplementary material for: Extensive Pyrosequencing Reveals Frequent Intra-Genomic Variations of Internal Transcribed Spacer Regions of Nuclear Ribosomal DNA
Source: PLoS One. 2012 Aug 30;7(8):e43971. doi: 10.1371/journal.pone.0043971 (PMC3431384; doi:10.1371/journal.pone.0043971)

Figure S7. The Neighbor-Joining and the Maximum Parsimony trees of ITS2 variants indicated that genetic information from the common ancestor was maintained in the genomes of descendants. The same colors represent the same species. The same shade and color of symbols show the variants with 100% identity. The Latin names of species are followed by the rank and RVA of the variants. **(A,B)** *Epimedium* (Berberidaceae). **(C,D)** *Inula* (Asteraceae). **(E,F)** *Ipomoea* (Convolvulaceae). **(G,H)** *Panax* (Araliaceae). **(I,J)** *Pinus* (Pinaceae).

Figure S7A.

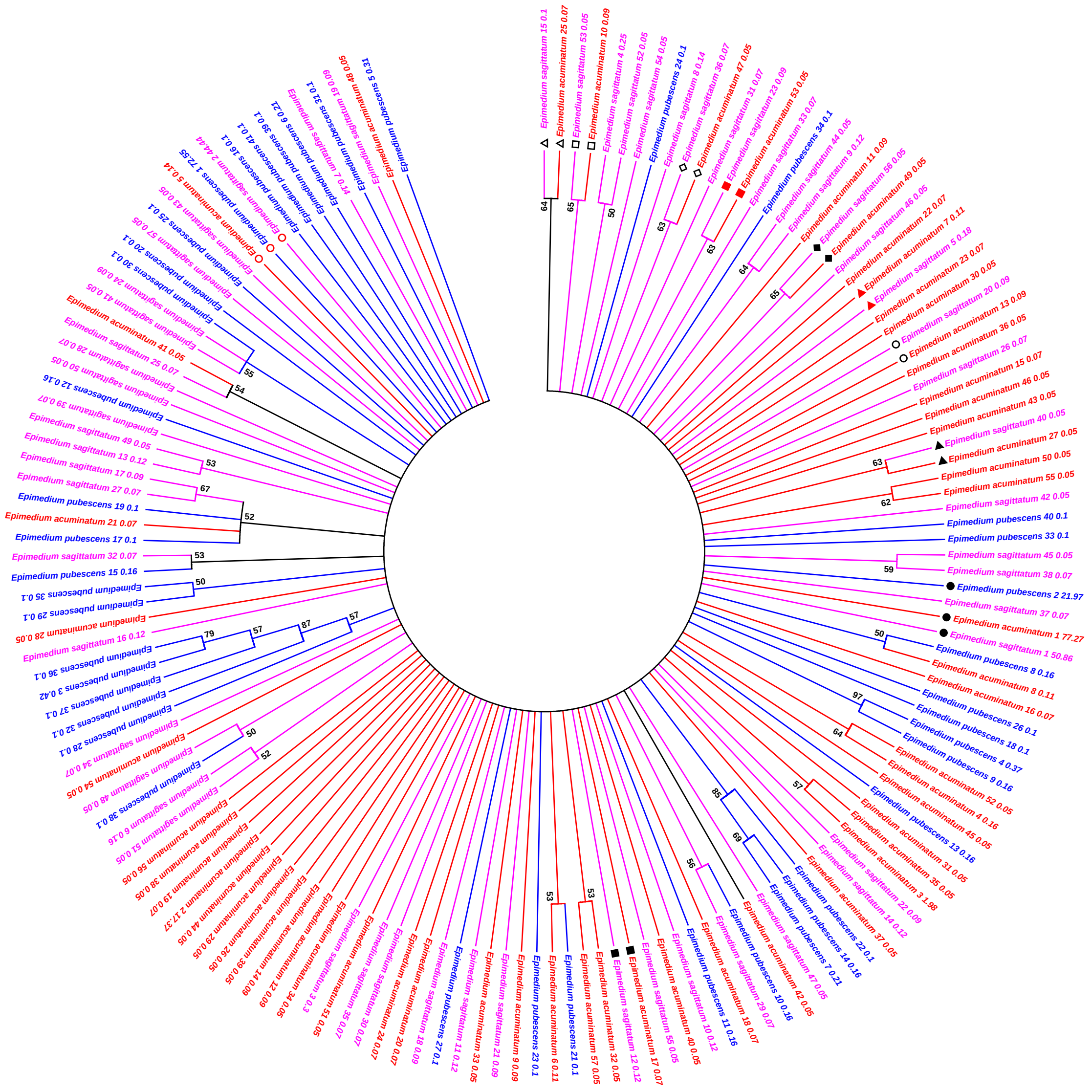

Figure S7B.

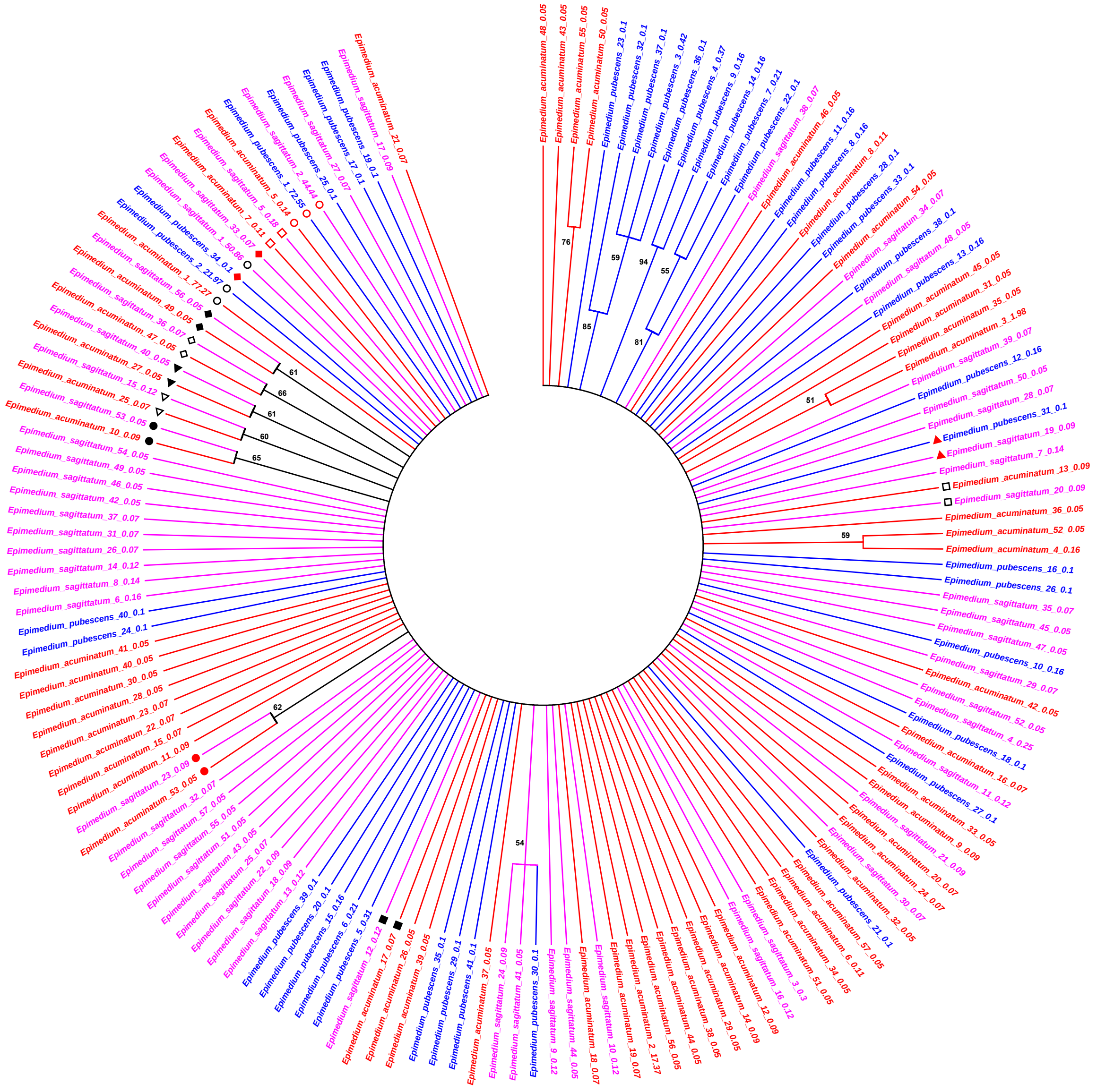

Figure S7C.

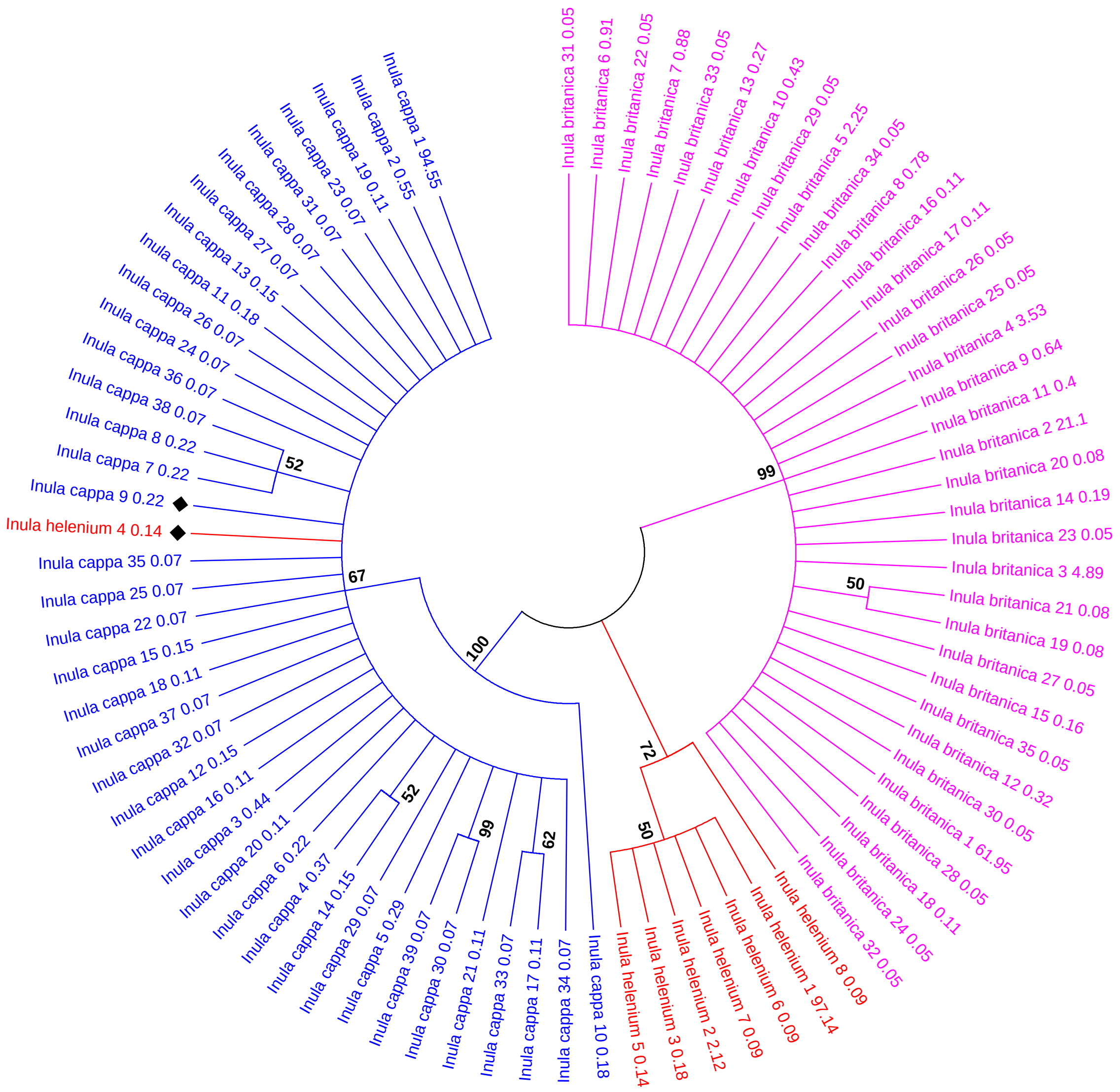
Inula cappa 1 94.55
Inula cappa 2 0.55
Inula cappa 19 0.11
Inula cappa 23 0.07
Inula cappa 31 0.07
Inula cappa 28 0.07
Inula cappa 27 0.07
Inula cappa 13 0.15
Inula cappa 11 0.18
Inula cappa 26 0.07
Inula cappa 24 0.07
Inula cappa 36 0.07
Inula cappa 38 0.07
Inula cappa 8 0.22
Inula cappa 7 0.22
52
Inula cappa 9 0.22
Inula helenium 4 0.14
Inula cappa 35 0.07
67
Inula cappa 25 0.07
Inula cappa 22 0.07
Inula cappa 15 0.15
Inula cappa 18 0.11
Inula cappa 37 0.07
Inula cappa 32 0.07
Inula cappa 12 0.15
Inula cappa 16 0.11
Inula cappa 3 0.44
Inula cappa 20 0.11
Inula cappa 6 0.22
Inula cappa 4 0.37
Inula cappa 14 0.15
52
Inula cappa 29 0.07
Inula cappa 5 0.29
Inula cappa 39 0.07
Inula cappa 30 0.07
99
Inula cappa 21 0.11
Inula cappa 33 0.07
Inula cappa 17 0.11
62
Inula cappa 34 0.07
Inula cappa 10 0.18
100
Inula helenium 5 0.14
Inula helenium 3 0.18
Inula helenium 2 2.12
Inula helenium 7 0.09
Inula helenium 6 0.09
50
Inula helenium 1 97.14
Inula helenium 8 0.09
72
Inula britanica 32 0.05
Inula britanica 24 0.05
Inula britanica 18 0.11
Inula britanica 28 0.05
Inula britanica 1 61.95
Inula britanica 30 0.05
Inula britanica 12 0.32
Inula britanica 35 0.05
Inula britanica 15 0.16
Inula britanica 27 0.05
Inula britanica 19 0.08
Inula britanica 21 0.08
50
Inula britanica 3 4.89
Inula britanica 23 0.05
Inula britanica 14 0.19
Inula britanica 20 0.08
Inula britanica 2 21.1
Inula britanica 11 0.4
Inula britanica 9 0.64
Inula britanica 4 3.53
Inula britanica 25 0.05
Inula britanica 26 0.05
Inula britanica 17 0.11
Inula britanica 16 0.11
Inula britanica 8 0.78
Inula britanica 34 0.05
Inula britanica 5 2.25
Inula britanica 29 0.05
Inula britanica 10 0.43
Inula britanica 13 0.27
Inula britanica 33 0.05
Inula britanica 7 0.88
Inula britanica 22 0.05
Inula britanica 6 0.91
Inula britanica 31 0.05
99

Figure S7D.

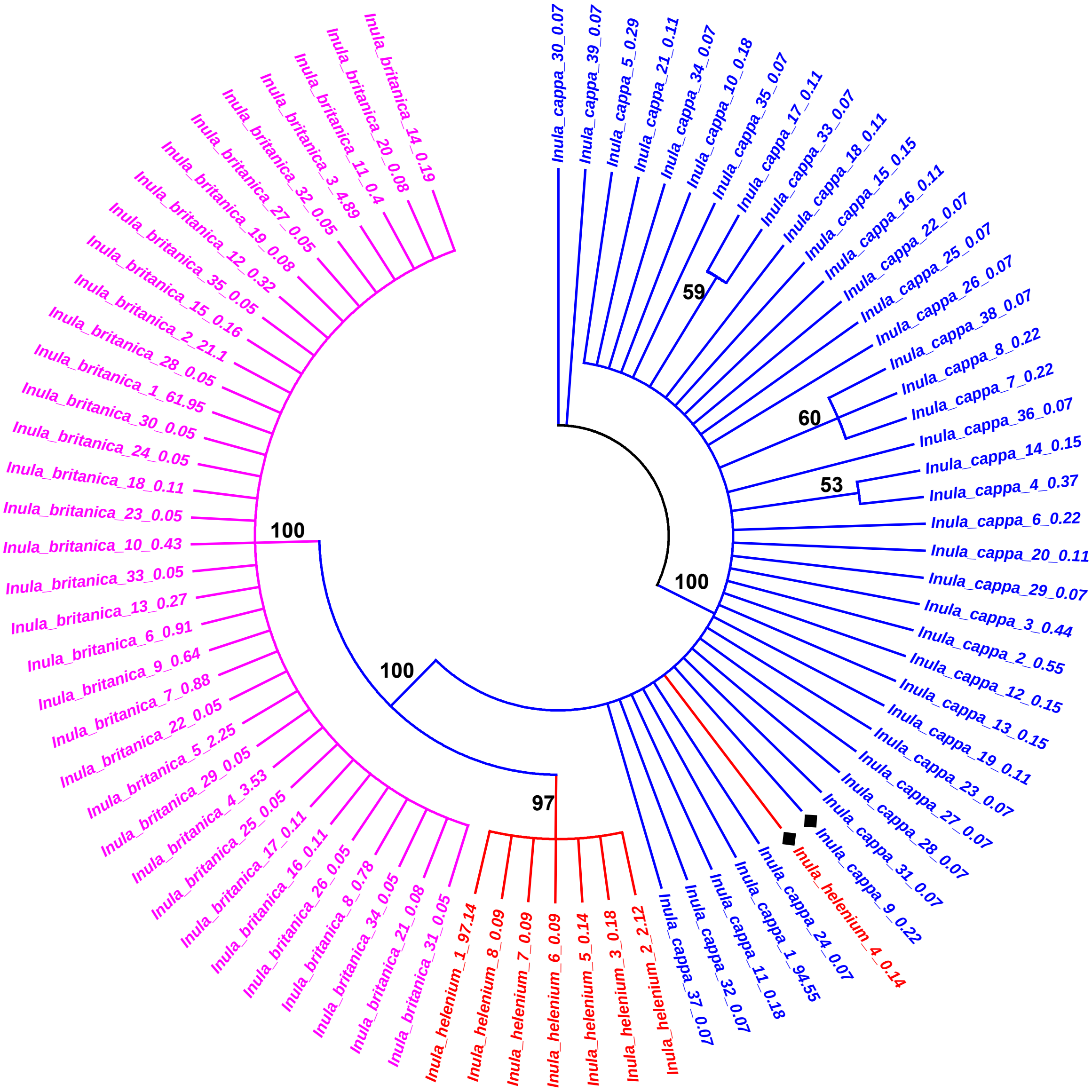
Inula_britanica_14_0.19
Inula_britanica_20_0.08
Inula_britanica_11_0.4
Inula_britanica_3_4.89
Inula_britanica_32_0.05
Inula_britanica_27_0.05
Inula_britanica_19_0.08
Inula_britanica_12_0.32
Inula_britanica_35_0.05
Inula_britanica_15_0.16
Inula_britanica_2_21.1
Inula_britanica_28_0.05
Inula_britanica_1_61.95
Inula_britanica_30_0.05
Inula_britanica_24_0.05
Inula_britanica_18_0.11
Inula_britanica_23_0.05
Inula_britanica_10_0.43
Inula_britanica_33_0.05
Inula_britanica_13_0.27
Inula_britanica_6_0.91
Inula_britanica_9_0.64
Inula_britanica_7_0.88
Inula_britanica_22_0.05
Inula_britanica_5_2.25
Inula_britanica_29_0.05
Inula_britanica_4_3.53
Inula_britanica_25_0.05
Inula_britanica_17_0.11
Inula_britanica_16_0.11
Inula_britanica_26_0.05
Inula_britanica_8_0.78
Inula_britanica_34_0.05
Inula_britanica_21_0.08
Inula_britanica_31_0.05
Inula_helenium_1_97.14
Inula_helenium_8_0.09
Inula_helenium_7_0.09
Inula_helenium_6_0.09
Inula_helenium_5_0.14
Inula_helenium_3_0.18
Inula_helenium_2_2.12
Inula_cappa_37_0.07
Inula_cappa_32_0.07
Inula_cappa_11_0.18
Inula_cappa_1_94.55
Inula_cappa_24_0.07
Inula_helenium_4_0.14
Inula_cappa_9_0.22
Inula_cappa_31_0.07
Inula_cappa_28_0.07
Inula_cappa_27_0.07
Inula_cappa_23_0.07
Inula_cappa_19_0.11
Inula_cappa_13_0.15
Inula_cappa_12_0.15
Inula_cappa_2_0.55
Inula_cappa_3_0.44
Inula_cappa_29_0.07
Inula_cappa_20_0.11
Inula_cappa_6_0.22
Inula_cappa_4_0.37
Inula_cappa_14_0.15
Inula_cappa_36_0.07
Inula_cappa_7_0.22
Inula_cappa_8_0.22
Inula_cappa_38_0.07
Inula_cappa_26_0.07
Inula_cappa_25_0.07
Inula_cappa_22_0.07
Inula_cappa_16_0.11
Inula_cappa_15_0.15
Inula_cappa_18_0.11
Inula_cappa_33_0.07
Inula_cappa_17_0.11
Inula_cappa_35_0.07
Inula_cappa_10_0.18
Inula_cappa_34_0.07
Inula_cappa_21_0.11
Inula_cappa_5_0.29
Inula_cappa_39_0.07
Inula_cappa_30_0.07
100
100
97
100
59
60
53

Figure S7E.

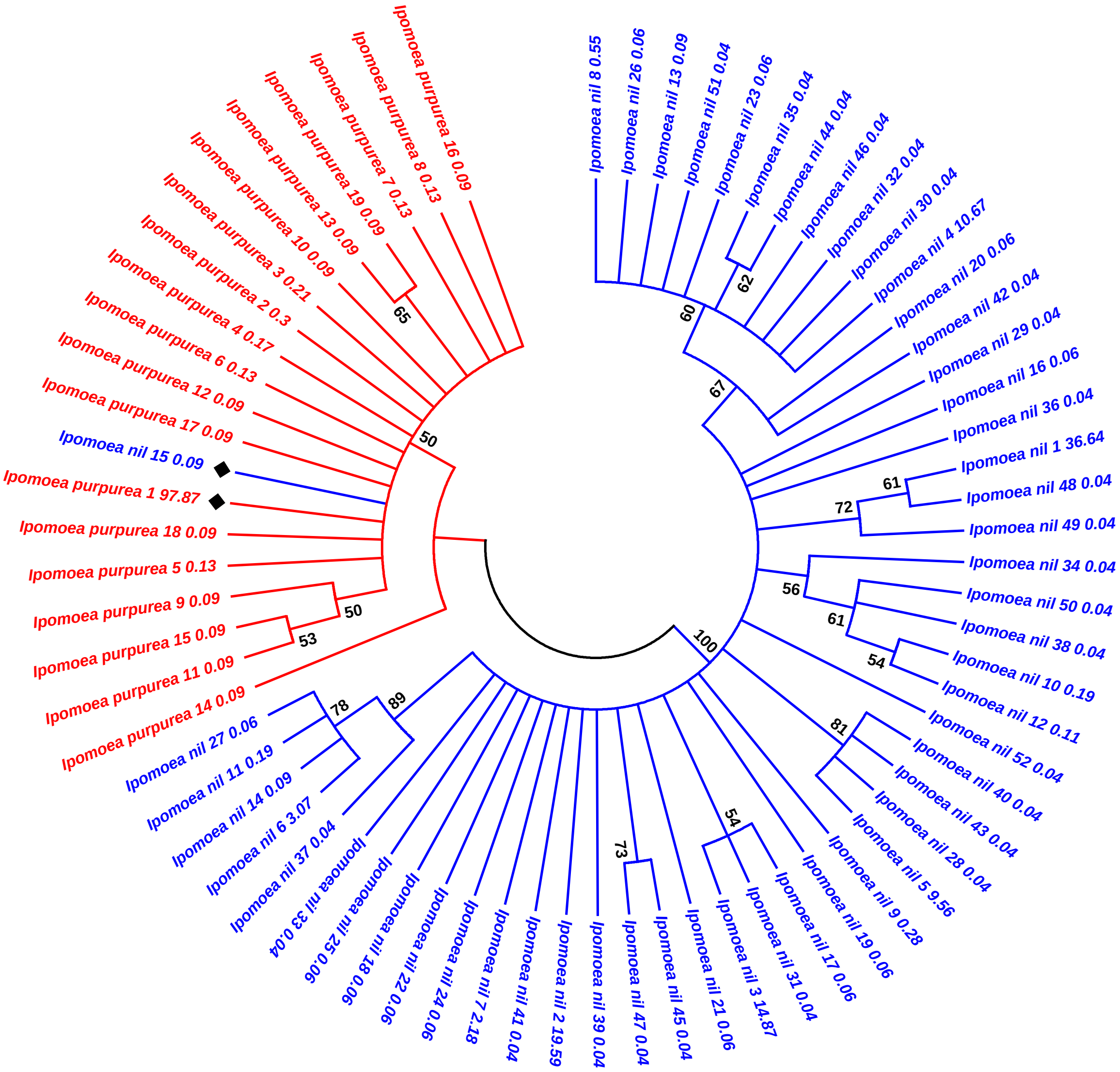
Ipomoea purpurea 16 0.09
Ipomoea purpurea 8 0.13
Ipomoea purpurea 7 0.13
Ipomoea purpurea 19 0.09
Ipomoea purpurea 13 0.09
Ipomoea purpurea 10 0.09
Ipomoea purpurea 3 0.21
Ipomoea purpurea 2 0.3
Ipomoea purpurea 4 0.17
Ipomoea purpurea 6 0.13
Ipomoea purpurea 12 0.09
Ipomoea purpurea 17 0.09
Ipomoea nil 15 0.09
Ipomoea purpurea 1 97.87
Ipomoea purpurea 18 0.09
Ipomoea purpurea 5 0.13
Ipomoea purpurea 9 0.09
Ipomoea purpurea 15 0.09
Ipomoea purpurea 11 0.09
Ipomoea purpurea 14 0.09
Ipomoea nil 27 0.06
Ipomoea nil 11 0.19
Ipomoea nil 14 0.09
Ipomoea nil 6 3.07
Ipomoea nil 37 0.04
Ipomoea nil 33 0.04
Ipomoea nil 25 0.06
Ipomoea nil 18 0.06
Ipomoea nil 22 0.06
Ipomoea nil 24 0.06
Ipomoea nil 7 2.18
Ipomoea nil 41 0.04
Ipomoea nil 2 19.59
Ipomoea nil 39 0.04
Ipomoea nil 47 0.04
Ipomoea nil 45 0.04
Ipomoea nil 21 0.06
Ipomoea nil 3 14.87
Ipomoea nil 31 0.04
Ipomoea nil 17 0.06
Ipomoea nil 19 0.06
Ipomoea nil 9 0.28
Ipomoea nil 5 9.56
Ipomoea nil 28 0.04
Ipomoea nil 43 0.04
Ipomoea nil 40 0.04
Ipomoea nil 52 0.04
Ipomoea nil 12 0.11
Ipomoea nil 10 0.19
Ipomoea nil 38 0.04
Ipomoea nil 50 0.04
Ipomoea nil 34 0.04
Ipomoea nil 49 0.04
Ipomoea nil 48 0.04
Ipomoea nil 1 36.64
Ipomoea nil 36 0.04
Ipomoea nil 16 0.06
Ipomoea nil 29 0.04
Ipomoea nil 42 0.04
Ipomoea nil 20 0.06
Ipomoea nil 4 10.67
Ipomoea nil 30 0.04
Ipomoea nil 32 0.04
Ipomoea nil 46 0.04
Ipomoea nil 44 0.04
Ipomoea nil 35 0.04
Ipomoea nil 23 0.06
Ipomoea nil 51 0.04
Ipomoea nil 13 0.09
Ipomoea nil 26 0.06
Ipomoea nil 8 0.55
65
50
50
53
78
89
73
54
81
100
54
61
56
72
61
67
60
62

Figure S7F.

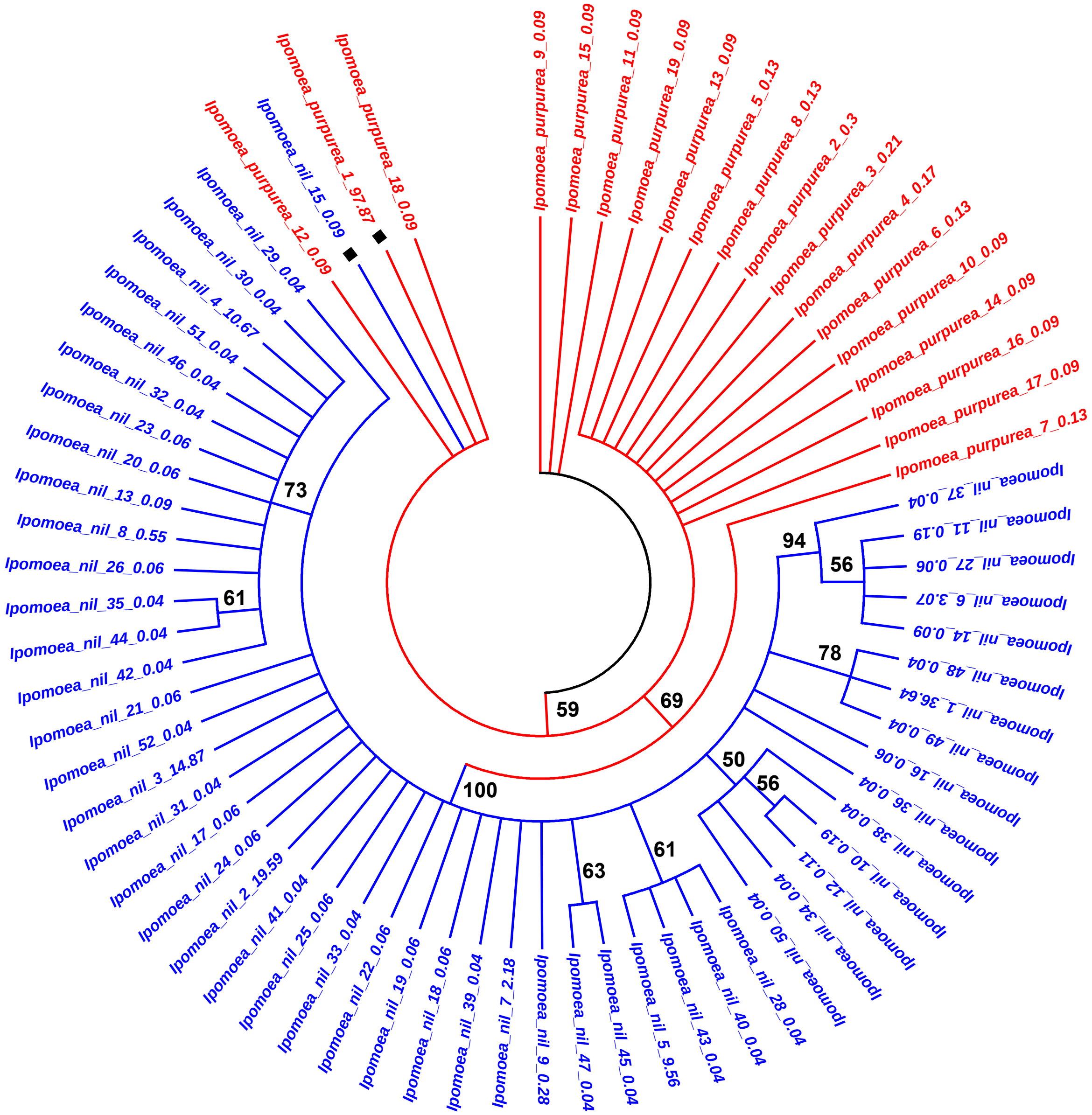
Ipomoea_purpurea_9_0.09
Ipomoea_purpurea_15_0.09
Ipomoea_purpurea_11_0.09
Ipomoea_purpurea_19_0.09
Ipomoea_purpurea_13_0.09
Ipomoea_purpurea_5_0.13
Ipomoea_purpurea_8_0.13
Ipomoea_purpurea_2_0.3
Ipomoea_purpurea_3_0.21
Ipomoea_purpurea_4_0.17
Ipomoea_purpurea_6_0.13
Ipomoea_purpurea_10_0.09
Ipomoea_purpurea_14_0.09
Ipomoea_purpurea_16_0.09
Ipomoea_purpurea_17_0.09
Ipomoea_purpurea_7_0.13
Ipomoea_nil_37_0.04
Ipomoea_nil_11_0.19
Ipomoea_nil_27_0.06
Ipomoea_nil_6_3.07
Ipomoea_nil_14_0.09
Ipomoea_nil_48_0.04
Ipomoea_nil_1_36.64
Ipomoea_nil_49_0.04
Ipomoea_nil_16_0.06
Ipomoea_nil_36_0.04
Ipomoea_nil_38_0.04
Ipomoea_nil_10_0.19
Ipomoea_nil_12_0.11
Ipomoea_nil_34_0.04
Ipomoea_nil_50_0.04
Ipomoea_nil_28_0.04
Ipomoea_nil_40_0.04
Ipomoea_nil_43_0.04
Ipomoea_nil_5_9.56
Ipomoea_nil_45_0.04
Ipomoea_nil_47_0.04
Ipomoea_nil_9_0.28
Ipomoea_nil_7_2.18
Ipomoea_nil_39_0.04
Ipomoea_nil_18_0.06
Ipomoea_nil_19_0.06
Ipomoea_nil_22_0.06
Ipomoea_nil_33_0.04
Ipomoea_nil_25_0.06
Ipomoea_nil_41_0.04
Ipomoea_nil_2_19.59
Ipomoea_nil_24_0.06
Ipomoea_nil_17_0.06
Ipomoea_nil_31_0.04
Ipomoea_nil_3_14.87
Ipomoea_nil_52_0.04
Ipomoea_nil_21_0.06
Ipomoea_nil_42_0.04
Ipomoea_nil_44_0.04
Ipomoea_nil_35_0.04
Ipomoea_nil_26_0.06
Ipomoea_nil_8_0.55
Ipomoea_nil_13_0.09
Ipomoea_nil_20_0.06
Ipomoea_nil_23_0.06
Ipomoea_nil_32_0.04
Ipomoea_nil_46_0.04
Ipomoea_nil_51_0.04
Ipomoea_nil_4_10.67
Ipomoea_nil_30_0.04
Ipomoea_nil_29_0.04
Ipomoea_purpurea_12_0.09
Ipomoea_nil_15_0.09
Ipomoea_purpurea_1_97.87
Ipomoea_purpurea_18_0.09
59
69
94
56
78
50
56
61
63
100
61
73

Figure S7G.

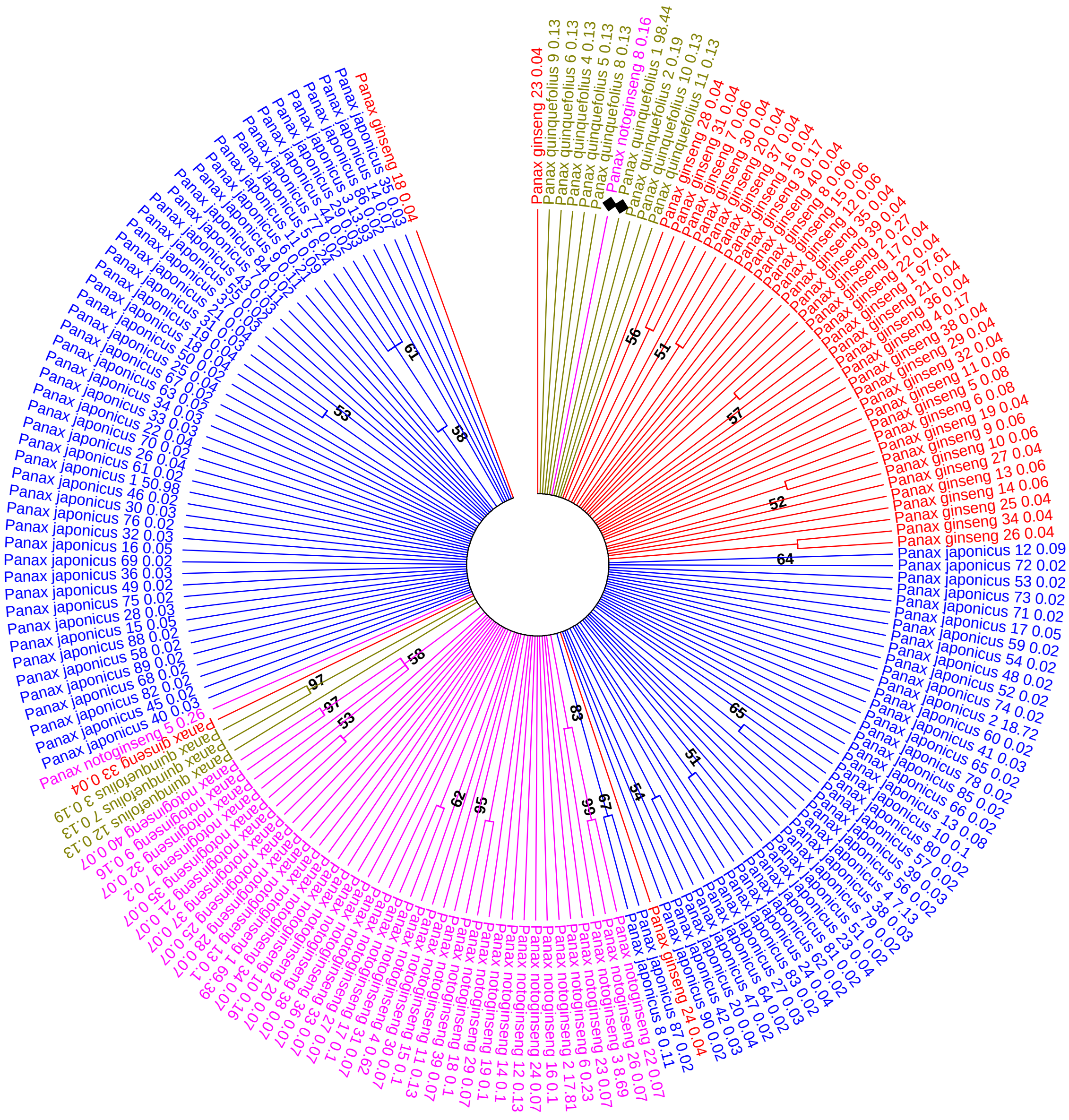

Figure S7H.

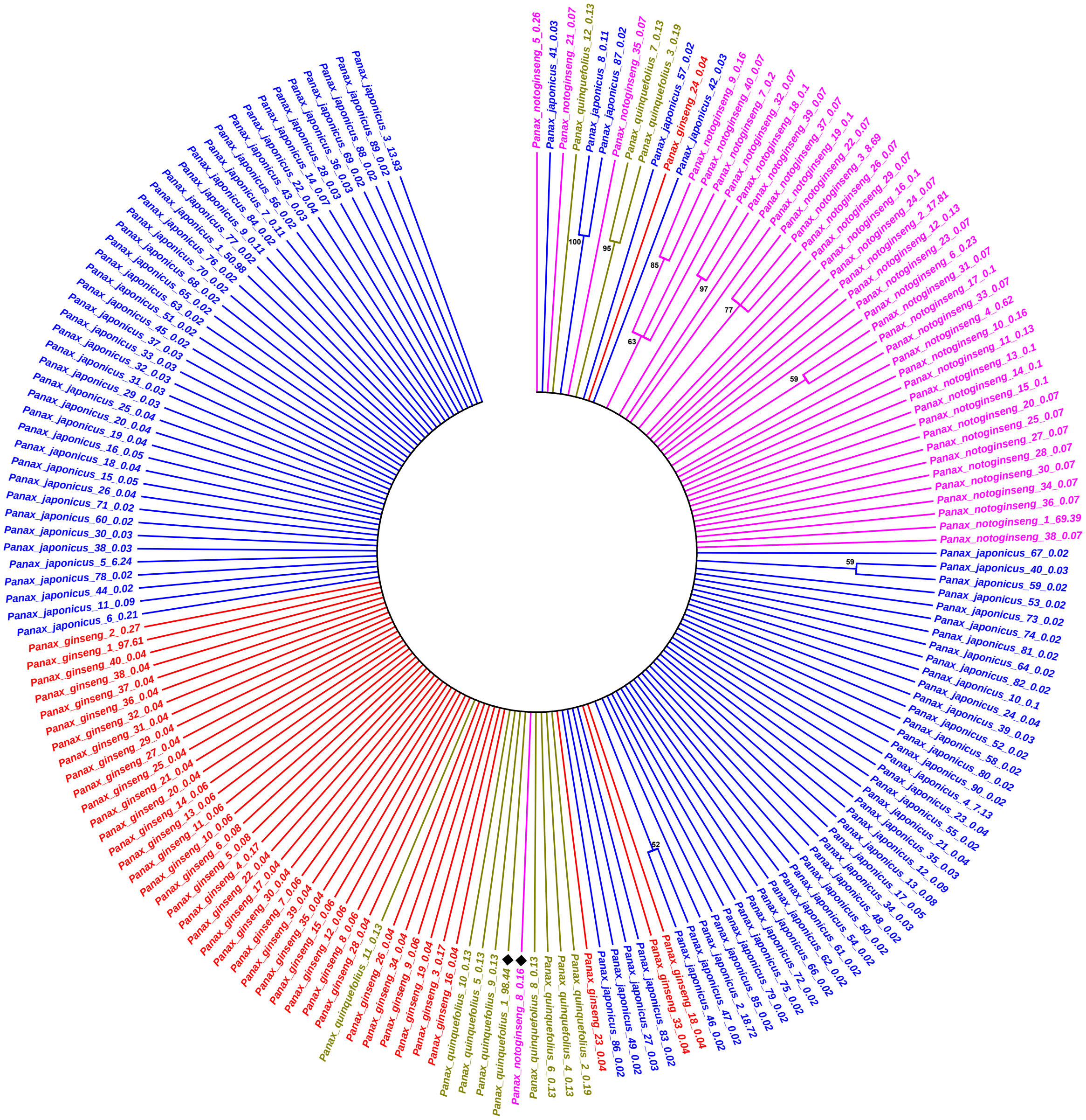

Panax_notoginseng_5_0.26
Panax_japonicus_41_0.03
Panax_notoginseng_21_0.07
Panax_quinquefolius_12_0.13
Panax_japonicus_8_0.11
Panax_japonicus_87_0.02
Panax_notoginseng_35_0.07
Panax_quinquefolius_7_0.13
Panax_quinquefolius_3_0.19
Panax_japonicus_57_0.02
Panax_ginseng_24_0.04
Panax_japonicus_42_0.03
Panax_notoginseng_9_0.16
Panax_notoginseng_40_0.07
Panax_notoginseng_7_0.2
Panax_notoginseng_32_0.07
Panax_notoginseng_18_0.1
Panax_notoginseng_39_0.07
Panax_notoginseng_37_0.07
Panax_notoginseng_19_0.1
Panax_notoginseng_22_0.07
Panax_notoginseng_3_8.69
Panax_notoginseng_26_0.07
Panax_notoginseng_29_0.07
Panax_notoginseng_16_0.1
Panax_notoginseng_24_0.07
Panax_notoginseng_2_17.81
Panax_notoginseng_12_0.13
Panax_notoginseng_23_0.07
Panax_notoginseng_6_0.23
Panax_notoginseng_31_0.07
Panax_notoginseng_17_0.1
Panax_notoginseng_33_0.07
Panax_notoginseng_4_0.62
Panax_notoginseng_10_0.16
Panax_notoginseng_11_0.13
Panax_notoginseng_13_0.1
Panax_notoginseng_14_0.1
Panax_notoginseng_15_0.1
Panax_notoginseng_20_0.07
Panax_notoginseng_25_0.07
Panax_notoginseng_27_0.07
Panax_notoginseng_28_0.07
Panax_notoginseng_30_0.07
Panax_notoginseng_34_0.07
Panax_notoginseng_36_0.07
Panax_notoginseng_1_69.39
Panax_notoginseng_38_0.07
Panax_japonicus_67_0.02
Panax_japonicus_40_0.03
Panax_japonicus_59_0.02
Panax_japonicus_53_0.02
Panax_japonicus_73_0.02
Panax_japonicus_74_0.02
Panax_japonicus_81_0.02
Panax_japonicus_64_0.02
Panax_japonicus_82_0.02
Panax_japonicus_10_0.1
Panax_japonicus_24_0.04
Panax_japonicus_39_0.03
Panax_japonicus_52_0.02
Panax_japonicus_58_0.02
Panax_japonicus_80_0.02
Panax_japonicus_90_0.02
Panax_japonicus_4_7.13
Panax_japonicus_23_0.04
Panax_japonicus_55_0.02
Panax_japonicus_21_0.04
Panax_japonicus_35_0.03
Panax_japonicus_12_0.09
Panax_japonicus_13_0.08
Panax_japonicus_17_0.05
Panax_japonicus_48_0.03
Panax_japonicus_34_0.03
Panax_japonicus_50_0.02
Panax_japonicus_54_0.02
Panax_japonicus_61_0.02
Panax_japonicus_62_0.02
Panax_japonicus_66_0.02
Panax_japonicus_72_0.02
Panax_japonicus_75_0.02
Panax_japonicus_79_0.02
Panax_japonicus_85_0.02
Panax_japonicus_2_18.72
Panax_japonicus_47_0.02
Panax_japonicus_46_0.02
Panax_ginseng_18_0.04
Panax_ginseng_33_0.04
Panax_japonicus_83_0.02
Panax_japonicus_27_0.03
Panax_japonicus_49_0.02
Panax_japonicus_86_0.02
Panax_ginseng_23_0.04
Panax_quinquefolius_2_0.19
Panax_quinquefolius_4_0.13
Panax_quinquefolius_6_0.13
Panax_quinquefolius_8_0.13
Panax_notoginseng_8_0.16
Panax_quinquefolius_1_98.44
Panax_quinquefolius_9_0.13
Panax_quinquefolius_5_0.13
Panax_quinquefolius_10_0.13
Panax_ginseng_16_0.04
Panax_ginseng_3_0.17
Panax_ginseng_19_0.04
Panax_ginseng_9_0.06
Panax_ginseng_34_0.04
Panax_ginseng_26_0.04
Panax_quinquefolius_11_0.13
Panax_ginseng_28_0.04
Panax_ginseng_8_0.06
Panax_ginseng_12_0.06
Panax_ginseng_15_0.06
Panax_ginseng_35_0.04
Panax_ginseng_39_0.04
Panax_ginseng_7_0.06
Panax_ginseng_30_0.04
Panax_ginseng_17_0.04
Panax_ginseng_22_0.04
Panax_ginseng_4_0.17
Panax_ginseng_5_0.08
Panax_ginseng_6_0.08
Panax_ginseng_10_0.06
Panax_ginseng_11_0.06
Panax_ginseng_13_0.06
Panax_ginseng_14_0.06
Panax_ginseng_20_0.04
Panax_ginseng_21_0.04
Panax_ginseng_25_0.04
Panax_ginseng_27_0.04
Panax_ginseng_29_0.04
Panax_ginseng_31_0.04
Panax_ginseng_32_0.04
Panax_ginseng_36_0.04
Panax_ginseng_37_0.04
Panax_ginseng_38_0.04
Panax_ginseng_40_0.04
Panax_ginseng_1_97.61
Panax_ginseng_2_0.27
Panax_japonicus_6_0.21
Panax_japonicus_11_0.09
Panax_japonicus_44_0.02
Panax_japonicus_78_0.02
Panax_japonicus_5_6.24
Panax_japonicus_38_0.03
Panax_japonicus_30_0.03
Panax_japonicus_60_0.02
Panax_japonicus_71_0.02
Panax_japonicus_26_0.04
Panax_japonicus_15_0.05
Panax_japonicus_18_0.04
Panax_japonicus_16_0.05
Panax_japonicus_19_0.04
Panax_japonicus_20_0.04
Panax_japonicus_25_0.04
Panax_japonicus_29_0.03
Panax_japonicus_31_0.03
Panax_japonicus_32_0.03
Panax_japonicus_33_0.03
Panax_japonicus_37_0.02
Panax_japonicus_45_0.02
Panax_japonicus_51_0.02
Panax_japonicus_63_0.02
Panax_japonicus_65_0.02
Panax_japonicus_68_0.02
Panax_japonicus_70_0.02
Panax_japonicus_76_0.02
Panax_japonicus_1_50.98
Panax_japonicus_77_0.02
Panax_japonicus_9_0.11
Panax_japonicus_7_0.11
Panax_japonicus_84_0.02
Panax_japonicus_43_0.03
Panax_japonicus_56_0.02
Panax_japonicus_22_0.04
Panax_japonicus_14_0.07
Panax_japonicus_28_0.03
Panax_japonicus_36_0.03
Panax_japonicus_69_0.02
Panax_japonicus_88_0.02
Panax_japonicus_89_0.02
Panax_japonicus_3_13.93
100
95
85
97
77
63
59
59
52

Figure S7I.

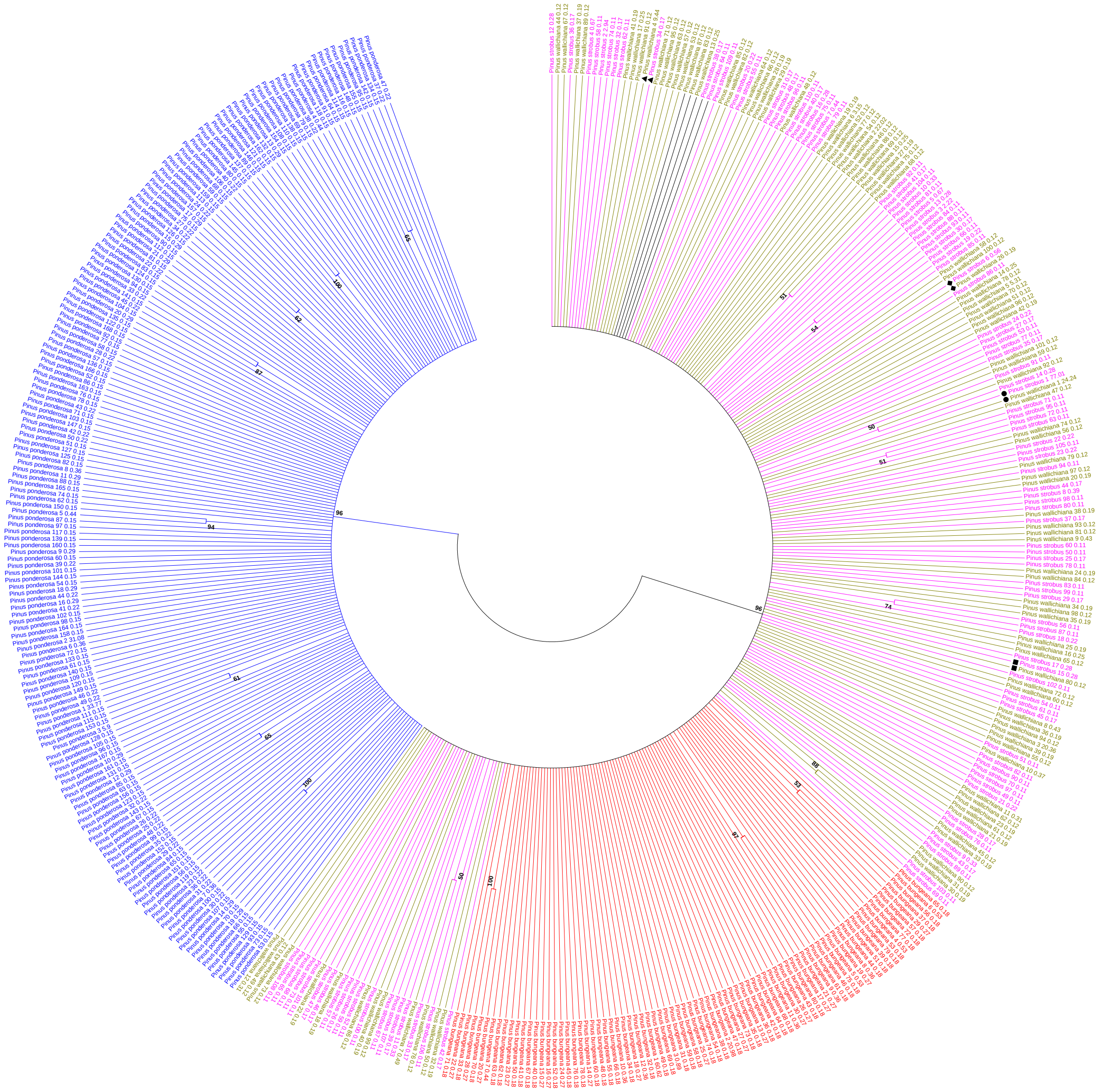

Figure S7J.

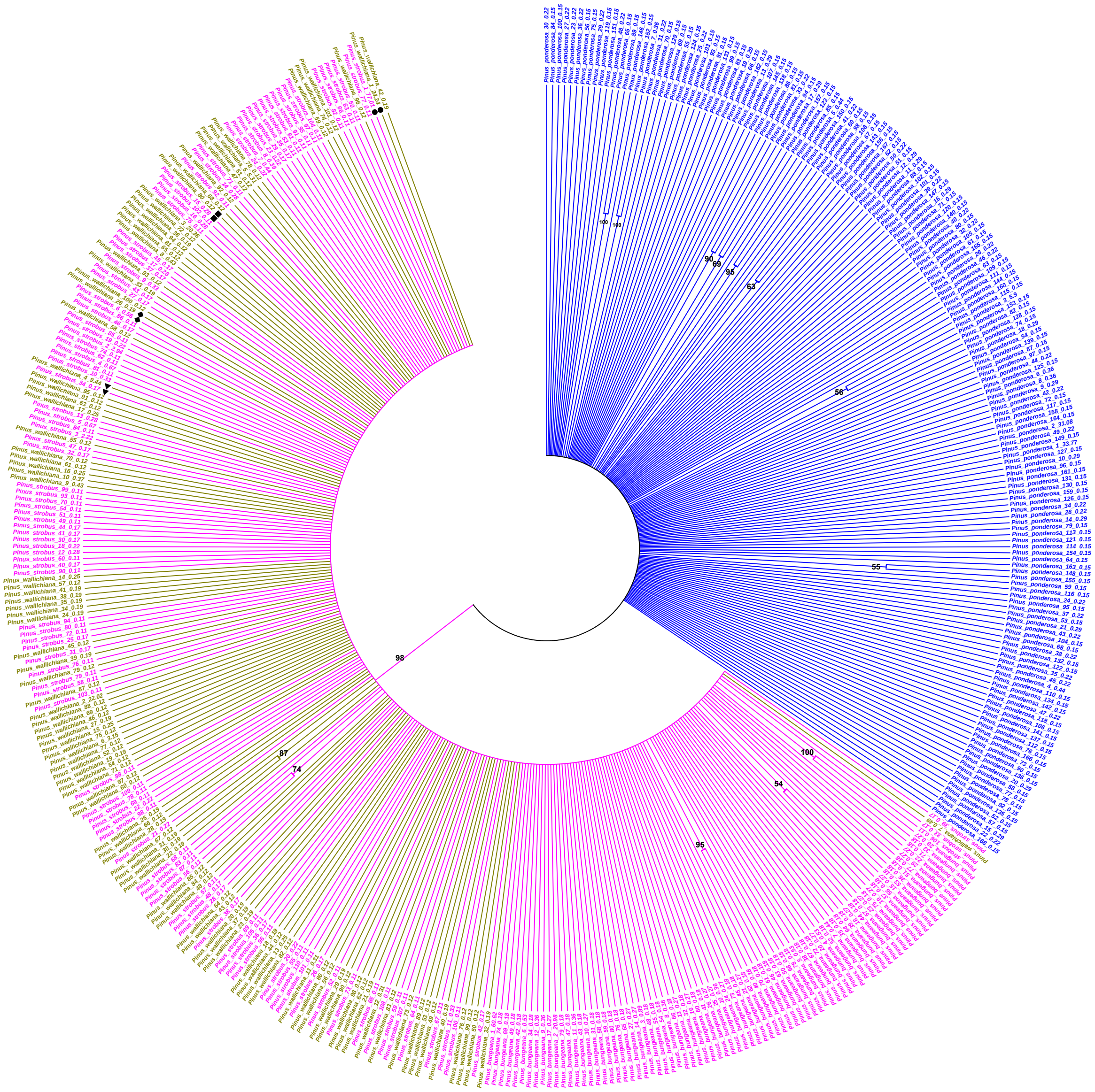

Supplement: Figure S7 — The Neighbor-Joining and the Maximum Parsimony trees of ITS2 variants indicated that genetic information from the common ancestor was maintained in the genomes of descendants. The same colors represent the same species. The same shade and color of symbols show the variants with 100% identity. The Latin names of species are followed by the rank and RVA of the variants. (A,B) Epimedium (Berberidaceae). (C,D) Inula (Asteraceae). (E,F) Ipomoea (Convolvulaceae). (G,H) Panax (Araliaceae). (I,J) Pinus (Pinaceae). (PDF) [file pone.0043971.s007.pdf]
