# Supplementary material for: Extensive Pyrosequencing Reveals Frequent Intra-Genomic Variations of Internal Transcribed Spacer Regions of Nuclear Ribosomal DNA
Source: PLoS One. 2012 Aug 30;7(8):e43971. doi: 10.1371/journal.pone.0043971 (PMC3431384; doi:10.1371/journal.pone.0043971)

Figure S8. The Neighbor-Joining tree of ITS2 variants of four species in the genus *Ilex* (Aquifoliaceae). The same colors represent the same species. The Latin names of species are followed by the rank and RVA of the variants.

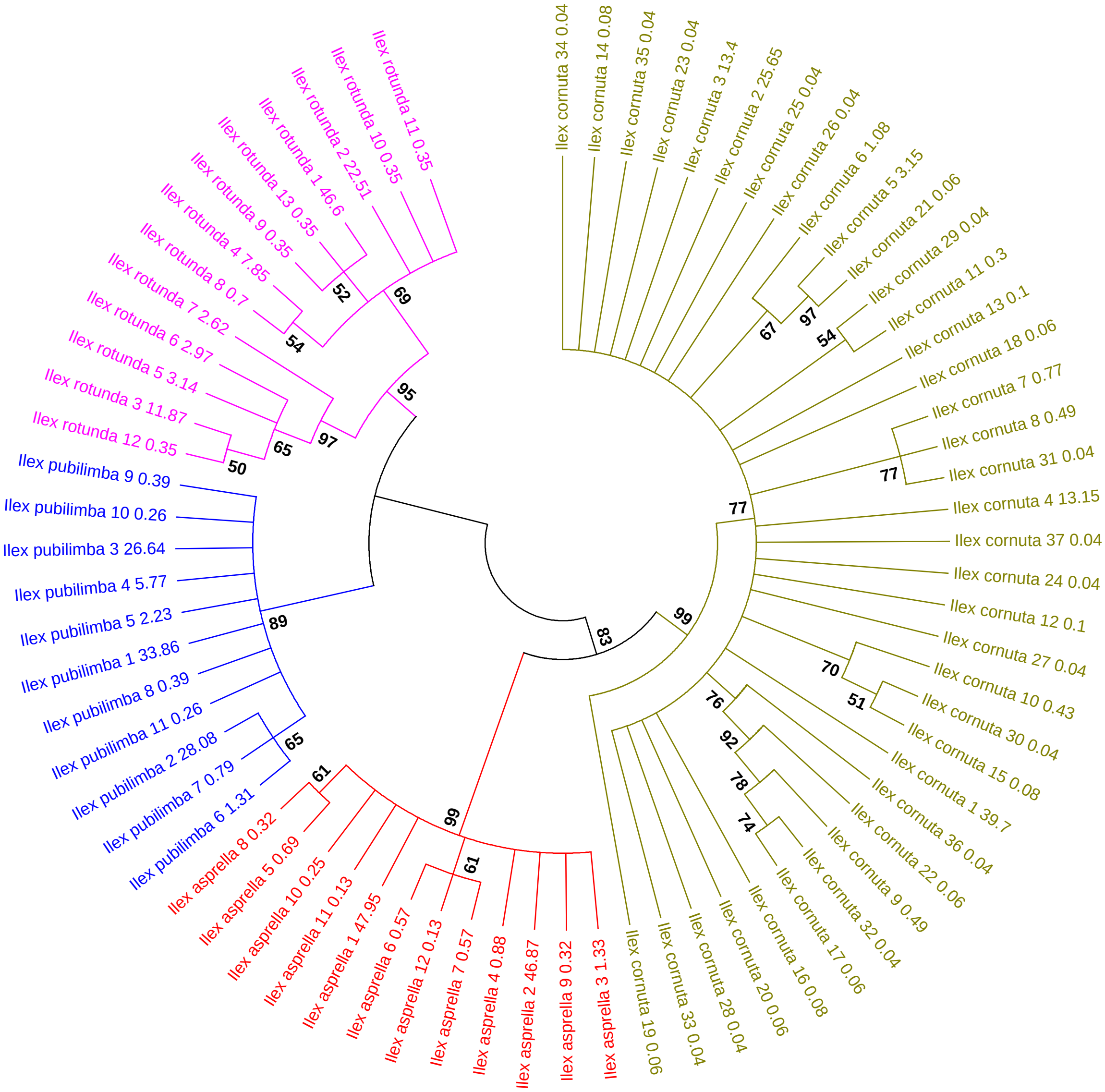

Supplement: Figure S8 — The Neighbor-Joining tree of ITS2 variants of four species in the genus Ilex (Aquifoliaceae). The same colors represent the same species. The Latin names of species are followed by the rank and RVA of the variants. (PDF) [file pone.0043971.s008.pdf]
