# Supplementary material for: Extensive Pyrosequencing Reveals Frequent Intra-Genomic Variations of Internal Transcribed Spacer Regions of Nuclear Ribosomal DNA
Source: PLoS One. 2012 Aug 30;7(8):e43971. doi: 10.1371/journal.pone.0043971 (PMC3431384; doi:10.1371/journal.pone.0043971)
Supplement: Table S1 — Determining the pyrosequencing error rate using known Panax quinquefolius sequences as internal controls. (PDF) [file pone.0043971.s011.pdf]

Table S1. Determining the pyrosequencing error rate using known *Panax quinquefolius* sequences as internal controls.

Panax Quinquefolius Sample Number	Length of Sample (bp)	Number of Reads	Base-Level Error Rate	Sequence-Level Error Rate
1	187	5263	1.30E-04	1.18E-02
2	350	124	9.22E-05	1.61E-02
3	174	2633	1.64E-04	2.58E-02
Average	237	2673	1.29E-04	1.79E-02
