# Supplementary material for: Extensive Pyrosequencing Reveals Frequent Intra-Genomic Variations of Internal Transcribed Spacer Regions of Nuclear Ribosomal DNA
Source: PLoS One. 2012 Aug 30;7(8):e43971. doi: 10.1371/journal.pone.0043971 (PMC3431384; doi:10.1371/journal.pone.0043971)
Supplement: Table S2 — The evolutionary mechanisms of ITS2 variants across 178 plant species under investigation. (PDF) [file pone.0043971.s012.pdf]

Table S2. The evolutionary mechanisms of ITS2 variants across 178 plant species under investigation.

Evolutionary mechanism	Species number	Percentage (%)
Concerted	117	65.7
Birth and Death	48	27.0
Divergent	13	7.3
Total	178	100
