# Supplementary material for: Extensive Pyrosequencing Reveals Frequent Intra-Genomic Variations of Internal Transcribed Spacer Regions of Nuclear Ribosomal DNA
Source: PLoS One. 2012 Aug 30;7(8):e43971. doi: 10.1371/journal.pone.0043971 (PMC3431384; doi:10.1371/journal.pone.0043971)
Supplement: Table S3 — ITS2 variants which have been verified by PCR products cloned or directly sequenced. (PDF) [file pone.0043971.s013.pdf]

Table S3. ITS2 variants which have been verified by PCR products cloned or directly sequenced.

ITS2 variant*	Method	Confirmed (clone number)
Alpinia_hainanensis_1_33.84	Clone	Yes(2)
Alpinia_hainanensis_2_30.06	Clone	Yes(2)
Alpinia_hainanensis_3_28.78	Clone	Yes(5)
Brucea_javanica_1_89.32	PCR	Yes
Brucea_mollis_1_62.64	PCR	Yes
Cerasus_glandulosa_1_14.08	Clone	Yes(9)
Cerasus_glandulosa_6_10.52	Clone	Yes(16)
Cerasus_glandulosa_7_6.26	Clone	Yes(6)
Cerasus_glandulosa_8_3.56	Clone	No
Cerasus_glandulosa_10_2.7	Clone	Yes(1)
Cerasus_glandulosa_12_2.24	Clone	No
Cerasus_japonica_1_20.47	Clone	Yes(15)
Cerasus_japonica_6_8.84	Clone	Yes(7)
Cerasus_japonica_7_8.42	Clone	Yes(6)
Cerasus_japonica_8_4.92	Clone	Yes(3)
Cerasus_japonica_10_2.64	Clone	No
Cerasus_japonica_11_1.78	Clone	No
Citrus_limonum_1_45.53	PCR	Yes
Citrus_maxima_3_8.37	PCR	Yes
Citrus_medica_1_75.75	PCR	Yes
Citrus_reticulata_3_17.46	PCR	Yes
Epimedium_acuminatum_1_77.27	PCR	Yes
Epimedium_pubescens_1_72.55	PCR	Yes
Epimedium_pubescens_2_21.97	PCR	Yes
Epimedium_sagittatum_1_50.86	PCR	Yes
Epimedium_sagittatum_2_44.44	PCR	Yes
Ipomoea_purpurea_1_97.87	PCR	Yes
Ligusticum_jeholense_1_28.63	PCR	Yes
Ligusticum_jeholense_2_22.29	PCR	Yes
Ligusticum_sinense_1_42.72	PCR	Yes
Ligusticum_sinense_2_36.02	PCR	Yes
Ligusticum_sinense_4_4.84	PCR	Yes
Ligusticum_sinense_cv._Chuanxiong_1_40.92	PCR	Yes
Ligusticum_sinense_cv._Chuanxiong_3_11.2	PCR	Yes

Panax_quinquefolius_1_98.44	PCR	Yes
Pinus_strobus_1_77.01	PCR	Yes
Pinus_wallichiana_1_24.24	PCR	Yes
Pinus_wallichiana_4_9.44	PCR	Yes
Potentilla_chinensis_6_4.72	Specific Primer	Yes
Potentilla_nivea__4_2.27	Specific Primer	Yes
Torreya_fargesii_var._yunnanensis_1_94.4	PCR	Yes
Torreya_grandis_2_28.82	PCR	Yes
Torreya_nucifera_1_92.69	PCR	Yes

*Showing the name of species following the rank of the variant and the relative variant abundance.
