# Supplementary material for: Extensive Pyrosequencing Reveals Frequent Intra-Genomic Variations of Internal Transcribed Spacer Regions of Nuclear Ribosomal DNA
Source: PLoS One. 2012 Aug 30;7(8):e43971. doi: 10.1371/journal.pone.0043971 (PMC3431384; doi:10.1371/journal.pone.0043971)
Supplement: Table S4 — Samples and their voucher numbers. (PDF) [file pone.0043971.s014.pdf]

Table S4. Samples and their voucher numbers.

No.	Division	Family	Species	Voucher Number
1	Angiosperms	Acoraceae	*Acorus calamus*	PS1418MT03
2	Angiosperms	Acoraceae	*Acorus calamus*	PS1418MT04
3	Angiosperms	Acoraceae	*Acorus gramineus*	PS1301MT04
4	Angiosperms	Acoraceae	*Acorus gramineus*	PS1301MT05
5	Angiosperms	Amaranthaceae	*Celosia argentea*	PS1495MT02
6	Angiosperms	Amaranthaceae	*Celosia cristata*	PS1491MT02
7	Angiosperms	Anacardiaceae	*Rhus chinensis*	PS1014MT03
8	Angiosperms	Apiaceae	*Angelica dahurica*	PS1197MT03
9	Angiosperms	Apiaceae	*Angelica dahurica*	PS1197MT01
10	Angiosperms	Apiaceae	*Angelica decursiva*	PS1226MT04
11	Angiosperms	Apiaceae	*Angelica decursiva*	PS1226MT05
12	Angiosperms	Apiaceae	*Angelica sinensis*	PS1205MT01
13	Angiosperms	Apiaceae	*Cryptotaenia japonica*	PS1223MT01
14	Angiosperms	Apiaceae	*Daucus carota*	PS1225MT02
15	Angiosperms	Apiaceae	*Foeniculum vulgare*	PS1222MT03
16	Angiosperms	Apiaceae	*Ligusticum jeholense*	PS1213MT02
17	Angiosperms	Apiaceae	*Ligusticum sinense*	PS1208MT01
18	Angiosperms	Apiaceae	*Ligusticum sinense* cv. *Chuanxiong*	PS1203MT01
19	Angiosperms	Apiaceae	*Ligusticum sinense* cv. *Chuanxiong*	PS1203MT02
20	Angiosperms	Aquifoliaceae	*Ilex asprella*	PS0325MT01
21	Angiosperms	Aquifoliaceae	*Ilex cornuta*	PS0320MT01
22	Angiosperms	Aquifoliaceae	*Ilex cornuta*	PS0320MT03
23	Angiosperms	Aquifoliaceae	*Ilex cornuta*	PS0320MT05
24	Angiosperms	Aquifoliaceae	*Ilex pubilimba*	PS0324MT01
25	Angiosperms	Aquifoliaceae	*Ilex rotunda*	PS0321MT01
26	Angiosperms	Araliaceae	*Eleutherococcus giraldii*	PS1461MT01
27	Angiosperms	Araliaceae	*Eleutherococcus nodiflorus*	PS1473MT01
28	Angiosperms	Araliaceae	*Eleutherococcus senticosus*	PS1456MT03
29	Angiosperms	Araliaceae	*Panax ginseng*	PS1467MT01
30	Angiosperms	Araliaceae	*Panax japonicus*	PS1477MT01
31	Angiosperms	Araliaceae	*Panax japonicus*	PS1477MT02
32	Angiosperms	Araliaceae	*Panax japonicus*	PS1477MT03
33	Angiosperms	Araliaceae	*Panax japonicus*	PS1477MT04

34	Angiosperms	Araliaceae	*Panax notoginseng*	PS1469MT02
35	Angiosperms	Araliaceae	*Panax notoginseng*	PS1469MT03
36	Angiosperms	Araliaceae	*Panax quinquefolius*	PS1472MT01
37	Angiosperms	Asclepiadaceae	*Cynanchum atratum*	PS0833MT01
38	Angiosperms	Asclepiadaceae	*Cynanchum paniculatum*	PS0836MT01
39	Angiosperms	Asclepiadaceae	*Cynanchum stauntonii*	PS0835MT04
40	Angiosperms	Asclepiadaceae	*Periploca sepium*	PS0840MT03
41	Angiosperms	Asteraceae	*Artemisia annua*	PS0633MT04
42	Angiosperms	Asteraceae	*Artemisia annua*	PS0633MT05
43	Angiosperms	Asteraceae	*Artemisia argyi*	PS0590MT04
44	Angiosperms	Asteraceae	*Artemisia capillaris*	PS0712MT02
45	Angiosperms	Asteraceae	*Artemisia gmelinii*	PS0594MT01
46	Angiosperms	Asteraceae	*Artemisia lavandulaefolia*	PS0703MT01
47	Angiosperms	Asteraceae	*Aster ageratoides*	PS0680MT01
48	Angiosperms	Asteraceae	*Aster tataricus*	PS0721MT03
49	Angiosperms	Asteraceae	*Cirsium japonicum*	PS0612MT02
50	Angiosperms	Asteraceae	*Cirsium japonicum*	PS0612MT03
51	Angiosperms	Asteraceae	*Cirsium japonicum*	PS0612MT06
52	Angiosperms	Asteraceae	*Cirsium setosum*	PS0611MT01
53	Angiosperms	Asteraceae	*Cirsium setosum*	PS0611MT02
54	Angiosperms	Asteraceae	*Cirsium setosum*	PS0611MT04
55	Angiosperms	Asteraceae	*Eupatorium fortunei*	PS0672MT01
56	Angiosperms	Asteraceae	*Inula britanica*	PS0670MT01
57	Angiosperms	Asteraceae	*Inula britanica*	PS0670MT02
58	Angiosperms	Asteraceae	*Inula cappa*	PS0701MT01
59	Angiosperms	Asteraceae	*Inula helenium*	PS0689MT01
60	Angiosperms	Asteraceae	*Prenanthes tatarinowii*	PS0671MT01
61	Angiosperms	Asteraceae	*Siegesbeckia glabrescens*	PS0660MT01
62	Angiosperms	Asteraceae	*Siegesbeckia orientalis*	PS0618MT05
63	Angiosperms	Asteraceae	*Stemmacantha uniflora*	PS0674MT01
64	Angiosperms	Berberidaceae	*Epimedium acuminatum*	PS1499MT01
65	Angiosperms	Berberidaceae	*Epimedium acuminatum*	PS1499MT02
66	Angiosperms	Berberidaceae	*Epimedium pubescens*	PS1505MT01
67	Angiosperms	Berberidaceae	*Epimedium sagittatum*	PS1502MT02
68	Angiosperms	Berberidaceae	*Epimedium sagittatum*	PS1502MT03
69	Angiosperms	Brassicaceae	*Arabidopsis thaliana*	PS9001MT01
70	Angiosperms	Brassicaceae	*Arabidopsis thaliana*	PS9001MT02
71	Angiosperms	Brassicaceae	*Arabidopsis thaliana*	PS9001MT03
72	Angiosperms	Caprifoliaceae	*Lonicera confusa*	PS1161MT03

73	Angiosperms	Caprifoliaceae	*Lonicera japonica*	PS1165MT02
74	Angiosperms	Caryophyllaceae	*Stellaria vestita*	PS1317MT01
75	Angiosperms	Convolvulaceae	*Ipomoea nil*	PS1536MT01
76	Angiosperms	Convolvulaceae	*Ipomoea nil*	PS1536MT05
77	Angiosperms	Convolvulaceae	*Ipomoea purpurea*	PS1542MT05
78	Angiosperms	Crassulaceae	*Sedum emarginatum*	PS0574MT03
79	Angiosperms	Crassulaceae	*Sedum lineare*	PS0577MT01
80	Angiosperms	Crassulaceae	*Sedum sarmentosum*	PS0575MT01
81	Angiosperms	Crassulaceae	*Sedum sarmentosum*	PS0575MT02
82	Angiosperms	Cucurbitaceae	*Siraitia grosvenorii*	PS0464MT02
83	Angiosperms	Euphorbiaceae	*Euphorbia esula*	PS0193MT01
84	Angiosperms	Euphorbiaceae	*Euphorbia hirta*	PS0175MT02
85	Angiosperms	Euphorbiaceae	*Euphorbia hirta*	PS0175MT03
86	Angiosperms	Euphorbiaceae	*Euphorbia pekinensis*	PS0187MT01
87	Angiosperms	Fabaceae	*Astragalus chinensis*	PS0272MT01
88	Angiosperms	Fabaceae	*Astragalus hancockii*	PS0271MT01
89	Angiosperms	Fabaceae	*Astragalus mongholicus*	PS0277MT01
90	Angiosperms	Fabaceae	*Flemingia lineata*	PS0223MT01
91	Angiosperms	Fabaceae	*Flemingia macrophylla*	PS0222MT02
92	Angiosperms	Fabaceae	*Flemingia macrophylla*	PS0222MT03
93	Angiosperms	Fabaceae	*Pueraria montana* var. *lobata*	PS0313MT04
94	Angiosperms	Fabaceae	*Pueraria peduncularis*	PS0314MT01
95	Angiosperms	Fabaceae	*Senna alata*	PS1362MT02
96	Angiosperms	Fabaceae	*Senna obtusifolia*	PS1588MT08
97	Angiosperms	Fabaceae	*Sophora flavescens*	PS0247MT04
98	Angiosperms	Fabaceae	*Sophora japonica*	PS0241MT03
99	Angiosperms	Fabaceae	*Sophora tonkinensis*	PS0228MT01
100	Angiosperms	Gentianaceae	*Gentiana macrophylla*	PS0821MT02
101	Angiosperms	Gentianaceae	*Gentiana manshurica*	PS0823MT01
102	Angiosperms	Gentianaceae	*Gentiana rigescens*	PS0822MT01
103	Angiosperms	Gentianaceae	*Gentiana straminea*	PS0820MT01
104	Angiosperms	Gentianaceae	*Gentiana straminea*	PS0820MT02
105	Angiosperms	Liliaceae	*Allium tuberosum*	PS0038MT01
106	Angiosperms	Liliaceae	*Allium tuberosum*	PS0038MT02
107	Angiosperms	Liliaceae	*Allium tuberosum*	PS0038MT03
108	Angiosperms	Liliaceae	*Allium tuberosum*	PS0038MT05
109	Angiosperms	Liliaceae	*Aloe vera*	PS0044MT01
110	Angiosperms	Liliaceae	*Aloe vera*	PS0044MT02
111	Angiosperms	Liliaceae	*Aloe vera*	PS0044MT03

112	Angiosperms	Liliaceae	*Asparagus cochinchinensis*	PS0057MT01
113	Angiosperms	Liliaceae	*Asparagus cochinchinensis*	PS0057MT04
114	Angiosperms	Liliaceae	*Asparagus cochinchinensis*	PS0057MT05
115	Angiosperms	Liliaceae	*Asparagus cochinchinensis*	PS0057MT06
116	Angiosperms	Liliaceae	*Asparagus schoberioides*	PS0058MT01
117	Angiosperms	Liliaceae	*Asparagus trichophyllus*	PS0059MT01
118	Angiosperms	Liliaceae	*Asparagus trichophyllus*	PS0059MT02
119	Angiosperms	Liliaceae	*Lilium pumilum*	PS0064MT01
120	Angiosperms	Liliaceae	*Lilium tigrinum*	PS0039MT01
121	Angiosperms	Liliaceae	*Liriope spicata*	PS0055MT01
122	Angiosperms	Myrsinaceae	*Ardisia crenata*	PS1658MT02
123	Angiosperms	Myrsinaceae	*Ardisia crenata*	PS1658MT03
124	Angiosperms	Myrsinaceae	*Ardisia japonica*	PS1659MT05
125	Angiosperms	Orchidaceae	*Dendrobium capillipes*	PS2502MT01
126	Angiosperms	Orchidaceae	*Dendrobium catenatum*	PS2521MT01
127	Angiosperms	Orchidaceae	*Dendrobium chrysanthum*	PS2515MT01
128	Angiosperms	Orchidaceae	*Dendrobium chrysotoxum*	PS2501MT01
129	Angiosperms	Orchidaceae	*Dendrobium chrysotoxum*	PS2501MT03
130	Angiosperms	Orchidaceae	*Dendrobium crepidatum*	PS2517MT01
131	Angiosperms	Orchidaceae	*Dendrobium crystallinum*	PS2519MT01
132	Angiosperms	Orchidaceae	*Dendrobium cucullatum*	PS2523MT01
133	Angiosperms	Orchidaceae	*Dendrobium denneanum*	PS0758MT01
134	Angiosperms	Orchidaceae	*Dendrobium devonianum*	PS2520MT01
135	Angiosperms	Orchidaceae	*Dendrobium gratiosissimum*	PS2513MT01
136	Angiosperms	Orchidaceae	*Dendrobium loddigesii*	PS1748MT02
137	Angiosperms	Orchidaceae	*Dendrobium nobile*	PS0766MT04
138	Angiosperms	Orchidaceae	*Dendrobium nobile*	PS0766MT06
139	Angiosperms	Orchidaceae	*Dendrobium pendulum*	PS2511MT01
140	Angiosperms	Orchidaceae	*Dendrobium polyanthum*	PS2518MT01
141	Angiosperms	Orchidaceae	*Dendrobium polyanthum*	PS2518MT02
142	Angiosperms	Orchidaceae	*Dendrobium spatella*	PS2527MT01
143	Angiosperms	Orchidaceae	*Dendrobium trigonopus*	PS2506MT01
144	Angiosperms	Orchidaceae	*Dendrobium wardianum*	PS2509MT01
145	Angiosperms	Orchidaceae	*Dendrobium wardianum*	PS2509MT02
146	Angiosperms	Orchidaceae	*Dendrobium williamsonii*	PS2503MT01
147	Angiosperms	Paeoniaceae	*Paeonia anomala* ssp. *veitchii*	PS0906MT01
148	Angiosperms	Paeoniaceae	*Paeonia anomala* ssp. *veitchii*	PS0906MT02
149	Angiosperms	Paeoniaceae	*Paeonia lactiflora*	PS0905MT01
150	Angiosperms	Paeoniaceae	*Paeonia ostii*	PS0913MT02

151	Angiosperms	Piperaceae	*Piper longum*	PS0445MT01
152	Angiosperms	Piperaceae	*Piper longum*	PS0445MT02
153	Angiosperms	Piperaceae	*Piper nigrum*	PS0449MT02
154	Angiosperms	Poaceae	*Oryza sativa* ssp. *indica*	PS9003MT01
155	Angiosperms	Poaceae	*Oryza sativa* ssp. *indica*	PS9003MT02
156	Angiosperms	Poaceae	*Oryza sativa* ssp. *indica*	PS9003MT03
157	Angiosperms	Poaceae	*Oryza sativa* ssp. *japonica*	PS9002MT01
158	Angiosperms	Poaceae	*Oryza sativa* ssp. *japonica*	PS9002MT02
159	Angiosperms	Poaceae	*Zea mays*	PS9005MT01
160	Angiosperms	Poaceae	*Zea mays*	PS9005MT02
161	Angiosperms	Poaceae	*Zea mays*	PS9005MT03
162	Angiosperms	Polygonaceae	*Polygonum chinense*	PS0794MT01
163	Angiosperms	Polygonaceae	*Polygonum chinense*	PS0794MT02
164	Angiosperms	Ranunculaceae	*Cimicifuga dahurica*	PS0929MT02
165	Angiosperms	Ranunculaceae	*Cimicifuga foetida*	PS0925MT01
166	Angiosperms	Ranunculaceae	*Cimicifuga foetida*	PS0925MT02
167	Angiosperms	Rosaceae	*Amygdalus persica*	PS1117MT02
168	Angiosperms	Rosaceae	*Amygdalus triloba*	PS1127MT02
169	Angiosperms	Rosaceae	*Armeniaca sibirica*	PS1121MT04
170	Angiosperms	Rosaceae	*Armeniaca vulgaris*	PS1123MT01
171	Angiosperms	Rosaceae	*Armeniaca vulgaris*	PS1123MT02
172	Angiosperms	Rosaceae	*Cerasus glandulosa*	PS1088MT01
173	Angiosperms	Rosaceae	*Cerasus japonica*	PS1128MT01
174	Angiosperms	Rosaceae	*Cerasus tomentosa*	PS1092MT01
175	Angiosperms	Rosaceae	*Potentilla chinensis*	PS1120MT01
176	Angiosperms	Rosaceae	*Potentilla chinensis*	PS1120MT02
177	Angiosperms	Rosaceae	*Potentilla fruticosa*	PS1084MT01
178	Angiosperms	Rosaceae	*Potentilla nivea*	PS1124MT01
179	Angiosperms	Rosaceae	*Potentilla supina*	PS1067MT01
180	Angiosperms	Rosaceae	*Potentilla supina*	PS1067MT02
181	Angiosperms	Rosaceae	*Rosa bella*	PS1098MT01
182	Angiosperms	Rosaceae	*Rosa laevigata*	PS1085MT01
183	Angiosperms	Rosaceae	*Rubus chingii*	PS1135MT01
184	Angiosperms	Rosaceae	*Rubus parvifolius*	PS1093MT01
185	Angiosperms	Rubiaceae	*Uncaria macrophylla*	PS1038MT01
186	Angiosperms	Rubiaceae	*Uncaria macrophylla*	PS1038MT03
187	Angiosperms	Rubiaceae	*Uncaria macrophylla*	PS1038MT04
188	Angiosperms	Rubiaceae	*Uncaria sessilifructus*	PS1041MT02
189	Angiosperms	Rubiaceae	*Uncaria sinensis*	PS1039MT01

190	Angiosperms	Rutaceae	*Citrus aurantium*	PS1613MT01
191	Angiosperms	Rutaceae	*Citrus limonum*	PS1609MT01
192	Angiosperms	Rutaceae	*Citrus maxima*	PS1600MT04
193	Angiosperms	Rutaceae	*Citrus maxima*	PS1600MT05
194	Angiosperms	Rutaceae	*Citrus maxima*	PS1600MT06
195	Angiosperms	Rutaceae	*Citrus maxima*	PS1600MT08
196	Angiosperms	Rutaceae	*Citrus maxima*	PS1600MT09
197	Angiosperms	Rutaceae	*Citrus medica*	PS1616MT02
198	Angiosperms	Rutaceae	*Citrus reticulata*	PS1596MT01
199	Angiosperms	Rutaceae	*Melicope pteleifolia*	PS1610MT01
200	Angiosperms	Rutaceae	*Tetradium ruticarpum*	PS1614MT01
201	Angiosperms	Rutaceae	*Zanthoxylum bungeanum*	PS1599MT01
202	Angiosperms	Salicaceae	*Populus trichocarpa*	PS9004MT01
203	Angiosperms	Santalaceae	*Santalum album*	PS1377MT03
204	Angiosperms	Saxifragaceae	*Dichroa febrifuga*	PS0850MT01
205	Angiosperms	Scrophulariaceae	*Veronicastrum axillare*	PS1525MT01
206	Angiosperms	Scrophulariaceae	*Veronicastrum stenostachyum*	PS1519MT01
207	Angiosperms	Simaroubaceae	*Brucea javanica*	PS0753MT01
208	Angiosperms	Simaroubaceae	*Brucea javanica*	PS0753MT02
209	Angiosperms	Simaroubaceae	*Brucea javanica*	PS0753MT05
210	Angiosperms	Simaroubaceae	*Brucea mollis*	PS0752MT01
211	Angiosperms	Solanaceae	*Datura arborea*	PS1147MT01
212	Angiosperms	Solanaceae	*Datura metel*	PS1152MT02
213	Angiosperms	Solanaceae	*Solanum lyratum*	PS1137MT01
214	Angiosperms	Solanaceae	*Solanum nigrum*	PS1144MT02
215	Angiosperms	Urticaceae	*Boehmeria nivea*	PS1031MT01
216	Angiosperms	Urticaceae	*Boehmeria nivea*	PS1031MT05
217	Angiosperms	Verbenaceae	*Clerodendrum bungei*	PS0853MT01
218	Angiosperms	Verbenaceae	*Clerodendrum cytophyllum*	PS0856MT02
219	Angiosperms	Verbenaceae	*Clerodendrum japonicum*	PS0851MT01
220	Angiosperms	Verbenaceae	*Verbena officinalis*	PS0865MT03
221	Angiosperms	Violaceae	*Viola diffusa*	PS0555MT01
222	Angiosperms	Violaceae	*Viola philippica*	PS0561MT02
223	Angiosperms	Zingiberaceae	*Alpinia galanga*	PS0515MT02
224	Angiosperms	Zingiberaceae	*Alpinia galanga*	PS0515MT03
225	Angiosperms	Zingiberaceae	*Alpinia galanga*	PS0515MT05
226	Angiosperms	Zingiberaceae	*Alpinia hainanensis*	PS0511MT01
227	Angiosperms	Zingiberaceae	*Alpinia zerumbet*	PS0532MT01
228	Ferns	Lygodiaceae	*Lygodium japonicum*	PS0395MT01

229	Ferns	Lygodiaceae	*Lygodium japonicum*	PS0395MT02
230	Ferns	Lygodiaceae	*Lygodium japonicum*	PS0395MT03
231	Ferns	Lygodiaceae	*Lygodium japonicum*	PS0395MT04
232	Ferns	Lygodiaceae	*Lygodium japonicum*	PS0395MT05
233	Ferns	Selaginellaceae	*Selaginella doederleinii*	PS0729MT01
234	Ferns	Selaginellaceae	*Selaginella moellendorffii*	PS0727MT01
235	Ferns	Selaginellaceae	*Selaginella uncinata*	PS0726MT01
236	Gymnosperms	Ephedraceae	*Ephedra equisetina*	PS0848MT01
237	Gymnosperms	Ephedraceae	*Ephedra equisetina*	PS0848MT02
238	Gymnosperms	Ephedraceae	*Ephedra sinica*	PS0847MT01
239	Gymnosperms	Pinaceae	*Pinus bungeana*	PS1348MT01
240	Gymnosperms	Pinaceae	*Pinus ponderosa*	PS1359MT01
241	Gymnosperms	Pinaceae	*Pinus strobus*	PS1350MT01
242	Gymnosperms	Pinaceae	*Pinus wallichiana*	PS1357MT01
243	Gymnosperms	Taxaceae	*Torreya californica*	PS1186MT01
244	Gymnosperms	Taxaceae	*Torreya fargesii* var. *yunnanensis*	PS1190MT01
245	Gymnosperms	Taxaceae	*Torreya grandis*	PS1744MT01
246	Gymnosperms	Taxaceae	*Torreya grandis*	PS1744MT02
247	Gymnosperms	Taxaceae	*Torreya nucifera*	PS1187MT01
