# Supplementary material for: Extensive Pyrosequencing Reveals Frequent Intra-Genomic Variations of Internal Transcribed Spacer Regions of Nuclear Ribosomal DNA
Source: PLoS One. 2012 Aug 30;7(8):e43971. doi: 10.1371/journal.pone.0043971 (PMC3431384; doi:10.1371/journal.pone.0043971)
Supplement: Table S5 — Sequence-tagged PCR primer pairs for each sample. (PDF) [file pone.0043971.s015.pdf]

**Table S5.** Sequence-tagged PCR primer pairs for each sample.

| No. | Species | Forward Primer (5'-3') | Reverse Primer (5'-3') | Type of Primer* | Voucher Number |
|---|---|---|---|---|---|
| 1 | *Acorus calamus* | TAGGCAAGCTGAGTCTTTGAAC GCAAGTTG | TAGGCAAGCTTCCTCCGCTTAT TGATATG | A | PS1418MT03 |
| 2 | *Acorus calamus* | TAGGCTTCAGGAGTCTTTGAAC GCAAGTTG | TAGGCTTCAGTCCTCCGCTTAT TGATATG | A | PS1418MT04 |
| 3 | *Acorus gramineus* | TACCGAAGTCGAGTCTTTGAAC GCAAGTTG | TACCGAAGTCTCCTCCGCTTAT TGATATG | A | PS1301MT04 |
| 4 | *Acorus gramineus* | TACGAGTATGGAGTCTTTGAAC GCAAGTTG | TACGAGTATGTCCTCCGCTTATT GATATG | A | PS1301MT05 |
| 5 | *Allium tuberosum* | CGTACACAGAATCCCGTGAATC ATCG | CGTACAGCCCCGTATGATCTGA GGTC | C | PS0038MT01 |
| 6 | *Allium tuberosum* | CTAGCACAGAATCCCGTGAATC ATCG | CTAGCAGCCCCGTATGATCTGA GGTC | C | PS0038MT02 |
| 7 | *Allium tuberosum* | TCAGGACAGAATCCCGTGAATC ATCG | TCAGGAGCCCCGTATGATCTGA GGTC | C | PS0038MT03 |
| 8 | *Allium tuberosum* | TGCACACAGAATCCCGTGAATC ATCG | TGCACAGCCCCGTATGATCTGA GGTC | C | PS0038MT05 |
| 9 | *Aloe vera* | ACGGTCCATGCGAGTTTTTGAA CGCAAGTTG | ACGGTCCATGGTTTCTTTTCCT CCGCTTATTG | B | PS0044MT01 |
| 10 | *Aloe vera* | AGACTCAGCGCGAGTTTTTGA ACGCAAGTTG | AGACTCAGCGGTTTCTTTTCCT CCGCTTATTG | B | PS0044MT02 |
| 11 | *Aloe vera* | AGAGCGTCACCGAGTTTTTGA ACGCAAGTTG | AGAGCGTCACGTTTCTTTTCCT CCGCTTATTG | B | PS0044MT03 |
| 12 | *Alpinia galanga* | AGTCCTGAATCATTGAGTCTTT GAACGC | AGTCCTTCCTCCTCCGCTTATTT ATATG | C | PS0515MT02 |

| | | | | | |
|---|---|---|---|---|---|
| 13 | *Alpinia galanga* | ATCGGTGAATCATTGAGTCTTTGAACGC | ATCGGTTCCTCCTCCGCTTATTTATATG | C | PS0515MT03 |
| 14 | *Alpinia galanga* | TCAGGCGAATCATTGAGTCTTTGAACGC | TCAGGCTCCTCCTCCGCTTATTTATATG | C | PS0515MT05 |
| 15 | *Alpinia hainanensis* | AGTCAGCGTGAACCATTGAGTCTTTG | AGTCAGGTTTCTCCTCCTCCGCTTAT | C | PS0511MT01 |
| 16 | *Alpinia zerumbet* | CGTGAACCATTGAGTCTTTGA | GTTTCTCCTCCTCCGCTTAT | C | PS0532MT01 |
| 17 | *Amygdalus persica* | TGACCATTCGGAGTCTTTGAACGCAAGTTG | TGACCATTCGTCCTCCGCTTATTGATATG | A | PS1117MT02 |
| 18 | *Amygdalus triloba* | TGACCTGGACGAGTCTTTGAACGCAAGTTG | TGACCTGGACTCCTCCGCTTATTGATATG | A | PS1127MT02 |
| 19 | *Angelica dahurica* | CTATAGCGTAGAGTCTTTGAACGCAAGTTG | CTATAGCGTATCCTCCGCTTATTGATATG | A | PS1197MT01 |
| 20 | *Angelica dahurica* | CTATGTACAGGAGTCTTTGAACGCAAGTTG | CTATGTACAGTCCTCCGCTTATTGATATG | A | PS1197MT03 |
| 21 | *Angelica decursiva* | TACACACACTGAGTCTTTGAACGCAAGTTG | TACACACACTTCCTCCGCTTATTGATATG | A | PS1226MT04 |
| 22 | *Angelica decursiva* | TACAGATCGTGAGTCTTTGAACGCAAGTTG | TACAGATCGTTCCTCCGCTTATTGATATG | A | PS1226MT05 |
| 23 | *Angelica sinensis* | CTCGATATAGGAGTCTTTGAACGCAAGTTG | CTCGATATAGTCCTCCGCTTATTGATATG | A | PS1205MT01 |
| 24 | *Arabidopsis thaliana* | ACGAGTGCGTGCAGAATCCCGTGAACCA | ACGAGTGCGTTCTTTTCCTCCGCTTATTGAT | C | PS9001MT01 |
| 25 | *Arabidopsis thaliana* | TGCTCGCTACGCAGAATCCCGTGAACCA | TGCTCGCTACTCTTTTCCTCCGCTTATTGAT | C | PS9001MT02 |
| 26 | *Arabidopsis thaliana* | TCGCAGACACGCAGAATCCCGTGAACCA | TCGCAGACACTCTTTTCCTCCGCTTATTGAT | C | PS9001MT03 |
| 27 | *Ardisia crenata* | ATAGATAGACCGAGTTTTTGAA | ATAGATAGACGTTTCTTTTCCTC | B | PS1658MT02 |

| | | | | | |
|---|---|---|---|---|---|
| | | CGCAAGTTG | CGCTTATTG | | |
| 28 | *Ardisia crenata* | CTCTACGCTCCGAGTTTTTGAA CGCAAGTTG | CTCTACGCTCGTTTCTTTTCCTC CGCTTATTG | B | PS1658MT03 |
| 29 | *Ardisia japonica* | TGTCGTCGCACGAGTTTTTGAA CGCAAGTT | TGTCGTCGCAGTTTCTTTTCCT CCGCTTATT | C | PS1659MT05 |
| 30 | *Armeniaca sibirica* | CGATTGGAATCCCGTGAACCAT CG | CGATTGAGTTTCTTTTCCTCCG CTTA | C | PS1121MT04 |
| 31 | *Armeniaca vulgaris* | TCTGATCGAGGAGTCTTTGAAC GCAAGTTG | TCTGATCGAGTCCTCCGCTTATT GATATG | A | PS1123MT01 |
| 32 | *Armeniaca vulgaris* | TGAACGGCATGAGTCTTTGAAC GCAAGTTG | TGAACGGCATTCCTCCGCTTAT TGATATG | A | PS1123MT02 |
| 33 | *Artemisia annua* | ATAGCTCTCGCGAGTTTTTGAA CGCAAGTTG | ATAGCTCTCGGTTTCTTTTCCTC CGCTTATTG | B | PS0633MT04 |
| 34 | *Artemisia annua* | CGCGTGCTAGCGAGTTTTTGAA CGCAAGTTG | CGCGTGCTAGGTTTCTTTTCCT CCGCTTATTG | B | PS0633MT05 |
| 35 | *Artemisia argyi* | ATCGAATCCCGTGAACCATCG | GCACCTTGTAAGTTTCTTTTCC TCC | C | PS0590MT04 |
| 36 | *Artemisia capillaris* | TTTCAGAATCCCGTGAACCAT | GCCTTTTCCTCCGCTTATTGA | C | PS0712MT02 |
| 37 | *Artemisia gmelinii* | ACGCAGAATCCCGTGAACCAT | GCGTTTCTTTTCCTCCGCTTA | C | PS0594MT01 |
| 38 | *Artemisia lavandulaefolia* | TTGAATCCCGTGAACCATCG | CGACCTTGTAAGTTTCTTTTCC TCC | C | PS0703MT01 |
| 39 | *Asparagus cochinchinensis* | ACGTTCATTCCCGTGAACCCAT CG | ACGTTCTTCTTCTCCTCCGCTTA TTGA | C | PS0057MT01 |
| 40 | *Asparagus cochinchinensis* | ACGTTGATTCCCGTGAACCCAT CG | ACGTTGTTCTTCTCCTCCGCTTA TTGA | C | PS0057MT04 |
| 41 | *Asparagus cochinchinensis* | ACTGACATTCCCGTGAACCCAT CG | ACTGACTTCTTCTCCTCCGCTTA TTGA | C | PS0057MT05 |
| 42 | *Asparagus* | AGCTAGATTCCCGTGAACCCAT | AGCTAGTTCTTCTCCTCCGCTTA | C | PS0057MT06 |

| | | | | | |
|---|---|---|---|---|---|
| | *cochinchinensis* | CG | TTGA | | |
| 43 | *Asparagus schoberioides* | CGTATGCGACGAGTCTTTGAAC GCAAGTTG | CGTATGCGACTCCTCCGCTTATT GATATG | A | PS0058MT01 |
| 44 | *Asparagus trichophyllus* | CGTTATTACGGAGTCTTTGAAC GCAAGTTG | CGTTATTACGTCCTCCGCTTATT GATATG | A | PS0059MT01 |
| 45 | *Asparagus trichophyllus* | CTACGACTGCGAGTCTTTGAAC GCAAGTTG | CTACGACTGCTCCTCCGCTTAT TGATATG | A | PS0059MT02 |
| 46 | *Aster ageratoides* | TACAGAATCCCGTGAACCATC | GCTTGTAAGTTTCTTTTCCTCC G | C | PS0680MT01 |
| 47 | *Aster tataricus* | ATACGACGTACGAGTTTTTGAA CGCAAGTTG | ATACGACGTAGTTTCTTTTCCTC CGCTTATTG | B | PS0721MT03 |
| 48 | *Astragalus chinensis* | CGAGAGATACGAGTCTTTGAAC GCAAGTTG | CGAGAGATACTCCTCCGCTTAT TGATATG | A | PS0272MT01 |
| 49 | *Astragalus hancockii* | CACACGATAGGAGTCTTTGAAC GCAAGTTG | CACACGATAGTCCTCCGCTTAT TGATATG | A | PS0271MT01 |
| 50 | *Astragalus mongholicus* | TGCTAGTCAGGAGTCTTTGAAC GCAAGTTG | TGCTAGTCAGTCCTCCGCTTAT TGATATG | A | PS0277MT01 |
| 51 | *Boehmeria nivea* | AGCGACTAGCGAGTCTTTGAA CGCAAGTTG | AGCGACTAGCTCCTCCGCTTAT TGATATG | A | PS1031MT01 |
| 52 | *Boehmeria nivea* | ATCCGCCTAGGAGTCTTTGAAC GCAAGTTG | ATCCGCCTAGTCCTCCGCTTATT GATATG | A | PS1031MT05 |
| 53 | *Brucea javanica* | AGCTCACGTAGAGTCTTTGAAC GCAAGTTG | AGCTCACGTATCCTCCGCTTATT GATATG | A | PS0753MT01 |
| 54 | *Brucea javanica* | AGTCGAGAGAGAGTCTTTGAA CGCAAGTTG | AGTCGAGAGATCCTCCGCTTAT TGATATG | A | PS0753MT02 |
| 55 | *Brucea javanica* | AGTGCTACGAGAGTCTTTGAAC GCAAGTTG | AGTGCTACGATCCTCCGCTTAT TGATATG | A | PS0753MT05 |
| 56 | *Brucea mollis* | CAGCAGAATCCCGTGAACCAT | GGTTCTTTTCCTCCGCTTATTG | C | PS0752MT01 |

| | | | | | |
|---|---|---|---|---|---|
| 57 | *Celosia argentea* | TATGCTAGTACGAGTTTTTGAA CGCAAGTTG | TATGCTAGTAGTTTCTTTTCCTC CGCTTATTG | B | PS1495MT02 |
| 58 | *Celosia cristata* | ACAGTATATACGAGTTTTTGAA CGCAAGTTG | ACAGTATATAGTTTCTTTTCCTC CGCTTATTG | B | PS1491MT02 |
| 59 | *Cerasus glandulosa* | TCGAATCCGAGAGTCTTTGAAC GCAAGTTG | TCGAATCCGATCCTCCGCTTATT GATATG | A | PS1088MT01 |
| 60 | *Cerasus japonica* | CGTAACCAGAATCCCGTGAACC ATC | CGTAACTCTTTTCCTCCGCTTAT TGA | C | PS1128MT01 |
| 61 | *Cerasus tomentosa* | TCTATACTATGAGTCTTTGAACG CAAGTTG | TCTATACTATTCCTCCGCTTATT GATATG | A | PS1092MT01 |
| 62 | *Cimicifuga dahurica* | AGCCTGGCTAGAGTCTTTGAAC GCAAGTTG | AGCCTGGCTATCCTCCGCTTATT GATATG | A | PS0929MT02 |
| 63 | *Cimicifuga foetida* | CGACACTATCGAGTCTTTGAAC GCAAGTTG | CGACACTATCTCCTCCGCTTATT GATATG | A | PS0925MT01 |
| 64 | *Cimicifuga foetida* | CTAGTCACTCGAGTCTTTGAAC GCAAGTTG | CTAGTCACTCTCCTCCGCTTATT GATATG | A | PS0925MT02 |
| 65 | *Cirsium japonicum* | CTGGACCTGACGAGTTTTTGAA CGCAAGTT | CTGGACCTGAGTTTCTTTTCCT CCGCTTATT | C | PS0612MT02 |
| 66 | *Cirsium japonicum* | CACGCTACGTCGAGTTTTTGAA CGCAAGTT | CACGCTACGTGTTTCTTTTCCT CCGCTTATT | C | PS0612MT03 |
| 67 | *Cirsium japonicum* | TCTCTATGCGCGAGTTTTTGAA CGCAAGTTG | TCTCTATGCGGTTTCTTTTCCTC CGCTTATTG | B | PS0612MT06 |
| 68 | *Cirsium setosum* | CAGGTCCAGTCGAGTTTTTGAA CGCAAGTTG | CAGGTCCAGTGTTTCTTTTCCT CCGCTTATTG | B | PS0611MT01 |
| 69 | *Cirsium setosum* | TCGTGACATGCGAGTTTTTGAA CGCAAGTTG | TCGTGACATGGTTTCTTTTCCT CCGCTTATTG | B | PS0611MT02 |
| 70 | *Cirsium setosum* | ATCCGCCGTACGAGTTTTTGAA CGCAAGTT | ATCCGCCGTAGTTTCTTTTCCTC CGCTTATT | C | PS0611MT04 |

| | | | | | |
|---|---|---|---|---|---|
| 71 | *Citrus aurantium* | ACTGGACCTGGAGTCTTTGAACGCAAGTTG | ACTGGACCTGTCCTCCGCTTATTGATATG | A | PS1613MT01 |
| 72 | *Citrus limonum* | ACGTTACCGTGAGTCTTTGAACGCAAGTTG | ACGTTACCGTTCCTCCGCTTATTGATATG | A | PS1609MT01 |
| 73 | *Citrus maxima* | ACGGTGGATCGAGTCTTTGAACGCAAGTTG | ACGGTGGATCTCCTCCGCTTATTGATATG | A | PS1600MT04 |
| 74 | *Citrus maxima* | AGACGCACTCGAGTCTTTGAACGCAAGTTG | AGACGCACTCTCCTCCGCTTATTGATATG | A | PS1600MT05 |
| 75 | *Citrus maxima* | AGCCTCCAGTGAGTCTTTGAACGCAAGTTG | AGCCTCCAGTTCCTCCGCTTATTGATATG | A | PS1600MT06 |
| 76 | *Citrus maxima* | AGCCTCCTGAGAGTCTTTGAACGCAAGTTG | AGCCTCCTGATCCTCCGCTTATTGATATG | A | PS1600MT08 |
| 77 | *Citrus maxima* | AGCCTGGCATGAGTCTTTGAACGCAAGTTG | AGCCTGGCATTCCTCCGCTTATTGATATG | A | PS1600MT09 |
| 78 | *Citrus medica* | AGCTTAGGCTGAGTCTTTGAACGCAAGTTG | AGCTTAGGCTTCCTCCGCTTATTGATATG | A | PS1616MT02 |
| 79 | *Citrus reticulata* | ATCGGAGAATCCCGTGACCCATC | ATCGGATCTTTCCTCCGCTTATTG | C | PS1596MT01 |
| 80 | *Clerodendrum bungei* | ACATGACGACGAGTCTTTGAACGCAAGTTG | ACATGACGACTCCTCCGCTTATTGATATG | A | PS0853MT01 |
| 81 | *Clerodendrum cytophyllum* | TAGCCAGGCTGAGTCTTTGAACGCAAGTTG | TAGCCAGGCTTCCTCCGCTTATTGATATG | A | PS0856MT02 |
| 82 | *Clerodendrum japonicum* | ACACATACGCGAGTCTTTGAACGCAAGTTG | ACACATACGCTCCTCCGCTTATTGATATG | A | PS0851MT01 |
| 83 | *Cryptotaenia japonica* | CGTAGACTAGGAGTCTTTGAACGCAAGTTG | CGTAGACTAGTCCTCCGCTTATTGATATG | A | PS1223MT01 |
| 84 | *Cynanchum atratum* | ATGGAGTCTTTGAACGCA | GGGGAATCCTTGTTAGTTT | C | PS0833MT01 |
| 85 | *Cynanchum* | TGTCACACGAGAGTCTTTGAA | TGTCACACGATCCTCCGCTTAT | A | PS0836MT01 |

| | | | | | |
|---|---|---|---|---|---|
| | *paniculatum* | CGCAAGTTG | TGATATG | | |
| 86 | *Cynanchum stauntonii* | CGTGTCTCTAGAGTCTTTGAACGCAAGTTG | CGTGTCTCTATCCTCCGCTTATTGATATG | A | PS0835MT04 |
| 87 | *Datura arborea* | CGTTACCGTAGAGTCTTTGAACGCAAGTTG | CGTTACCGTATCCTCCGCTTATTGATATG | A | PS1147MT01 |
| 88 | *Datura metel* | CGTTAGGTACGAGTCTTTGAACGCAAGTTG | CGTTAGGTACTCCTCCGCTTATTGATATG | A | PS1152MT02 |
| 89 | *Daucus carota* | ACGGTAACGTGAGTCTTTGAACGCAAGTTG | ACGGTAACGTTCCTCCGCTTATTGATATG | A | PS1225MT02 |
| 90 | *Dendrobium capillipes* | ATCGGCAAGTGAGTCTTTGAACGCAAGTTG | ATCGGCAAGTTCCTCCGCTTATTGATATG | A | PS2502MT01 |
| 91 | *Dendrobium catenatum* | CGAATAACTGGAGTCTTTGAACGCAAGTTG | CGAATAACTGTCCTCCGCTTATTGATATG | A | PS2521MT01 |
| 92 | *Dendrobium chrysanthum* | CAGTTAGGTCGAGTCTTTGAACGCAAGTTG | CAGTTAGGTCTCCTCCGCTTATTGATATG | A | PS2515MT01 |
| 93 | *Dendrobium chrysotoxum* | ATAGAGTACTGAGTCTTTGAACGCAAGTTG | ATAGAGTACTTCCTCCGCTTATTGATATG | A | PS2501MT01 |
| 94 | *Dendrobium chrysotoxum* | ATCGGATTCGGAGTCTTTGAACGCAAGTTG | ATCGGATTCGTCCTCCGCTTATTGATATG | A | PS2501MT03 |
| 95 | *Dendrobium crepidatum* | CAGTTCAAGTGAGTCTTTGAACGCAAGTTG | CAGTTCAAGTTCCTCCGCTTATTGATATG | A | PS2517MT01 |
| 96 | *Dendrobium crystallinum* | CATGGCAATGGAGTCTTTGAACGCAAGTTG | CATGGCAATGTCCTCCGCTTATTGATATG | A | PS2519MT01 |
| 97 | *Dendrobium cucullatum* | CGACAGCGAGGAGTCTTTGAACGCAAGTTG | CGACAGCGAGTCCTCCGCTTATTGATATG | A | PS2523MT01 |
| 98 | *Dendrobium denneanum* | ATGGCTTACGGAGTCTTTGAACGCAAGTTG | ATGGCTTACGTCCTCCGCTTATTGATATG | A | PS0758MT01 |
| 99 | *Dendrobium* | CATTGAAGCTGAGTCTTTGAAC | CATTGAAGCTTCCTCCGCTTATT | A | PS2520MT01 |

| | | | | | |
|---|---|---|---|---|---|
| | *devonianum* | GCAAGTTG | GATATG | | |
| 100 | *Dendrobium gratiosissimum* | CAGGTGGCTAGAGTCTTTGAAC GCAAGTTG | CAGGTGGCTATCCTCCGCTTAT TGATATG | A | PS2513MT01 |
| 101 | *Dendrobium loddigesii* | AGTCCTCCAGGAGTCTTTGAAC GCAAGTTG | AGTCCTCCAGTCCTCCGCTTAT TGATATG | A | PS1748MT02 |
| 102 | *Dendrobium nobile* | ATGTACGATGGAGTCTTTGAAC GCAAGTTG | ATGTACGATGTCCTCCGCTTATT GATATG | A | PS0766MT04 |
| 103 | *Dendrobium nobile* | ATGTGTCTAGGAGTCTTTGAAC GCAAGTTG | ATGTGTCTAGTCCTCCGCTTATT GATATG | A | PS0766MT06 |
| 104 | *Dendrobium pendulum* | CAGGTGGCATGAGTCTTTGAAC GCAAGTTG | CAGGTGGCATTCCTCCGCTTAT TGATATG | A | PS2511MT01 |
| 105 | *Dendrobium polyanthum* | CAGTTGCCTAGAGTCTTTGAAC GCAAGTTG | CAGTTGCCTATCCTCCGCTTATT GATATG | A | PS2518MT01 |
| 106 | *Dendrobium polyanthum* | CATGGAGGCTGAGTCTTTGAAC GCAAGTTG | CATGGAGGCTTCCTCCGCTTAT TGATATG | A | PS2518MT02 |
| 107 | *Dendrobium spatella* | CGATTACCTGGAGTCTTTGAAC GCAAGTTG | CGATTACCTGTCCTCCGCTTATT GATATG | A | PS2527MT01 |
| 108 | *Dendrobium trigonopus* | ATGCCGTTACGAGTCTTTGAAC GCAAGTTG | ATGCCGTTACTCCTCCGCTTATT GATATG | A | PS2506MT01 |
| 109 | *Dendrobium wardianum* | ATGCCTAACGGAGTCTTTGAAC GCAAGTTG | ATGCCTAACGTCCTCCGCTTATT GATATG | A | PS2509MT01 |
| 110 | *Dendrobium wardianum* | ATGGCGGTCAGAGTCTTTGAAC GCAAGTTG | ATGGCGGTCATCCTCCGCTTATT GATATG | A | PS2509MT02 |
| 111 | *Dendrobium williamsonii* | ATGCCATTGCGAGTCTTTGAAC GCAAGTTG | ATGCCATTGCTCCTCCGCTTATT GATATG | A | PS2503MT01 |
| 112 | *Dichroa febrifuga* | CGATTCGGATGAGTCTTTGAAC GCAAGTTG | CGATTCGGATTCCTCCGCTTATT GATATG | A | PS0850MT01 |
| 113 | *Eleutherococcus* | TATCACTCAGGAGTCTTTGAAC | TATCACTCAGTCCTCCGCTTATT | A | PS1461MT01 |

| | | | | | |
|---|---|---|---|---|---|
| | *giraldii* | GCAAGTTG | GATATG | | |
| 114 | *Eleutherococcus* | TCACGTACTAGAGTCTTTGAAC | TCACGTACTATCCTCCGCTTATT | A | PS1473MT01 |
| | *nodiflorus* | GCAAGTTG | GATATG | | |
| 115 | *Eleutherococcus* | TAGTCGCATAGAGTCTTTGAAC | TAGTCGCATATCCTCCGCTTATT | A | PS1456MT03 |
| | *senticosus* | GCAAGTTG | GATATG | | |
| 116 | *Ephedra equisetina* | ATGCACCGAGTCTTTGAACGCA | ATGCACTATGCTTAAACTCAGC | A | PS0848MT01 |
| | | AGTTG | GGGTG | | |
| 117 | *Ephedra equisetina* | CAGTTCCGAGTCTTTGAACGCA | CAGTTCTATGCTTAAACTCAGC | A | PS0848MT02 |
| | | AGTTG | GGGTG | | |
| 118 | *Ephedra sinica* | CAGTAGACGTGAGTCTTTGAAC | CAGTAGACGTTCCTCCGCTTAT | A | PS0847MT01 |
| | | GCAAGTTG | TGATATG | | |
| 119 | *Epimedium* | TGATACGTCTGAGTCTTTGAAC | TGATACGTCTTCCTCCGCTTATT | A | PS1499MT01 |
| | *acuminatum* | GCAAGTTG | GATATG | | |
| 120 | *Epimedium* | TGATGTGTACGAGTCTTTGAAC | TGATGTGTACTCCTCCGCTTATT | A | PS1499MT02 |
| | *acuminatum* | GCAAGTTG | GATATG | | |
| 121 | *Epimedium pubescens* | TGTAGTGTGAGAGTCTTTGAAC | TGTAGTGTGATCCTCCGCTTATT | A | PS1505MT01 |
| | | GCAAGTTG | GATATG | | |
| 122 | *Epimedium* | TGCTATAGACGAGTCTTTGAAC | TGCTATAGACTCCTCCGCTTATT | A | PS1502MT02 |
| | *sagittatum* | GCAAGTTG | GATATG | | |
| 123 | *Epimedium* | TGTACTACTCGAGTCTTTGAAC | TGTACTACTCTCCTCCGCTTATT | A | PS1502MT03 |
| | *sagittatum* | GCAAGTTG | GATATG | | |
| 124 | *Eupatorium fortunei* | AGACTATACTCGAGTTTTTGAA | AGACTATACTGTTTCTTTTCCTC | B | PS0672MT01[$] |
| | | CGCAAGTTG | CGCTTATTG | | |
| 125 | *Euphorbia esula* | ATCAGACACGGAGTCTTTGAA | ATCAGACACGTCCTCCGCTTAT | A | PS0193MT01 |
| | | CGCAAGTTG | TGATATG | | |
| 126 | *Euphorbia hirta* | AGTTCAAGTCGAGTCTTTGAAC | AGTTCAAGTCTCCTCCGCTTAT | A | PS0175MT02 |
| | | GCAAGTTG | TGATATG | | |
| 127 | *Euphorbia hirta* | CTAGGTCCAGGAGTCTTTGAAC | CTAGGTCCAGTCCTCCGCTTAT | A | PS0175MT03 |

| | | | | | |
|---|---|---|---|---|---|
| | | GCAAGTTG | TGATATG | | |
| 128 | *Euphorbia pekinensis* | CTAGGCTTAGGAGTCTTTGAAC GCAAGTTG | CTAGGCTTAGTCCTCCGCTTATT GATATG | A | PS0187MT01 |
| 129 | *Flemingia lineata* | ATACAGAATCCCGTGAACCAT | CGCTTTTCCTCCGCTTATTGA | C | PS0223MT01 |
| 130 | *Flemingia macrophylla* | AGAGAGTGTGGAGTCTTTGAA CGCAAGTTG | AGAGAGTGTGTCCTCCGCTTAT TGATATG | A | PS0222MT02 |
| 131 | *Flemingia macrophylla* | AGTCTGACTGGAGTCTTTGAAC GCAAGTTG | AGTCTGACTGTCCTCCGCTTAT TGATATG | A | PS0222MT03 |
| 132 | *Foeniculum vulgare* | TACGGTAACGGAGTCTTTGAAC GCAAGTTG | TACGGTAACGTCCTCCGCTTAT TGATATG | A | PS1222MT03 |
| 133 | *Gentiana macrophylla* | AAGAATCCCGTGAACCATCG | GGTCCTTGTTAGTTTCTTTTCCT CC | C | PS0821MT02 |
| 134 | *Gentiana manshurica* | GACCAGAATCCCGTGAACCAT | CCTTCTTTTCCTCCGCTTATTG | C | PS0823MT01 |
| 135 | *Gentiana rigescens* | CAGGTAAGTCGAGTCTTTGAAC GCAAGTTG | CAGGTAAGTCTCCTCCGCTTAT TGATATG | A | PS0822MT01 |
| 136 | *Gentiana straminea* | CGTACTCAGAGAGTCTTTGAAC GCAAGTTG | CGTACTCAGATCCTCCGCTTATT GATATG | A | PS0820MT01 |
| 137 | *Gentiana straminea* | CTACGCTCTAGAGTCTTTGAAC GCAAGTTG | CTACGCTCTATCCTCCGCTTATT GATATG | A | PS0820MT02 |
| 138 | *Ilex asprella* | TGCCAGGTCACGAGTTTTTGAA CGCAAGTT | TGCCAGGTCAGTTTCTTTTCCT CCGCTTATT | C | PS0325MT01 |
| 139 | *Ilex cornuta* | CGATCTGTCGCGAGTTTTTGAA CGCAAGTT | CGATCTGTCGGTTTCTTTTCCTC CGCTTATT | C | PS0320MT01 |
| 140 | *Ilex cornuta* | CGCAGTACGACGAGTTTTTGAA CGCAAGTT | CGCAGTACGAGTTTCTTTTCCT CCGCTTATT | C | PS0320MT03 |
| 141 | *Ilex cornuta* | CGTCGATCTCCGAGTTTTTGAA CGCAAGTT | CGTCGATCTCGTTTCTTTTCCTC CGCTTATT | C | PS0320MT05 |
| 142 | *Ilex pubilimba* | TGCCAGGACTCGAGTTTTTGAA | TGCCAGGACTGTTTCTTTTCCT | C | PS0324MT01 |

| | | | | | |
|---|---|---|---|---|---|
| | | CGCAAGTT | CCGCTTATT | | |
| 143 | *Ilex rotunda* | TGCCACCGATCGAGTTTTTGAA CGCAAGTT | TGCCACCGATGTTTCTTTTCCT CCGCTTATT | C | PS0321MT01 |
| 144 | *Inula britanica* | TATACATGTGCGAGTTTTTGAA CGCAAGTTG | TATACATGTGGTTTCTTTTCCTC CGCTTATTG | B | PS0670MT01 |
| 145 | *Inula britanica* | TATCTGATAGCGAGTTTTTGAA CGCAAGTTG | TATCTGATAGGTTTCTTTTCCTC CGCTTATTG | B | PS0670MT02 |
| 146 | *Inula cappa* | GCACAGAATCCCGTGAACCAT | CGATCTTTTCCTCCGCTTATTG | C | PS0701MT01 |
| 147 | *Inula helenium* | AGCCAGAATCCCGTGAACCAT | GCATCTTTTCCTCCGCTTATTG | C | PS0689MT01 |
| 148 | *Ipomoea nil* | TGTATCACAGGAGTCTTTGAAC GCAAGTTG | TGTATCACAGTCCTCCGCTTATT GATATG | A | PS1536MT01 |
| 149 | *Ipomoea nil* | TGTGAGTAGTGAGTCTTTGAAC GCAAGTTG | TGTGAGTAGTTCCTCCGCTTAT TGATATG | A | PS1536MT05 |
| 150 | *Ipomoea purpurea* | ACAGTCGTGCGAGTCTTTGAA CGCAAGTTG | ACAGTCGTGCTCCTCCGCTTAT TGATATG | A | PS1542MT05 |
| 151 | *Ligusticum jeholense* | CTGTACATACGAGTCTTTGAAC GCAAGTTG | CTGTACATACTCCTCCGCTTATT GATATG | A | PS1213MT02 |
| 152 | *Ligusticum sinense* | CTGGATTGACGAGTCTTTGAAC GCAAGTTG | CTGGATTGACTCCTCCGCTTATT GATATG | A | PS1208MT01 |
| 153 | *Ligusticum sinense* cv. *Chuanxiong* | CTGAACCAGAATCCCGTGAAC CATC | CTGAACTCTTTTCCTCCGCTTAT TGA | C | PS1203MT01 |
| 154 | *Ligusticum sinense* cv. *Chuanxiong* | CTGACTCAGAATCCCGTGAACC ATC | CTGACTTCTTTTCCTCCGCTTAT TGA | C | PS1203MT02 |
| 155 | *Lilium pumilum* | ATATAGTCGCGAGTCTTTGAAC GCAAGTTG | ATATAGTCGCTCCTCCGCTTATT GATATG | A | PS0064MT01 |
| 156 | *Lilium tigrinum* | AGTTCGGACTGAGTCTTTGAAC GCAAGTTG | AGTTCGGACTTCCTCCGCTTAT TGATATG | A | PS0039MT01 |
| 157 | *Liriope spicata* | AGTTCTTGACGAGTCTTTGAAC | AGTTCTTGACTCCTCCGCTTATT | A | PS0055MT01 |

| | | | | | |
|---|---|---|---|---|---|
| | | GCAAGTTG | GATATG | | |
| 158 | *Lonicera confusa* | CGTTAGGTCAGAGTCTTTGAACGCAAGTTG | CGTTAGGTCATCCTCCGCTTATTGATATG | A | PS1161MT03 |
| 159 | *Lonicera japonica* | CTAAGCCTAGGAGTCTTTGAACGCAAGTTG | CTAAGCCTAGTCCTCCGCTTATTGATATG | A | PS1165MT02 |
| 160 | *Lygodium japonicum* | AGCTATCGAGTCTTTGAACGCAACTTG | AGCTATTGCCTGATTTGAGGTCCGAG | C | PS0395MT01 |
| 161 | *Lygodium japonicum* | AGCTTACGAGTCTTTGAACGCAACTTG | AGCTTATGCCTGATTTGAGGTCCGAG | C | PS0395MT02 |
| 162 | *Lygodium japonicum* | ATCGTACGAGTCTTTGAACGCAACTTG | ATCGTATGCCTGATTTGAGGTCCGAG | C | PS0395MT03 |
| 163 | *Lygodium japonicum* | CGTATACGAGTCTTTGAACGCAACTTG | CGTATATGCCTGATTTGAGGTCCGAG | C | PS0395MT04 |
| 164 | *Lygodium japonicum* | TCGATACGAGTCTTTGAACGCAACTTG | TCGATATGCCTGATTTGAGGTCCGAG | C | PS0395MT05 |
| 165 | *Melicope pteleifolia* | ACGTTGAATCGAGTCTTTGAACGCAAGTTG | ACGTTGAATCTCCTCCGCTTATTGATATG | A | PS1610MT01 |
| 166 | *Oryza sa tiva* ssp. *indica* | TACGTCGAGTCTTTGAACGCAAGTTG | TACGTCCCTCGTAAGTTTCTTCTCCTC | A | PS9003MT01 |
| 167 | *Oryza sa tiva* ssp. *indica* | TCGAGAGAGTCTTTGAACGCAAGTTG | TCGAGACCTCGTAAGTTTCTTCTCCTC | A | PS9003MT02 |
| 168 | *Oryza sa tiva* ssp. *indica* | TGCACTGAGTCTTTGAACGCAAGTTG | TGCACTCCTCGTAAGTTTCTTCTCCTC | A | PS9003MT03 |
| 169 | *Oryza sa tiva* ssp. *japonica* | CTGAGAGAGTCTTTGAACGCAAGTTG | CTGAGACCTCGTAAGTTTCTTCTCCTC | A | PS9002MT01 |
| 170 | *Oryza sa tiva* ssp. *japonica* | AGCTGAGAGTCTTTGAACGCAAGTTG | AGCTGACCTCGTAAGTTTCTTCTCCTC | A | PS9002MT02 |
| 171 | *Paeonia anomala* ssp. | ACGTGTATCACCGAGTCTTTGA | ACGTGTGTTTCTTTTCCTCCGC | C | PS0906MT01 |

| | | | | | |
|---|---|---|---|---|---|
| | *veitchii* | ACGC | TTATTG | | |
| 172 | *Paeonia anomala* ssp. *veitchii* | CAGTGTATCACCGAGTCTTTGA ACGC | CAGTGTGTTTCTTTTCCTCCGC TTATTG | C | PS0906MT02 |
| 173 | *Paeonia lactiflora* | ACGTCTCATCGAGTCTTTGAAC GCAAGTTG | ACGTCTCATCTCCTCCGCTTATT GATATG | A | PS0905MT01[&] |
| 174 | *Paeonia ostii* | AGTGACACACGAGTCTTTGAA CGCAAGTTG | AGTGACACACTCCTCCGCTTAT TGATATG | A | PS0913MT02 |
| 175 | *Panax ginseng* | TATGATACGCGAGTCTTTGAAC GCAAGTTG | TATGATACGCTCCTCCGCTTATT GATATG | A | PS1467MT01 |
| 176 | *Panax japonicus* | TCACTCATACGAGTCTTTGAAC GCAAGTTG | TCACTCATACTCCTCCGCTTATT GATATG | A | PS1477MT01 |
| 177 | *Panax japonicus* | TCGATAGTGAGAGTCTTTGAAC GCAAGTTG | TCGATAGTGATCCTCCGCTTATT GATATG | A | PS1477MT02 |
| 178 | *Panax japonicus* | TGACGTATGTGAGTCTTTGAAC GCAAGTTG | TGACGTATGTTCCTCCGCTTATT GATATG | A | PS1477MT03 |
| 179 | *Panax japonicus* | TGAGTCAGTAGAGTCTTTGAAC GCAAGTTG | TGAGTCAGTATCCTCCGCTTATT GATATG | A | PS1477MT04 |
| 180 | *Panax notoginseng* | TCAAGCCAGTGAGTCTTTGAA CGCAAGTTG | TCAAGCCAGTTCCTCCGCTTAT TGATATG | A | PS1469MT02 |
| 181 | *Panax notoginseng* | TCAAGCCTGAGAGTCTTTGAA CGCAAGTTG | TCAAGCCTGATCCTCCGCTTAT TGATATG | A | PS1469MT03 |
| 182 | *Panax quinquefolius* | TCAAGTTAGCGAGTCTTTGAAC GCAAGTTG | TCAAGTTAGCTCCTCCGCTTAT TGATATG | A | PS1472MT01 |
| 183 | *Periploca sepium* | TGCAACGGATGAGTCTTTGAAC GCAAGTTG | TGCAACGGATTCCTCCGCTTAT TGATATG | A | PS0840MT03 |
| 184 | *Pinus bungeana* | ACTGATCCGTGAATCATCCGAG TTTTTG | ACTGATAGCGGGTGTTCTCGCC TGA | C | PS1348MT01 |
| 185 | *Pinus ponderosa* | CAGTTAGCAGAATCCCGTGAAT | CAGTTAGGGTGTTCTCGCCTGA | C | PS1359MT01 |

| | | | | | |
|---|---|---|---|---|---|
| | | CATC | GC | | |
| 186 | *Pinus strobus* | CGATATGCAGAATCCCGTGAATCATC | CGATATGGGTGTTCTCGCCTGAGC | C | PS1350MT01 |
| 187 | *Pinus wallichiana* | TGACATGCAGAATCCCGTGAATCATC | TGACATGGGTGTTCTCGCCTGAGC | C | PS1357MT01 |
| 188 | *Piper longum* | ACGACTACAGGAGTCTTTGAACGCAAGTTG | ACGACTACAGTCCTCCGCTTATTGATATG | A | PS0445MT01 |
| 189 | *Piper longum* | TACTCTCGTGGAGTCTTTGAACGCAAGTTG | TACTCTCGTGTCCTCCGCTTATTGATATG | A | PS0445MT02 |
| 190 | *Piper nigrum* | ATCCGTTCGAGAGTCTTTGAACGCAAGTTG | ATCCGTTCGATCCTCCGCTTATTGATATG | A | PS0449MT02 |
| 191 | *Polygonum chinense* | CGCGTATACAGAGTCTTTGAACGCAAGTTG | CGCGTATACATCCTCCGCTTATTGATATG | A | PS0794MT01 |
| 192 | *Polygonum chinense* | CGTACAGTCAGAGTCTTTGAACGCAAGTTG | CGTACAGTCATCCTCCGCTTATTGATATG | A | PS0794MT02 |
| 193 | *Populus trichocarpa* | TCAGAGAGAATCCCGTGAACCATCG | TCAGAGTCCTCCGCTTATTGATATGCT | C | PS9004MT01 |
| 194 | *Potentilla chinensis* | CGACGTGACTCGAGTTTTTGAACGCAAGTTG | CGACGTGACTGTTTCTTTTCCTCCGCTTATTG | B | PS1120MT01 |
| 195 | *Potentilla chinensis* | TAGTGTAGATCGAGTTTTTGAACGCAAGTTG | TAGTGTAGATGTTTCTTTTCCTCCGCTTATTG | B | PS1120MT02 |
| 196 | *Potentilla fruticosa* | CATTGCCATGGAGTCTTTGAACGCAAGTTG | CATTGCCATGTCCTCCGCTTATTGATATG | A | PS1084MT01 |
| 197 | *Potentilla nivea* | TCGATCACGTCGAGTTTTTGAACGCAAGTTG | TCGATCACGTGTTTCTTTTCCTCCGCTTATTG | B | PS1124MT01 |
| 198 | *Potentilla supina* | TCAGGCTTGAGAGTCTTTGAACGCAAGTTG | TCAGGCTTGATCCTCCGCTTATTGATATG | A | PS1067MT01 |
| 199 | *Potentilla supina* | TCAGGTCCAGGAGTCTTTGAA | TCAGGTCCAGTCCTCCGCTTAT | A | PS1067MT02 |

| | | | | | |
|---|---|---|---|---|---|
| | | CGCAAGTTG | TGATATG | | |
| 200 | *Prenanthes tatarinowii* | CTAAGAACGTCGAGTTTTTGAACGCAAGTTG | CTAAGAACGTGTTTCTTTTCCTCCGCTTATTG | B | PS0671MT01 |
| 201 | *Pueraria montana* var. *lobata* | CGTCTAGTACGAGTCTTTGAACGCAAGTTG | CGTCTAGTACTCCTCCGCTTATTGATATG | A | PS0313MT04 |
| 202 | *Pueraria peduncularis* | AATCAGAATCCCGTGAACCAT | GGCTTTTCCTCCGCTTATTG | C | PS0314MT01 |
| 203 | *Rhus chinensis* | CATACTCTACGAGTCTTTGAACGCAAGTTG | CATACTCTACTCCTCCGCTTATTGATATG | A | PS1014MT03 |
| 204 | *Rosa bella* | TGAACAATCGGAGTCTTTGAACGCAAGTTG | TGAACAATCGTCCTCCGCTTATTGATATG | A | PS1098MT01 |
| 205 | *Rosa laevigata* | TCGGACCTAGGAGTCTTTGAACGCAAGTTG | TCGGACCTAGTCCTCCGCTTATTGATATG | A | PS1085MT01 |
| 206 | *Rubus chingii* | TGAGTGACGCGAGTCTTTGAACGCAAGTTG | TGAGTGACGCTCCTCCGCTTATTGATATG | A | PS1135MT01 |
| 207 | *Rubus parvifolius* | CATTGTTAGCGAGTCTTTGAACGCAAGTTG | CATTGTTAGCTCCTCCGCTTATTGATATG | A | PS1093MT01 |
| 208 | *Santalum album* | ACTTGTTCAGGAGTCTTTGAACGCAAGTTG | ACTTGTTCAGTCCTCCGCTTATTGATATG | A | PS1377MT03 |
| 209 | *Sedum emarginatum* | ATCTACTGACGAGTCTTTGAACGCAAGTTG | ATCTACTGACTCCTCCGCTTATTGATATG | A | PS0574MT03 |
| 210 | *Sedum lineare* | TCTAGCGACTGAGTCTTTGAACGCAAGTTG | TCTAGCGACTTCCTCCGCTTATTGATATG | A | PS0577MT01 |
| 211 | *Sedum sarmentosum* | AGCACTGTAGGAGTCTTTGAACGCAAGTTG | AGCACTGTAGTCCTCCGCTTATTGATATG | A | PS0575MT01 |
| 212 | *Sedum sarmentosum* | TACGCTGTCTGAGTCTTTGAACGCAAGTTG | TACGCTGTCTTCCTCCGCTTATTGATATG | A | PS0575MT02 |
| 213 | *Selaginella* | TGACCGTCCGTGAATCATCAAA | TGACCGTAGTTTCTTTTCCTCC | C | PS0729MT01 |

| | | | | | |
|---|---|---|---|---|---|
| | *doederleinii* | TGTT | GCTTA | | |
| 214 | *Selaginella moellendorffii* | AGCTCTCGAGTCTTTGAACGCA CATTG | AGCTCTTAGTTTCTTTTCCTCCG CTTAGT | C | PS0727MT01 |
| 215 | *Selaginella uncinata* | CGAATCTTTGAACGCACAT | CGGTTTCTTTTCCTCCGCTTA | C | PS0726MT01 |
| 216 | *Senna alata* | TAGCTCTATCGAGTCTTTGAAC GCAAGTTG | TAGCTCTATCTCCTCCGCTTATT GATATG | A | PS1362MT02 |
| 217 | *Senna obtusifolia* | ACGACAGCTCGAGTCTTTGAA CGCAAGTTG | ACGACAGCTCTCCTCCGCTTAT TGATATG | A | PS1588MT08 |
| 218 | *Siegesbeckia glabrescens* | TACCGCCATGGAGTCTTTGAAC GCAAGTTG | TACCGCCATGTCCTCCGCTTATT GATATG | A | PS0660MT01 |
| 219 | *Siegesbeckia orientalis* | ACGTGCAGCGCGAGTTTTTGA ACGCAAGTTG | ACGTGCAGCGGTTTCTTTTCCT CCGCTTATTG | B | PS0618MT05 |
| 220 | *Siraitia grosvenorii* | CTGAATGGACGAGTCTTTGAAC GCAAGTTG | CTGAATGGACTCCTCCGCTTAT TGATATG | A | PS0464MT02 |
| 221 | *Solanum lyratum* | CGAATCCGATGAGTCTTTGAAC GCAAGTTG | CGAATCCGATTCCTCCGCTTATT GATATG | A | PS1137MT01 |
| 222 | *Solanum nigrum* | AGTCCAGGTCGAGTCTTTGAA CGCAAGTTG | AGTCCAGGTCTCCTCCGCTTAT TGATATG | A | PS1144MT02 |
| 223 | *Sophora flavescens* | ATCGTCTGTGGAGTCTTTGAAC GCAAGTTG | ATCGTCTGTGTCCTCCGCTTATT GATATG | A | PS0247MT04 |
| 224 | *Sophora japonica* | ATCCGAATCGGAGTCTTTGAAC GCAAGTTG | ATCCGAATCGTCCTCCGCTTATT GATATG | A | PS0241MT03 |
| 225 | *Sophora tonkinensis* | ATCGTAGCAGGAGTCTTTGAAC GCAAGTTG | ATCGTAGCAGTCCTCCGCTTAT TGATATG | A | PS0228MT01 |
| 226 | *Stellaria vestita* | CGTAACGGTAGAGTCTTTGAAC GCAAGTTG | CGTAACGGTATCCTCCGCTTATT GATATG | A | PS1317MT01 |
| 227 | *Stemmacantha uniflora* | ACGTCTCATCGAGTCTTTGAAC GCAAGTTG | ACGTCTCATCTCCTCCGCTTATT GATATG | A | PS0674MT01[&] |

| | | | | | |
|---|---|---|---|---|---|
| 228 | *Tetradium ruticarpum* | ACTGGTCCGAGAGTCTTTGAACGCAAGTTG | ACTGGTCCGATCCTCCGCTTATTGATATG | A | PS1614MT01 |
| 229 | *Torreya californica* | CTAAGTTCAGGAGTCTTTGAACGCAAGTTG | CTAAGTTCAGTCCTCCGCTTATTGATATG | A | PS1186MT01 |
| 230 | *Torreya f argesii* var. *yunnanensis* | CTAGGTGAATCCCGTGAATCATCG | CTAGGTGTTTCTTTTCCTCCGCTTA | C | PS1190MT01 |
| 231 | *Torreya grandis* | TAGCCTGAGTCTTTGAACGCAAGTTG | TAGCCTCTTTTCCTCCGCTTAATGAT | A | PS1744MT01 |
| 232 | *Torreya grandis* | CGATTCGAGTCTTTGAACGCAAGTTG | CGATTCCTTTTCCTCCGCTTAATGAT | A | PS1744MT02 |
| 233 | *Torreya nucifera* | CTAAGTTGCAGAGTCTTTGAACGCAAGTTG | CTAAGTTGCATCCTCCGCTTATTGATATG | A | PS1187MT01 |
| 234 | *Uncaria macrophylla* | ATCCGTTAGCGAGTCTTTGAACGCAAGTTG | ATCCGTTAGCTCCTCCGCTTATTGATATG | A | PS1038MT01 |
| 235 | *Uncaria macrophylla* | ATGGCAATGCGAGTCTTTGAACGCAAGTTG | ATGGCAATGCTCCTCCGCTTATTGATATG | A | PS1038MT03 |
| 236 | *Uncaria macrophylla* | ATGGCGGTACGAGTCTTTGAACGCAAGTTG | ATGGCGGTACTCCTCCGCTTATTGATATG | A | PS1038MT04 |
| 237 | *Uncaria sessilifructus* | TCATCGAGTCGAGTCTTTGAACGCAAGTTG | TCATCGAGTCTCCTCCGCTTATTGATATG | A | PS1041MT02 |
| 238 | *Uncaria sinensis* | CATGCTCAGAATCCCGTGAACCATC | CATGCTGTAATCCCGCCTGACCTG | C | PS1039MT01 |
| 239 | *Verbena officinalis* | AGTACGCTATGAGTCTTTGAACGCAAGTTG | AGTACGCTATTCCTCCGCTTATTGATATG | A | PS0865MT03 |
| 240 | *Veronicastrum axillare* | AGCTTCTTGAGAGTCTTTGAACGCAAGTTG | AGCTTCTTGATCCTCCGCTTATTGATATG | A | PS1525MT01 |
| 241 | *Veronicastrum stenostachyum* | AGTCCGAACTGAGTCTTTGAACGCAAGTTG | AGTCCGAACTTCCTCCGCTTATTGATATG | A | PS1519MT01 |

| | | | | | |
|---|---|---|---|---|---|
| 242 | *Viola diffusa* | ACGCGAGTATGAGTCTTTGAACGCAAGTTG | ACGCGAGTATTCCTCCGCTTATTGATATG | A | PS0555MT01 |
| 243 | *Viola philippica* | ACTGTGCAGAATCCCGTGAATCATC | ACTGTGTCTTTTCCTCCGCTTATTG | C | PS0561MT02 |
| 244 | *Zanthoxylum bungeanum* | ACGCTCGACAGAGTCTTTGAACGCAAGTTG | ACGCTCGACATCCTCCGCTTATTGATATG | A | PS1599MT01 |
| 245 | *Zea mays* | TATAGACATCCGAGTTTTTGAACGCAAGT | TATAGACATCGTTTCTTCTCCTCCGCTTAT | C | PS9005MT01 |
| 246 | *Zea mays* | AGACTATACTCGAGTTTTTGAACGCAAGT | AGACTATACTGTTTCTTCTCCTCCGCTTAT | C | PS9005MT02[$] |
| 247 | *Zea mays* | AGTATACATACGAGTTTTTGAACGCAAGT | AGTATACATAGTTTCTTCTCCTCCGCTTAT | C | PS9005MT03 |

* A: universal primer type 1, B: universal primer type 2, C: special primer

[$] The 454 reads for the samples PS0672MT01 and PS9005MT02 were manually inspected because they have same primers and tags.

[&] The 454 reads for the samples PS0674MT01 and PS0905MT01 were manually inspected because they have same primers and tags.
