# Supplementary material for: Extensive Pyrosequencing Reveals Frequent Intra-Genomic Variations of Internal Transcribed Spacer Regions of Nuclear Ribosomal DNA
Source: PLoS One. 2012 Aug 30;7(8):e43971. doi: 10.1371/journal.pone.0043971 (PMC3431384; doi:10.1371/journal.pone.0043971)
Supplement: Table S6 — PCR cycling regime for primers in Table S5. (PDF) [file pone.0043971.s016.pdf]

Table S6. PCR cycling regime for primers in **Table S5**.

	Temperature	Time
1 cycle:	94 ℃	5min
33 cycles:	94 ℃	30sec
	56 ℃ (58 ℃) *	30sec
	72 ℃	40sec
1 cycle:	72 ℃	7min

* 56 ℃ for universal primer type 1 in **Table S5**

58 ℃ for special primers and universal primer type 2 in **Table S5**
