# Supplementary material for: Extensive Pyrosequencing Reveals Frequent Intra-Genomic Variations of Internal Transcribed Spacer Regions of Nuclear Ribosomal DNA
Source: PLoS One. 2012 Aug 30;7(8):e43971. doi: 10.1371/journal.pone.0043971 (PMC3431384; doi:10.1371/journal.pone.0043971)
Supplement: Table S7 — GenBank accession numbers of the 37 previously published ITS2 sequences from Araliaceae and two outgroup taxa. (PDF) [file pone.0043971.s017.pdf]

Table S7. GenBank accession numbers of the 37 previously published ITS2 sequences from Araliaceae and two outgroup taxa.

Species	Accession number
Aralia elata	AF273526
Aralia elata var. mandshurica	AF273528
Aralia spinosa	GU054699
Aralia stellata	GU054650
Billardiera heterophylla	AY829019
Brassaiopsis glomerulata	AY725111
Brassaiopsis grushvitzkyi	AF551728
Brassaiopsis malayana	DQ007367
Brassaiopsis sumatrana	DQ007365
Dendropanax dentiger	GU054694
Dendropanax hainanensis	AF242236
Eleutherococcus gracilistylus	GQ434779
Eleutherococcus japonicus f. kiusianus	AY548183
Eleutherococcus koreanus	AY548184
Eleutherococcus nodiflorus	U63184
Eleutherococcus senticosus	GU054610
Eleutherococcus senticosus f. inermis	AY548186
Eleutherococcus seoulensis	AY548187
Eleutherococcus sessiliflorus	GQ434778
Eleutherococcus sieboldianus	AY548190
Eleutherococcus simonii	AF242227
Eleutherococcus trifoliatus	AF551739
Eleutherococcus trifoliatus f. spinifolia	AY548191
Hymenosporum flavum	AY829026
Macropanax rosthornii	GU054613
Merrilliopanax alpinus	AY233309
Merrilliopanax chinensis	AY389040
Metapanax davidii	AF242233
Metapanax delavayi	GU054612
Oreopanax argentatus	DQ007400
Oreopanax echinops	AF242229
Panax japonicus	GQ434782
Panax notoginseng	AY271919
Panax pseudoginseng	U41693
Panax quinquefolius	U41688

Panax stipuleanatus	U41695
Trevesia baviensis	AF551731
Trevesia palmata	AF551735
Trevesia sundaica	AF551732
