# Supplementary material for: Extensive Pyrosequencing Reveals Frequent Intra-Genomic Variations of Internal Transcribed Spacer Regions of Nuclear Ribosomal DNA
Source: PLoS One. 2012 Aug 30;7(8):e43971. doi: 10.1371/journal.pone.0043971 (PMC3431384; doi:10.1371/journal.pone.0043971)
Supplement: Table S8 — Specific primer pairs for the corresponding species. (PDF) [file pone.0043971.s018.pdf]

Table S8. Specific primer pairs for the corresponding species.

Species	Specific Primer Name	Specific Primer Sequence (5’-3’)
Potentilla chinensis, *Potentilla nivea*	PCA_ITS2F	AACCCCTTCGGTGGCTGGA
	PCA_ITS2R	CGACGAATCGACACGCATTAGG
	PCA_5.8F	GCAGAATCCCGTGAACCATCG
	PCA_28SR	CGGTTCGCTCGCCGTTACTA
Citrus limonum	CL_5.8F	GCAGAATCCCGTGAACCATCG
	CL_ITS2R1	GATGCGAGCGCCGCTTGC
	CL_ITS2R2	GCTTGCGCGGAGCTTCAA
	CL_ITS2F	CTCGATCTCCGGCCGTGT
Eleutherococcus giraldii	ITS2-F	AACCCATCACTCCCTTGC
	28S-R	GTTCGCTCGCCGTTACTA
	5.8S-F	CAGAATCCCGTGAACCATC
