# Supplementary material for: Extensive Pyrosequencing Reveals Frequent Intra-Genomic Variations of Internal Transcribed Spacer Regions of Nuclear Ribosomal DNA
Source: PLoS One. 2012 Aug 30;7(8):e43971. doi: 10.1371/journal.pone.0043971 (PMC3431384; doi:10.1371/journal.pone.0043971)
Supplement: Table S9 — The PCR cycling and reaction conditions for specific primer pairs in Table S8. (PDF) [file pone.0043971.s019.pdf]

Table S9. The PCR cycling and reaction conditions for specific primer pairs in **Table S8**.

A. PCR cycling for *Eleutherococcus giraldii*.

	Temperature	Time
1 cycle:	94 ℃	5min
33 cycles:	94 ℃	30sec
	60 ℃	30sec
	72 ℃	30sec
1 cycle:	72 ℃	10min

B. Reaction conditions for *Eleutherococcus giraldii*.

Reaction Conditions	Volume (μL)
10× Standard *Taq* Reaction Buffer	2.5
dNTP(2.5mM each)	0.8
Forward primer(10μM)	0.6
Reverse primer(10μM)	0.6
NEB *Taq* DNA Polymerase(5U/μL）	0.1
DNA template (ca. 15ng/μL)	2.0
	Up to 25μL

C. PCR cycling for *Potentilla chinensis, Potentilla nivea, Citrus limonum*.

	Temperature	Time
1 cycle:	94 ℃	5min
33 cycles:	94 ℃	30sec
	65 ℃	30sec
	72 ℃	45sec
1 cycle:	72 ℃	7min

D. Reaction conditions for *Potentilla chinensis, Potentilla nivea, Citrus limonum*.

Reaction Conditions	Volume (μL)
2×PrimeSTAR® GC Buffer (Mg^{2+}plus)	12.5
dNTP(2.5mM each)	1
Forward primer(10μM)	0.6
Reverse primer(10μM)	0.6
PrimeSTAR® HS DNA Polymerase (2.5U/μL）	0.25
DNA template (ca. 15ng/μL)	2.0
	Up to 25μL
